# Essential Functional Interplay of the Catalytic Groups in Acid Phosphatase

**DOI:** 10.1021/acscatal.1c05656

**Published:** 2022-02-28

**Authors:** Martin Pfeiffer, Rory M. Crean, Catia Moreira, Antonietta Parracino, Gustav Oberdorfer, Lothar Brecker, Friedrich Hammerschmidt, Shina Caroline Lynn Kamerlin, Bernd Nidetzky

**Affiliations:** † Institute of Biotechnology and Biochemical Engineering, Graz University of Technology, NAWI Graz, Petersgasse 12/I, 8010 Graz, Austria; ‡ Austrian Centre of Industrial Biotechnology, Petersgasse 14, 8010 Graz, Austria; § Department of Chemistry—BMC, Uppsala University, BMC Box 576, S-751 23 Uppsala, Sweden; ∥ Institute of Biochemistry, Graz University of Technology, NAWI Graz, Petersgasse 12/II, 8010 Graz, Austria; ⊥ Department of Organic Chemistry, University of Vienna, Währingerstraße 38, 1090 Vienna, Austria

**Keywords:** functional cooperativity, enzyme catalysis, EVB simulations, linear free-energy relationship, nucleophilic catalysis, phosphate transfer

## Abstract

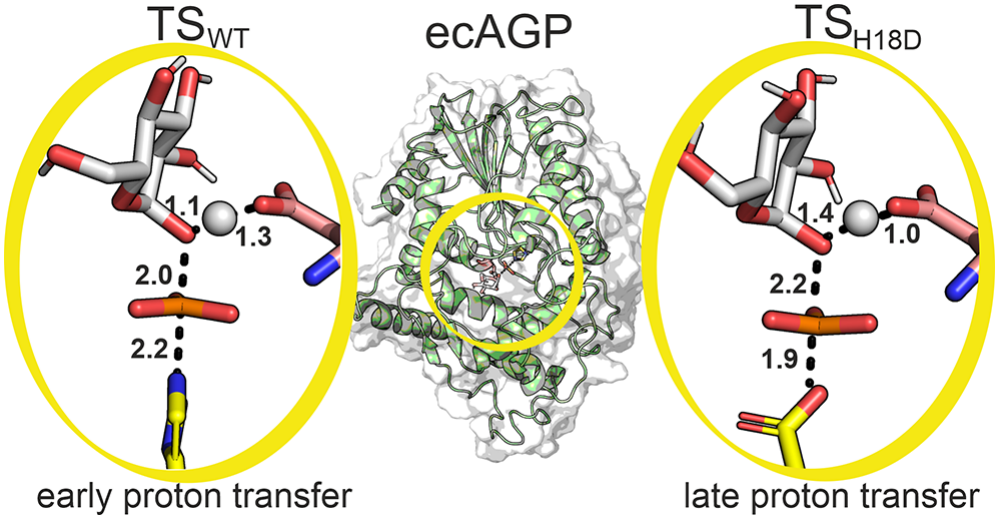

The cooperative interplay between the functional devices of a preorganized active site is fundamental to enzyme catalysis. An in-depth understanding of this phenomenon is central to elucidating the remarkable efficiency of natural enzymes and provides an essential benchmark for enzyme design and engineering. Here, we study the functional interconnectedness of the catalytic nucleophile (His18) in an acid phosphatase by analyzing the consequences of its replacement with aspartate. We present crystallographic, biochemical, and computational evidence for a conserved mechanistic pathway via a phospho-enzyme intermediate on Asp18. Linear free-energy relationships for phosphoryl transfer from phosphomonoester substrates to His18/Asp18 provide evidence for the cooperative interplay between the nucleophilic and general-acid catalytic groups in the wild-type enzyme, and its substantial loss in the H18D variant. As an isolated factor of phosphatase efficiency, the advantage of a histidine compared to an aspartate nucleophile is ∼10^4^-fold. Cooperativity with the catalytic acid adds ≥10^2^-fold to that advantage. Empirical valence bond simulations of phosphoryl transfer from glucose 1-phosphate to His and Asp in the enzyme explain the loss of activity of the Asp18 enzyme through a combination of impaired substrate positioning in the Michaelis complex, as well as a shift from early to late protonation of the leaving group in the H18D variant. The evidence presented furthermore suggests that the cooperative nature of catalysis distinguishes the enzymatic reaction from the corresponding reaction in solution and is enabled by the electrostatic preorganization of the active site. Our results reveal sophisticated discrimination in multifunctional catalysis of a highly proficient phosphatase active site.

## Introduction

Enzyme active sites are built from distinct functional devices that are derived from protein residues, the cofactors used, and sometimes the substrate itself.^1,2^ The basic role of each device in the catalytic cycle follows fundamental chemical principles and can usually be assigned with great clarity from experimental and computational evidence.^2−4^ However, the degree of functional interconnectedness of the different devices in determining the enzyme’s catalytic efficiency is by far less straightforward to establish.^5,6^ The extent to which the functionality of a single catalytic device is dependent upon the entire network of interactions within the preorganized structure of the active site is accessible to computational analysis,^7^ but it is fundamentally difficult to assess by experiment.^5,8^ However, the problem is central to the mechanistic understanding of the emergence of a broad diversity of active-site features among various contemporary enzymes that are completely unrelated by sequence, but catalyze identical chemical reactions.^2,9−14^ It is also central to the practical development of enzyme catalysts by de novo design^15,16^ and rational protein engineering.^16−18^ A systematic approach to its advance is to acquire in-depth knowledge about the interchangeability of different functional devices able to fulfill the same catalytic role within the given active-site environment of a natural enzyme.

**Figure 1:**
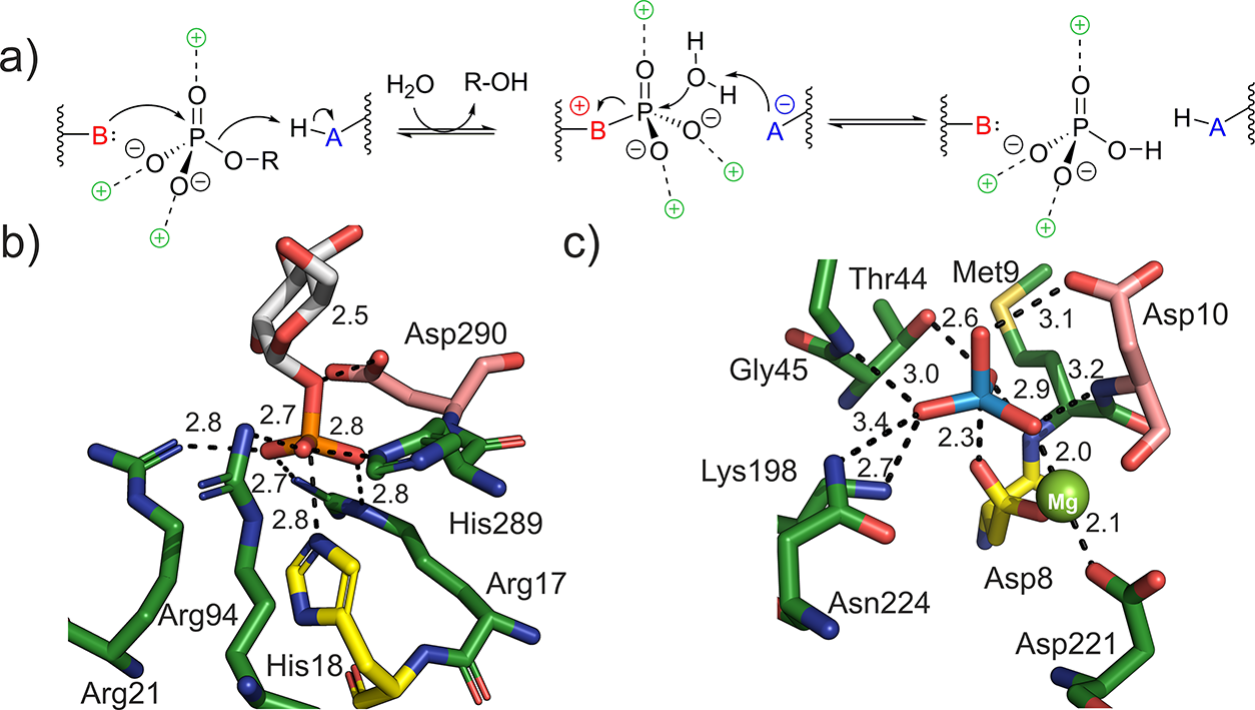
Covalent catalysis in the enzymatic hydrolysis of phosphomonoester substrates. (a) Proposed reaction mechanism, involving the interplay of a nucleophile (B), a general-acid–base (AH), and electrostatically stabilizing residues in enzymatic catalysis. Close-ups of the active sites of (b) acid phosphatase^30^ in complex with α-d-glucose 1-phosphate (Glc1P) and (c) the HAD-type phosphatase YidA^31^ in complex with tungstate, showing the nucleophile (His18 (b); Asp9, (c)) in yellow, the general-acid–base (Asp290 in (b); Asp10 in (c)) in pink, and the electrostatically stabilizing residues in green. Key distances (Å) are highlighted. (b) Model of the His18-containing wild-type phosphatase based on the crystal structure of the H18A variant of the enzyme (PDB ID: 1NT4).^30^ The structure shows the Michaelis complex with α-d-glucose 1-phosphate obtained in empirical valence bond (EVB) simulations of the enzymatic reaction, as described later in this study. Note: the tungstate in (c) is from a structural overlay of YidA (PDB ID: 1RKQ)^31^ and a tungstate complex of the hexose 6-phosphate phosphatase from *Bacteroides thetaiotaomicron* (PDB ID: 2RB5).^25^ The two enzymes have highly similar overall structures (residual mean-square deviation for the C_α_ atoms: 1.264 Å) and show superimposable active sites. For clarity, the residues of 2RB5 are hidden and only the tungstate is shown. The YidA structure shows the active-site Mg^2+^, which is typical of HAD-type phosphatases.

Here, we addressed the important case of nucleophilic catalysis^2,19^ and studied it in the context of enzymatic phosphoryl transfer.^20−23^ Nucleophilic catalysis involves the obligatory formation of a covalent enzyme intermediate.^2,19^ It is a common strategy of enzymes to subdivide the catalytic reaction into several, more easily manageable steps.^2,19^ A large class of enzymatic phosphoryl transfer reactions involve phospho-enzyme intermediates (Figure 1a) and use protein residues as nucleophiles.^13,20−29^ Here, we analyzed the substitutability of the native-histidine nucleophile by an alternative aspartate nucleophile in an acid phosphatase active site. We show that the aspartate enzyme involves participation from nucleophilic catalysis during the catalyzed phosphoryl transfer reaction that is mechanistically analogous to that observed in the wild-type histidine enzyme. We thus demonstrate that there is considerable flexibility of the overall phosphatase active site to accommodate different chemical groups for catalysis from a suitably positioned enzyme nucleophile. The aspartate enzyme however showed substantially (10^5^-fold) decreased activity compared to the native-histidine enzyme. We present evidence from experimental and computational studies showing that the large catalytic advantage of the histidine enzyme over the aspartate enzyme is not only an expression of an intrinsic reactivity difference between the two nucleophiles in phosphoryl transfer. It also reflects the essential functional interconnectedness between nucleophilic and general-acid catalysis during conversion of the phosphomonoester substrate into the covalent phospho-enzyme intermediate of the overall phosphatase reaction.

The biological task of hydrolyzing phosphomonoester substrates is handled by a group of phosphatases that represent a fascinating diversity of enzyme active sites.^10,21,23^ Mechanistically, these active sites can be classified according to whether they form a phospho-enzyme intermediate. Nucleotide hydrolases such as the GTPases do not use the catalytic intervention from an enzyme nucleophile.^32^ Many phosphatases however use covalent catalysis. Despite being structurally unrelated (Figure S1 and Table S1),^10^ the “phospho-enzyme” active sites have a mechanistic commonality in that they use a three-pronged strategy in catalysis (Figure 1a). Key elements of this strategy are the precise positioning of the phosphate group in the binding pocket to provide transition-state stabilization; the facilitation of leaving group departure (P–O bond cleavage) by electrostatic stabilization from a divalent metal ion or through partial protonation from a general acid; and the catalytic participation from a suitably activated enzyme nucleophile.^10,21,23,29^

For the purpose of this study, we considered a structurally simple phosphatase active site, that of histidine acid phosphatase, which lacks metal cofactors and involves a catalytic nucleophile (histidine) that does not require partner residues for its chemical activation (Figure 1a,b and Table S1).^30,33^ We replaced the relevant His18 in α-d-glucose 1-phosphate phosphatase from *Escherichia coli* (ecAGP; Figure 1b) by an aspartate which is the catalytic nucleophile in phosphatases of the haloalkanoate dehalogenase (HAD) superfamily (Figure 1c and Table S1).^25^ Among the HAD-type phosphatases in *E. coli*, YidA (alternative name: HAD13) and YihX (alternative name: HAD4) are highly active with the ecAGP substrate α-d-glucose 1-phosphate (Glc1P).^31^ Chemically^19,34−36^ and structurally,^10^ the ionized aspartate represents an excellent substitute of the histidine for function as the catalytic nucleophile under neutral pH conditions. In addition, the proposed catalytic reaction of the Asp18-containing ecAGP can be compared to the reversible isomerization (Glc1P to d-glucose 6-phosphate) catalyzed by β-phosphoglucomutase (βPGM), which has been characterized extensively both experimentally^37−41^ and computationally.^37,41−46^ However, we note that the Asp nucleophiles of PGMs and HADs cooperate in catalysis with a Mg^2+^ cofactor (Figure 1c) that is essential in providing electrostatic stabilization. Difference in the electrostatic environment between free and Mg^2+^-paired Asp may limit the mechanistic analogy regarding the function as catalytic nucleophile.

Based on a combined experimental-computational study, we have here performed a comparative mechanistic analysis of the wild-type and the H18D variant of ecAGP. We present evidence of residue interchangeability with retention of the basic role in nucleophilic catalysis. However, we find strong perturbation of the general-acid catalytic function of Asp290 in the H18D variant. This importantly demonstrates the fundamentally cooperative nature of the ecAGP active site and shows the essential interconnectivity of its catalytic devices in providing the impressive amount of rate acceleration (≥10^17^-fold)^21,47,48^ to phosphoryl transfer by the native enzyme.

## Results

### Covalent Catalysis to Phosphoryl Transfer by Histidine and Aspartate-Enzyme Nucleophiles

The H18D variant of ecAGP was constructed to place an alternative catalytic nucleophile into the enzyme active site (Figure 1b). Two separate triplet codon exchanges (CAC → GAC, CAC → GAT) were made to implement the same His18 → Asp substitution. Since the encoded proteins are identical, the obtained H18D enzymes must be experimentally indistinguishable. H18D activity due to translational misincorporation of histidine under the reversion of wild-type phenotype is extremely unlikely to have occurred similarly in both variant enzymes. Additionally, codon exchange at the first (nonwobble) base makes the Asp → His mistranslation an extremely improbable event. The D290A ecAGP was constructed to remove the catalytic general-acid–base. The H18D/D290A double variant combined the features of active site change. The H18A variant was constructed to remove any catalytic facilitation from an enzyme nucleophile.

Using Glc1P as a substrate for phosphomonoester hydrolysis, the purified H18D variants were about 10^5^-fold less active than the wild-type enzyme (*k*_cat_ = 35 ± 2 s^–1^; Table S2). The *K*_M_ for Glc1P (∼90–120 μM) was similar for wild-type enzyme and the H18D variants. Within the limit of experimental error, the H18D activity was independent of the genotype used (Table S2). Unless mentioned otherwise, the H18D variant with GAC codon genetic background was used in further experiments. The D290A variant showed ∼10^3^-fold decreased activity (*k*_cat_) and ∼10^4^-fold decreased efficiency (*k*_cat_/*K*_M_) compared to the wild-type enzyme (Table S2). Catalytic impairment of the D290A variant was understood from the requirement for general-acid assistance to the departure of the alcohol as leaving group at C1 (p*K*_a_ = ∼12) of the released glucose. The H18D/D290A double variant was just ∼5-fold less active than the H18D variant (Table S2). The result suggests that consequences of substituting the nucleophile and removing the acid–base were not energetically additive (i.e., multiplicative on the rate) in the double variant compared to the corresponding single variants. Non-additive energetics of double residue substitution suggests interdependent function of the two residues in enzyme catalysis. The H18A variant showed a large drop in catalytic ability, but was not completely inactive (Table S2). Its apparent *k*_cat_ of ∼1 × 10^–7^ s^–1^ provided a reference for enzymatic activity in phosphoryl transfer from Glc1P directly to water, without the formation of a phospho-enzyme intermediate. Note that nonenzymatic hydrolysis of Glc1P was not detectable within limits of detection of the methods used.

To demonstrate covalent catalysis in the H18D variant, we first sought to obtain direct evidence for the formation of a phospho-enzyme intermediate. Using a ^32^P-labeled phosphate group^26,49^ in the Glc1P substrate, we could clearly show the incorporation of radiolabel into the wild-type enzyme but did not observe the same for the H18D variant (Figure S2). The H18A variant did not incorporate radiolabel, as expected from the requirement of an enzyme nucleophile to form a phosphorylated enzyme. To examine the possibility that phosphorylation on Asp18 escaped detection by the method used, we analyzed a natural aspartate-phosphatase (YidA) as a reference. Although YidA converted Glc1P efficiently (*k*_cat_ = 20 ± 1 s^–1^; *k*_cat_/*K*_M_ = 9.5 × 10^4^ M^–1^ s^–1^), incorporation of the radiolabel was not detected (Figure S2). The stability of the aspartyl-phosphate in the enzyme might be too low to isolate.^49,50^ We therefore applied chemical reduction with sodium borohydride to convert aspartyl-phosphate into the nonlabile homoserine (Figure S3a) and analyzed the enzyme by tryptic peptide mass fingerprinting.^49^ For YidA incubated with Glc1P, indeed, the expected peptide containing the catalytic nucleophile Asp9 present as homoserine was found in high abundance (Figure S3 and Table S3). The same peptide was not detectable for the control (YidA incubated in the absence of Glc1P, Table S4). This result shows the formation of a phospho-enzyme intermediate on Asp9 in YidA and validates the analytical procedure used.

When applied to the H18D variant incubated with Glc1P, the analogous analysis succeeded in detecting, at low abundance, three unique peptides that had a homoserine at position 18 (Figures S3 and S4 and Table S5). The same peptides were absent from the sample of apo-H18D treated exactly identically in the absence of Glc1P (Table S6). The homoserine peptides were found with a peptide spectrum matches (PMS) score about 5-fold lower than the corresponding peptides containing the unmodified Asp18. The sodium borohydride treatment caused a small degree of unspecific conversion of Asp124, Asp333, and Asp383 into homoserine (Tables S5 and S6). The three Asp residues are located on the protein surface (Figure S5). Their conversion into homoserine occurred in both the H18D sample and control. Within the enzyme active site, however, the presence of homoserine was highly specific for Asp18. Importantly, Asp290, which is close to Asp18 in the crystal structure as shown later, was not converted into homoserine. These results are strong, direct evidence for the phosphorylation of Asp18 from Glc1P in the H18D variant. However, the portion of total enzyme that was phosphorylated at steady state appeared to be low.

### Transphosphorylation and Oxygen Isotope Exchange

When offered Glc1P as donor and glucose or mannose as acceptor, the H18D variant catalyzed transphosphorylation to give glucose 6-phosphate (Figure S6), or α-mannose 1-phosphate (Man1P, Figure S7) as products. Similarly, when Man1P was the donor and glucose the acceptor, the enzyme catalyzed transphosphorylation to give glucose 6-phosphate as the product (Figure S8). The wild-type enzyme catalyzed the same transphosphorylation reactions.^48,51^ The H18A variant was inactive as expected (Figure S6c).

Using ^18^O-labeled water (94%), we analyzed with ^31^P NMR spectroscopy the exchange of ^18^O between phosphate and solvent.^52^ Both the wild-type and the H18D variant catalyzed this exchange, as shown in Figure S9. The enzymatic exchange rates (wild-type: 0.24 s^–1^; H18D: 2 × 10^–5^ s^–1^) appeared to be consistent with the corresponding hydrolysis rates.

In accordance with the proposed catalytic mechanism of ^18^O isotope exchange (Figure S9), variants of ecAGP having the catalytic nucleophile (His18) or the catalytic acid–base (Asp290) replaced by an incompetent alanine residue were no longer able to catalyze the isotope exchange. The H18A variant cannot form the phospho-enzyme intermediate which is an essential requirement for the isotope exchange. Note: the mechanistic role of the Asp290 residue is discussed later in more detail. In summary, therefore, transphosphorylation and isotope exchange data provide further evidence for a phospho-enzyme intermediate on Asp18 in the H18D variant.

### Stereochemical Course of Phosphoryl Transfer

Transphosphorylation via a phospho-enzyme intermediate involves two steps with inversion of configuration, resulting in net retention of configuration at the phosphorus atom.^53^ We prepared (*R*_P_)- and (*S*_P_)-[^16^O,^17^O,^18^O]phosphoenolpyruvate (PEP) as two complementary substrates to unravel the stereochemical course of the enzymatic phosphoryl transfer. The synthetic route used was reported recently,^54^ but we here improved substantially on its overall efficiency (Sections S1.20–S1.27). Although PEP was hydrolyzed by both wild-type enzyme (*k*_cat_ = 0.8 s^–1^) and the H18D variant (*k*_cat_ = 6 × 10^–6^ s^–1^), it was not a usable phosphate donor substrate for transphosphorylation of glucose. The Glc6P product required for stereochemical analysis was obtained in yields (≤5%) too low to isolate. We therefore considered Man1P as an established donor substrate for both wild-type enzyme and the H18D variant (Figure S8).

**Figure 2:**
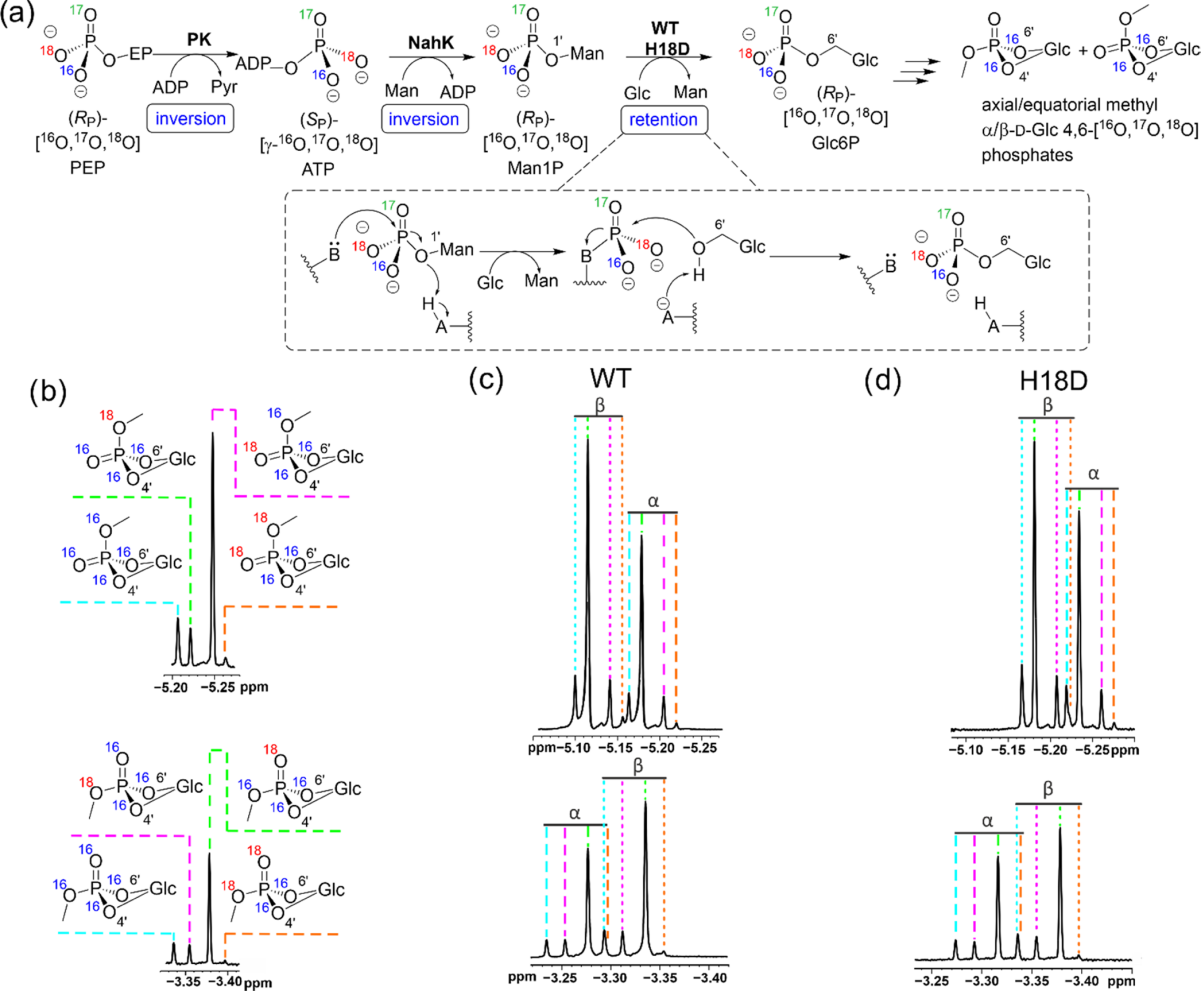
Stereochemical analysis of phosphoryl transfer catalyzed by wild-type and H18D forms of ecAGP. (a) Analytical principle, starting from (*R*_P_)-[^16^O,^17^O,^18^O]PEP to give the correspondingly chiral Man1P donor for enzymatic transphosphorylation to glucose, is shown. The Glc6P thus produced is derivatized via cyclization and methylation and is then analyzed by ^31^P NMR. (b–d) The four detectable ^31^P NMR signals (not including ^17^O-labeled products, as ^17^O relaxation causes large line widths at half height for all NMR signals) of α/β-d-glucopyranose-4,6-[^16^O,^17^O,^18^O]phosphate methyl esters are shown. Doubly labeled (2× ^16^O or 2× ^18^O) cyclic phospho-methyl-esters originate from a small amount of nonuniform isotope labeling during [^16^O,^17^O,^18^O]PEP synthesis and phosphoryl transfer from ATP (Figures S11 and S12). The signals of α- and β-configured products are indicated with dashed and dotted lines, respectively. Further details are provided in Section S1.31 in the Supporting Information, as well as in Figures S11–S14 and Tables S7 and S8.

We prepared Man1P from *P*-chiral (*R*_P_)- and (*S*_P_)-PEP, as shown in Figure 2a (Figure S10a). The phosphoryl transfer from PEP to mannose occurs in two enzymatic steps, each of which is known to proceed with inversion of configuration.^54,55^ The Man1P thus obtained has the same (*R*_P_)- or (*S*_P_)-configuration at the phosphorus atom as the *P*-chiral PEP substrate used. (*R*_P_)- or (*S*_P_)-Man1P (10 mM) was reacted with glucose (800 mM) in the presence of wild-type enzyme (0.07 μM; 60 min) or H18D variant (22 μM; 8 days). The *P*-chiral Glc6P samples formed (≥1.5 mM) were purified, converted to the corresponding cyclic methyl 4,6-phosphates and analyzed by ^31^P NMR spectroscopy. The results are summarized in Figure 2b–d for reactions using (*R*_P_)-Man1P and in Figure S10b–d for reactions using (*S*_P_)-Man1P. Transphosphorylation by the wild-type enzyme retained the stereochemistry at phosphorus as expected. Transphosphorylation by the H18D variant also proceeded with net retention of stereochemistry, as clearly shown in Figures 2 and S10–S14 as well as in Tables S7 and S8.

### Crystal Structure of the H18D Variant

**Figure 3:**
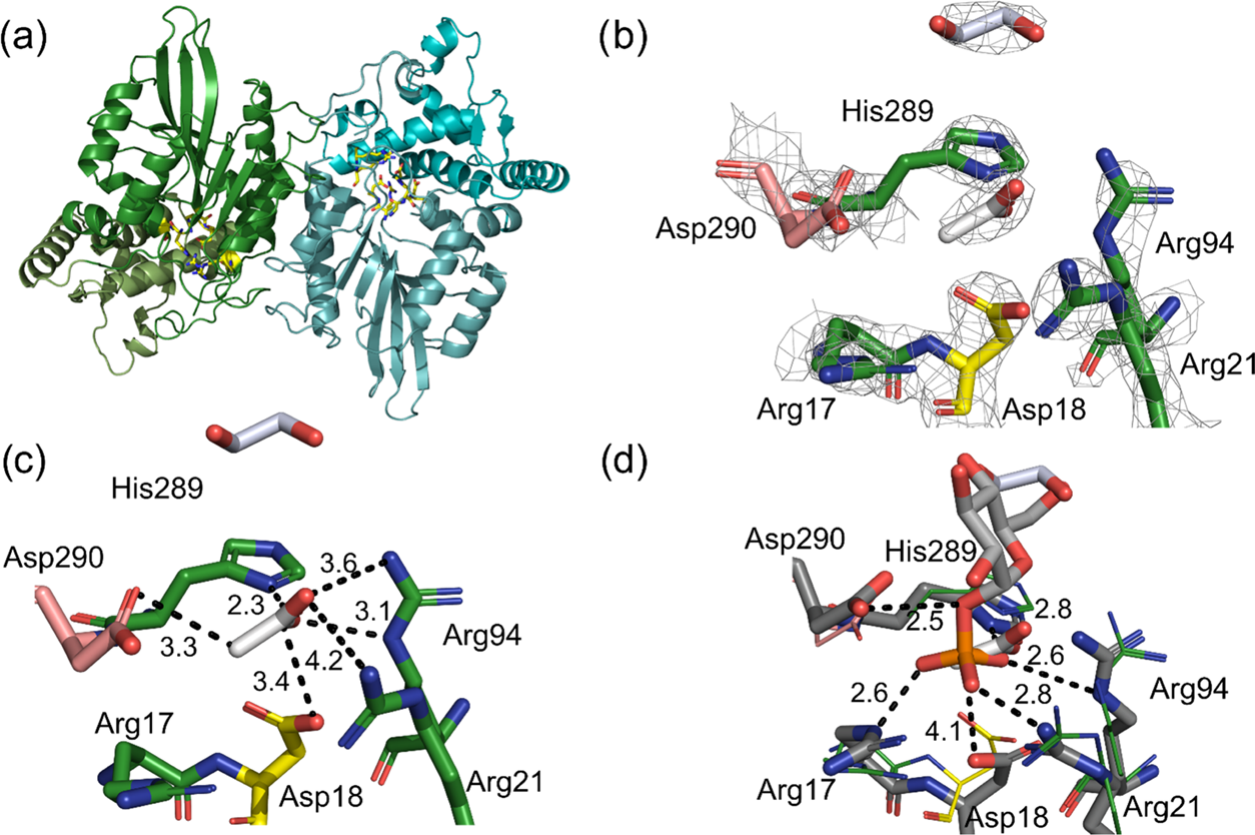
Crystal structure of the H18D variant with acetate bound in the active site. (a) Overall fold of the dimeric enzyme found in the asymmetric unit. The active site (highlighted in yellow) is at the interface of the Rossman fold core domain and the α helical cap. Close-ups of the active site, with (b) the 2*F*_O_–*F*_C_ electron density map of the structure contoured at 1.5 σ and (c) distances to the acetate ligand shown in Å. The Asp18 is highlighted in yellow. (d) Active-site overlay of the H18D–acetate complex (crystal structure; same colors as in (c)) and the H18D-Glc1P complex (empirical valence bond simulations; carbons of enzyme and Glc1P in dark and light gray, respectively).

The H18D variant was crystallized, and its X-ray structure was determined at 2.5 Å resolution (PDB ID: 6RMR). Data collection and refinement statistics are shown in Table S9. The protein crystal contained two enzyme molecules in the asymmetric unit. The functional enzyme is a homodimer with monomers arranged side by side in an opposite up-and-down orientation (Figure 3a). Both subunits adopt the characteristic histidine acid phosphatase fold,^33^ composed of a prominent α/β-domain to which a smaller α-helical domain is appended (Figure 3a). The active site is in an open cleft between the two structural domains. There are two active sites 32 Å apart from one another in the dimer structure (Figure 3a). Based on the structural evidence, therefore, we assume that the two active sites function independently in catalysis. Asp18 and the other active-site residues have well-defined electron density (Figure 3b). An acetate ion and ethylene glycol from the crystallization buffer are bound in and close to the active site, respectively (Figure 3b,c). Both molecules occupy positions that are occupied by Glc1P in the Michaelis complex (Figure 3d). The Michaelis complex was obtained by empirical valence bond (EVB) simulations of the Glc1P cleavage reaction catalyzed by the H18D variant, as described in the subsequent section. As shown in Figure 3c, the acetate is held in place by multiple interactions with the active-site residues. In the Michaelis complex (Figure 3d), a highly similar network of interactions is used to position the phosphate group of Glc1P. Therefore, Asp18 is well positioned for catalytic function as an enzyme nucleophile. Its side-chain O atom is 3.4 Å away from the C1 of acetate (Figure 3c). In the modeled Michaelis complex (Figure 3d), the distance between Asp18-O and the phosphate-P is 4.18 ± 0.07 Å.

### EVB Simulations of the Wild-Type Enzyme and the H18D Variant

We applied EVB simulations to study the Glc1P cleavage reaction to give the phospho-enzyme intermediate for both the wild-type ecAGP and the H18D variant. Comparison of our calculated activation free energies (Δ*G*^‡^) to the experimental data demonstrates that we are able to reproduce the dramatic loss in activity upon the H18D mutation (Table 1). The reaction free energies (Δ*G*^0^) are notably different for the two enzymes. Exothermicity of the H18D-catalyzed reaction may reflect a substantial reorganization penalty paid to achieve positioning for catalysis in this enzyme. MS data indicating the formation of the phospho-enzyme intermediate on Asp18 in extremely low amounts (Figures S3 and S4 and Tables S5 and S6) is consistent with impaired positioning in the H18D variant. While interesting, the Δ*G*^0^ of enzyme phosphorylation on His/Asp18 from Glc1P was not further pursued.

**Table 1:** Comparison between the Experimental and Calculated Activation (Δ*G*^‡^) and Reaction Free Energies (Δ*G*^0^) for the Dephosphorylation of Gc1P by the Wild-Type Enzyme and the H18D Variant^a^

enzyme	*k*_cat_ (s^–1^)	Δ*G*_exp_^‡^ (kcal mol^–1^)	Δ*G*_calc_^‡^ (kcal mol^–1^)	Δ*G*_calc_^0^ (kcal mol^–1^)
wild-type	35	16.0	17.6 ± 0.2	4.1 ± 0.4
H18D	2.0 × 10^–4^	23.4	22.1 ± 0.7	–7.3 ± 0.8

a The calculated energies are the averages and standard errors of the mean from 30 individual empirical valence bond (EVB) simulations, obtained as described in the Supporting Information. The activation free energies for the experimentally determined values were obtained using the Eyring equation.

**Figure 4:**
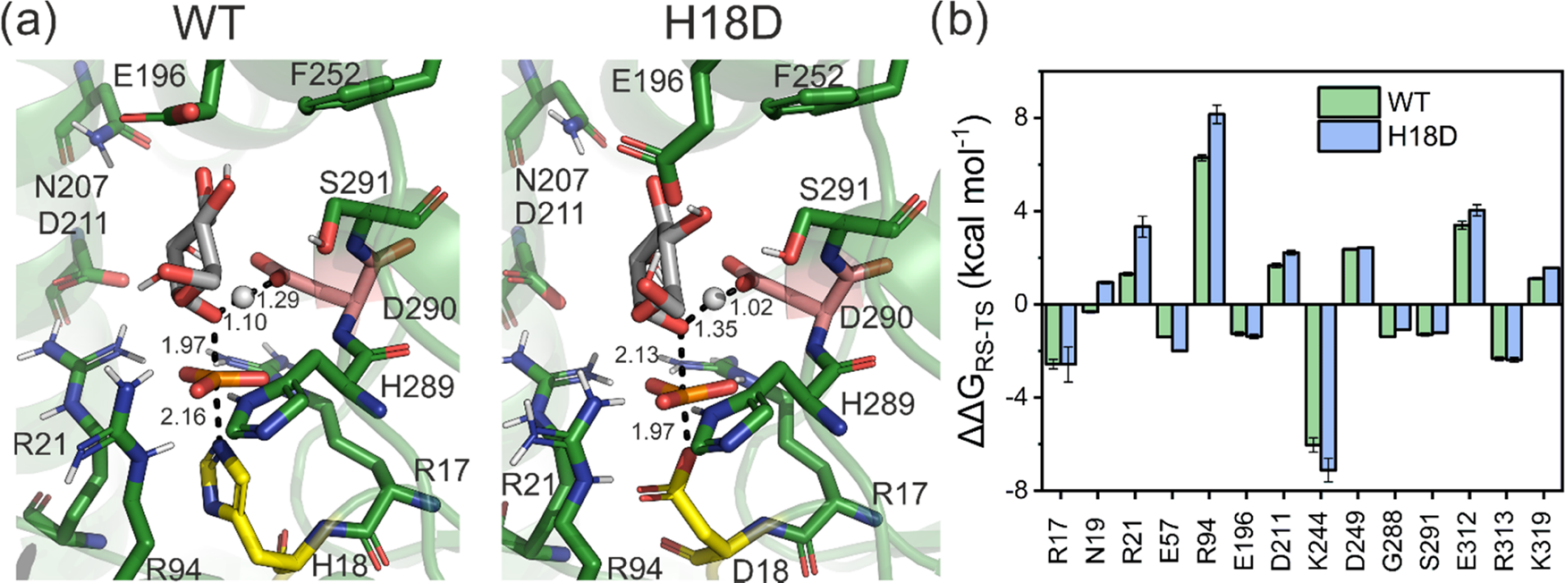
(a) Representative structures of the transition states for the dephosphorylation of G1c1P by (left) the wild-type enzyme and (right) the H18D variant. The nucleophilic and acid–base residues are shown in yellow and salmon, respectively. The structures shown here are the centroids of the top ranked cluster obtained from clustering the active site (described in Sections S1.33–S1.36, Supporting Information). (b) Per residue electrostatic contributions to the calculated activation free energies for both enzymes, where negative values indicate electrostatically stabilizing contributions and positive values indicate electrostatically destabilizing contributions. These values were obtained by applying the linear response approximation^56,57^ to the calculated EVB trajectories for the active site. Only residues with contributions of >1 kcal mol^–1^ are shown here for clarity. The corresponding raw data for (b) are shown in Table S13.

We therefore analyzed our EVB simulations to gain detailed insight into the chemistry behind the phosphorylation reaction catalyzed by both enzymes. Representative structures at key stationary points obtained from our EVB simulations for each of the enzyme-catalyzed reactions considered in this work are shown in Figures 4 and S15, and analysis of the corresponding hydrogen-bonding networks is shown in Tables S10 and S11. The average distances of the reacting atoms at the key stationary points along the reaction coordinate for the reactions catalyzed by each enzyme variant are shown in Table S12. As can be seen from Tables S10 and S11, we do not observe radical changes in the hydrogen-bonding interactions between the reacting atoms and the surrounding protein upon moving from the Michaelis complex to the transition state; however, we do see a slight increase in hydrogen-bonding interactions in the case of the wild-type enzyme (Table S10), whereas in the case of the H18D variant, we see a slight decrease in hydrogen-bonding interactions (Table S11), corresponding to a total difference of ∼2 hydrogen bonds between the two systems.

This is corroborated by the examination of the electrostatic interactions^56,57^ between the reacting atoms and the protein side chains (Figure 4b), which are very similar in the case of the two enzyme variants. However, although the contributions of individual residues remain similar, the sum of electrostatic contributions from all residues with contributions >0.5 kcal mol^–1^ (Table S13) is −0.3 kcal mol^–1^ in the case of the wild-type enzyme, and 4.6 kcal mol^–1^ in the case of the H18D variant. Therefore, the H18D variant provides less electrostatic stabilization to the transition state, of which 1.3, 2.0, and 1.9 kcal mol^–1^ comes from destabilization interactions with the Asn19, Arg21, and Arg94 side chains, respectively. In particular, the Arg21 and Arg94 side chains both interact with the charged phosphate group throughout the reaction, and the difference in electrostatic contributions to transition state stabilization between the wild-type and H18D variants suggests suboptimal positioning of these side chains during the reaction.

Comparison of average distances between reacting atoms at the transition states for both the enzymatic and nonenzymatic reactions (Table S12) indicates that the transition states for all reactions are relatively similar in the context of phosphorus–oxygen and phosphorus–nitrogen (as relevant) distances to the incoming nucleophile and departing leaving group.

**Figure 5:**
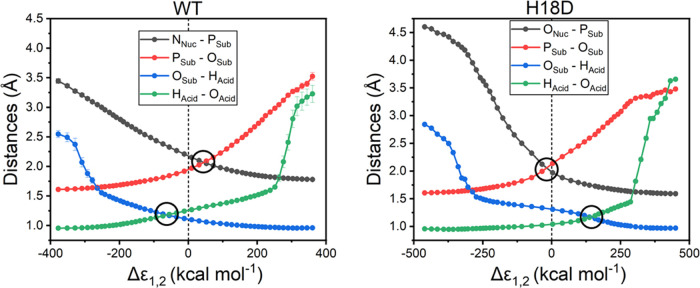
Change in the distances of key reacting atoms in the reactions catalyzed by the wild-type (WT) enzyme and the H18D variant, along the EVB energy gap reaction coordinate (Δε_1,2_). The approximate location of the transition state (Δε_1,2_ = 0) is marked on both graphs with a dotted line and the points at which the reacting atoms “cross-over” (i.e., phosphoryl and proton transfer occur) are circled for clarity. The analogous data for the corresponding nonenzymatic reactions is shown in Figure S16, and the calculated average reacting atom distances at the Michaelis complexes, transition states, and product complexes for all reactions are provided in Table S12. Note that the EVB calculations were performed using a two-state model that simultaneously describes proton transfer and phosphoryl group transfer; see Figures S24 and S25.

A larger difference is seen in the degree of proton transfer from the protonated D290 side chain to the leaving group oxygen. This is again corroborated by tracking how these distances change along the energy gap (Δε_1,2_) reaction coordinate used by the EVB approach (this reaction coordinate is defined as the energy difference between the EVB diabatic states, with the transition state located at Δε_1,2_ = 0 where the two states cross).^58,59^ This data is shown in Figures 5 and S16 for the enzymatic and nonenzymatic reactions, respectively, where the nonenzymatic reaction was modeled using either methyl imidazole or propionic acid as a nucleophile, as described in the Supporting Information. From this data (Figure S16), it can be seen that in the nonenzymatic reaction, phosphoryl group transfer occurs just before the EVB transition state, with proton transfer from propionic acid to the leaving group lagging behind the transition state. This is similar to what we observe in the case of the reaction catalyzed by the H18D variant (Figure 5). However, in the case of the wild-type enzyme, the enzyme environment changes the nature of the transition state toward a looser transition state with earlier proton transfer, and phosphoryl transfer occurring just after the EVB transition state. The looser transition state in the case of the wild-type enzyme (compared to the H18D variant) is likely facilitated by the fact that proton transfer to the leaving group is essentially complete by the transition state.

### Linear Free-Energy Relationship (LFER) Analysis

To obtain experimental evidence in support of the suggestion from our EVB simulations, that the proton transfer from Asp290 was affected by the His18 → Asp exchange of catalytic nucleophile, we considered LFER analysis. We determined kinetic parameters for the hydrolysis of a homologous series of phosphomonoester substrates, featuring a substituted or unsubstituted phenol as the leaving group (Table S14). The p*K*_a_ of the leaving group varied between 7.1 and 10.0 due to the effect of the substituent.^60^ Besides the wild-type enzyme, the H18D variant and the D290A variant were used. When using a substrate with a leaving group able to depart without the need for protonation (e.g., 4-nitrophenol, p*K*_a_ = ∼7.1), the D290A variant was even more active in terms of the *k*_cat_/*K*_M_ (∼10-fold) than the wild-type enzyme. The result supported the mechanistic idea of the D290A variant, which was the specific removal of acid–base catalytic function from an otherwise unperturbed enzyme active site.

**Figure 6:**
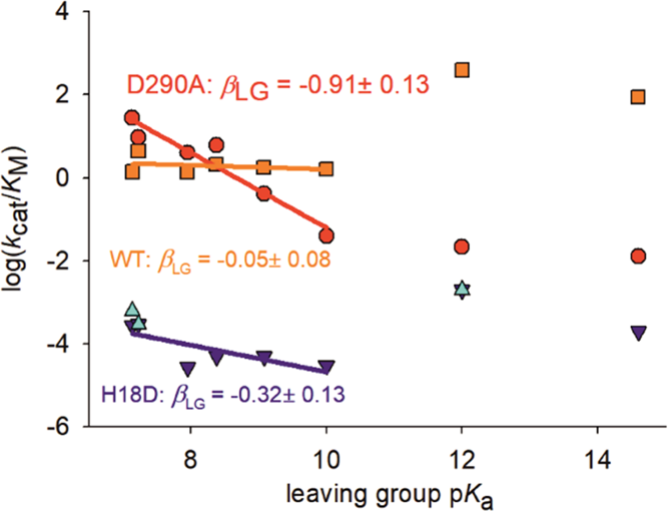
LFER analysis for the hydrolysis of substituted phenyl phosphate substrates catalyzed by the wild-type enzyme (orange rectangles), as well as the D290A (red circles) and H18D (dark blue triangles) variants and an H18D variant derived from a different genetic background (CAC → GAT codon exchange, cyan triangles). The *k*_cat_/*K*_M_ values for reaction with Glc1P (p*K*_a_ = ∼12) and Glc6P (p*K*_a_ = ∼15) are outliers in the plots and excluded from the calculation of the Brønsted β_LG_ coefficients.

In the two-step mechanistic pathway of the phosphatase (Figure 1a), *k*_cat_/*K*_M_ represents the phosphoryl transfer from the substrate to the enzyme nucleophile (His18, Asp18) as a kinetically isolated step. Note here that free 2-nitrophenol does not inhibit the enzymatic reactions, as shown in Figure S17. This suggests that phospho-enzyme complex with 2-nitrophenol does not accumulate at steady state. Reverse phosphoryl transfer from the enzyme to 2-nitrophenol is not significant under initial-rate conditions. This supports the simple kinetic mechanism used. The kinetic data for each enzyme is shown in a Brønsted plot that correlates the logarithmic *k*_cat_/*K*_M_ with the p*K*_a_ of the substrate leaving group. The Brønsted coefficient, β_LG_, is the slope of the LFER thus obtained (Figure 6). The β_LG_ of the wild-type enzyme is effectively zero (−0.05 ± 0.08); that of the D290A variant is −0.91 ± 0.13. The β_LG_ of the H18D variant lies between the two and has a value of −0.32 ± 0.13.

To compare the β_LG_ for wild-type enzyme and H18D variant, we considered the observation from studies of nonenzymatic phosphoryl transfer that the β_LG_ becomes less negative when the p*K*_a_ of the nucleophile increases, with a decrease of ∼0.013 for each increase in p*K*_a_ unit.^35^ The difference in side-chain p*K*_a_ for His and Asp is not large, ∼1.8 when the p*K*_a_ values in solution (His: ∼6.0; Asp: ∼3.9) are used. The correction on the β_LG_ of the H18D variant is +0.027 (=0.013 × 2.1), thus yields a corrected value of −0.293. These enzymatic β_LG_ values imply that the intrinsic differences in leaving group ability of the different phenols (given by their p*K*_a_) are effectively offset in the wild-type reaction while they are largely expressed in the reaction of the D290A variant. These data thus suggest that proton transfer from Asp290 to the oxygen atom of the leaving group is responsible for the equalization of leaving group reactivity in the wild-type reaction (Figure 6).

**Figure 7:**
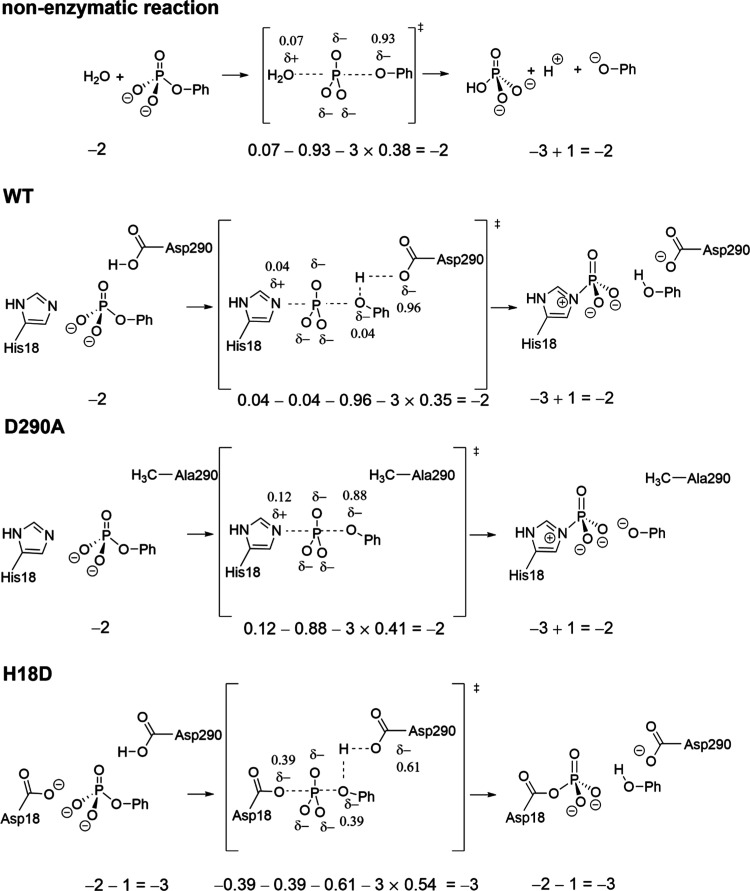
Hypothetical charge distribution at the transition states for phosphoryl transfer from phenyl phosphate to water in the nonenzymatic reaction and to His18/Asp18 in the enzymatic reactions, as calculated from the LFER data. The relationship β_LG_/β_EQ_ is used to calculate the charge. β_EQ_ is −1.35.^61^ We assume a symmetrical transition state with equal amounts of bond cleavage to the leaving group and bond formation to the nucleophile, consistent with previous analyses.^19,62,63^ In the nonenzymatic reaction and the reaction of the D290A variant that do not involve catalytic proton transfer to the leaving group oxygen atom, the positive charge on the attacking nucleophile can thus be calculated. In reactions involving catalytic proton transfer from Asp290, a total negative charge of 1 was assumed for the leaving group oxygen atom and the side chain of Asp290.

The difference in p*K*_a_ between Asp (∼3.9) and the leaving group phenol (≥7.1) is noted. The β_LG_ of the H18D variant indicates accordingly, that on substituting the enzyme nucleophile in the active site, the catalytic proton transfer to the leaving group is no longer as complete (Figure 7) as it is in the native phosphatase. In other words, the transition state of phosphoryl transfer by the H18D variant is suggested to involve negative charge development on the leaving group oxygen atom (0.27 = −0.293/–1.35 = β_LG_/β_EQ_; β_EQ_^61^ describes the sensitivity of the reaction equilibrium of hydrolysis of phosphomonoesters on the p*K*_a_ of the phenolic leaving group) which is lacking in the corresponding transition state for the wild-type (0.04 = −0.063/–1.35). The negative charge development on the leaving group oxygen atom in the reaction of the D290A variant is calculated as 0.88 (=–0.91/–1.35). These findings emphasize the coordinated interplay between nucleophile and general acid in the conversion of a phosphomonoester in the phosphatase active site, which is discussed below.

We note that the *k*_cat_/*K*_M_ values for reaction with Glc1P and Glc6P are positive outliers in the Brønsted plots for wild-type enzyme and H18D variant (Figure 6). Substrate binding that is somewhat specific for the glucose leaving group, and is utilized to promote catalysis, is the probable cause for the effect. Differences in substrate reactivity with the enzyme notwithstanding, the EVB simulations (reaction with Glc1P; Figure 5 and Table S12) and the LFER study (reaction with phenyl phosphates; Figures 6 and 7) give a detailed and coherent picture about the mechanistic consequences of nucleophile substitution in the H18D variant.

## Discussion

### Phospho-Enzyme Intermediate on Asp18

Evidence for a phospho-enzyme intermediate in the mechanistic pathway of the H18D variant comes from the following: (a) the enzyme catalyzes transphosphorylation with a kinetic behavior that is consistent with the formation of such an intermediate; (b) the enzyme catalyzes the exchange of ^18^O between water and phosphate; (c) stereo-chemically, the enzyme-catalyzed transphosphorylation proceeds with net retention at the phosphorus atom; and (d) the phosphorylated Asp18 was detected as homoserine in phospho-enzyme reduced by sodium borohydride. The complete absence of activity in the H18A variant furthermore shows that a nucleophilic residue at position 18 is required for enzymatic phosphoryl transfer. The formation of the phospho-enzyme intermediate on Asp18 is thus strongly supported. Asp18 substitutes the native His18 with retention of the original catalytic function. The anticipated role of His18 in the wild-type enzyme is well supported by previous evidence^30,33,47^ and further evidence provided by this study.

### Cooperativity between Nucleophilic and General-Acid Catalysis

The evidence for cooperative energetics between the nucleophilic and the general-acid catalytic groups in the ecAGP active site is the following: (a) There is a much larger (≥10^5^-fold) catalytic advantage for a histidine compared to an aspartate-enzyme nucleophile in reactions with substrates (e.g., Glc1P) requiring assistance from proton transfer to facilitate the departure of their leaving group than in reactions with substrates (e.g., 4-nitrophenyl phosphate) not requiring such assistance and (b) the extent of proton transfer from Asp290 to the leaving group in the transition state of phosphoryl transfer between substrate and enzyme is considerably lower for the aspartate compared to the histidine nucleophile (Figures 5 and 6). The *k*_cat_/*K*_M_ values for conversion of 4-nitrophenyl phosphate by the wild-type enzyme (1.39 × 10^3^ M^–1^ s^–1^) and the H18D variant (0.27 M^–1^ s^–1^) show that, as an isolated factor of ecAGP catalytic efficiency, the advantage of a histidine compared to an aspartate nucleophile is ∼10^3^-fold. In nonenzymatic phosphoryl transfer reactions, the advantage of nitrogen over oxygen nucleophiles was between 30- and 100-fold.^35,36,62,64^ The lowering of *k*_cat_/*K*_M_ in the H18D variant compared to the wild-type enzyme might therefore involve the effect of this intrinsic difference in nucleophilic reactivity. However, there are additional factors important in the enzyme specifically. The *k*_cat_/*K*_M_ values for conversion of Glc1P (wild-type: 3.9 × 10^5^ M^–1^ s^–1^; H18D: 2 M^–1^ s^–1^) show that the essential cooperativity between nucleophilic and general-acid catalysis adds another ∼10^2^-fold to the advantage of the histidine nucleophile. The marked change in the β_LG_ slope of Figure 6, from −0.05 for the wild-type enzyme to −0.32 for the H18D variant, suggests that the catalytic proton transfer is strongly modulated dependent on the nucleophilic group present in the enzyme active site. In this respect, the catalysis by the enzyme differs strikingly from the catalysis in solution (cf. Figures 5 and S16). There is little interplay between the general acid and the nucleophile in nonenzymatic catalysis of phosphomonoester hydrolysis in solution.^19,35^

Interpretation of both the EVB simulations and the LFER analysis offers additional insight. From our EVB simulations (Figures 5 and S16), it can be seen that the nonenzymatic transition states for reactions involving either methyl imidazole or propionic acid as a nucleophile are geometrically very similar, with relatively synchronous phosphoryl transfer and protonation of the leaving group lagging the transition state. A similar mechanistic trend is seen in the reaction catalyzed by the H18D variant. However, in the case of the reaction catalyzed by the wild-type enzyme (histidine nucleophile), the transition state shifts to an early transition state with essentially full proton transfer to the leaving group preceding phosphoryl transfer. The enzyme forms slightly more stabilizing interactions with this transition state (Tables S10 and S11), and the Michaelis complex is slightly better preorganized for optimal stabilization of phosphoryl transfer, in particular by Asn19, Arg21, and Arg94 (Figure 4 and Table S13).

For comparison, the β_LG_ for wild-type and D290A phosphatases is indicative of a loose (dissociative) transition state (Figure 6) in which the P–O bond to the leaving group oxygen atom is largely broken (both enzymes) and the catalytic proton transfer to the leaving-group oxygen atom far advanced (wild-type enzyme). Assuming a somewhat symmetrical transition state, consistent with previous studies of phosphoryl transfer in chemical^19,21,35,63,65^ and enzymatic systems,^23,62^ there would be also little bond formation to the nitrogen atom of the histidine nucleophile, as shown in Figures 4 and 5 and Table S12. In the wild-type enzyme, therefore, the development of negative charge associated with that transition state would be mainly on the side-chain carboxylate of Asp290 while in the D290A variant it would be on the leaving-group oxygen atom. In the H18D variant, there is a −1 extra charge from the enzyme nucleophile. As illustrated in Figure 7, this extra charge might be better accommodated in a transition state somewhat tighter than that of the wild-type enzyme.

A relatively lower degree of P–O bond breaking to the leaving-group oxygen atom in the H18D transition state, as predicted by our EVB simulations (Figure 5), would be consistent with the suggestion from the LFER study that the catalytic proton is only partially transferred from Asp290 to the leaving-group oxygen. This in turn might result in an increase in nucleophilic participation from Asp18 compared to that of His18. The requirement for Asp290 to go from being protonated in the complex with phosphomonoester substrate to being unprotonated in the phospho-enzyme intermediate suggests considerable sensitivity of its p*K*_a_ to the electrostatic environment of the active site. Substantial negative charge (Asp18) compared to a small positive charge (His18) on the attacking nucleophile in the enzymatic transition state (Figure 6) might be the reason for the restricted extent of proton transfer from Asp290 to the leaving group oxygen atom in the H18D transition state. Together, both effects (steric and intrinsic cooperativity) offer a plausible explanation for the reduced catalytic proficiency observed in H18D.

### Implications for Enzymatic Phosphoryl Transfer

Phosphoryl transfer reactions have been extensively investigated for many decades^3,12,20,62,63,66−68^ due to both the importance they have in biology and the chemical challenges they present.^67,68^ The results of this study of ecAGP converge with earlier mechanistic inquiries into the suggestion of a dissociative transition state of enzymatic phosphoryl transfer^21,63,69^ and refute computational models predicting an associative transition state in the histidine acid phosphatase superfamily.^70,71^

The cooperative nature of the interactions employed in enzymatic transition-state stabilization is the new insight of fundamental importance. Active-site remodeling to replace the native catalytic nucleophile His18 by aspartate, with retention of function in the enzymatic mechanism, was central to this study. Nucleophilic residues have been replaced in phosphatases^29,47,49,71−73^ as well as in other phosphotransferase and nucleotide transferase enzymes,^74−77^ but the resulting variants were usually unable to perform the full catalytic cycle.

Enzymatic reactions differ fundamentally from nonenzymatic reactions in their use of catalysis from a well-positioned (instead of a free) nucleophile. An enzyme variant whose activity is enhanced by binding of an exogenous nucleophile to the protein cavity vacated by site-directed substitution of the native nucleophilic residue by a small non-nucleophilic residue, typically a glycine, presents an instructive surrogate of the native enzyme for examining the catalytic importance of the positioned nucleophile.^74−76^ However, it is fundamentally difficult to mimic in a noncovalent enzyme/nucleophile complex the effect of positioning that derives from the nucleophilic residue’s being an integral part of the folded polypeptide structure. Therefore, the distinct advance made herein is that a defined change in the nucleophilic residue positioned for reaction in the enzyme active site was achieved and the effect of this change on enzymatic reactivity could be elucidated.

We note that the current study of histidine acid phosphatase has some conceptual precedent in early mutagenesis studies of alkaline phosphatase.^72,73,78,79^ The native nucleophile (Ser-102) of *E. coli* alkaline phosphatase was individually replaced by Cys, Leu, and Ala.^29,72,78^ The S102C variant was reported to catalyze transphosphorylation with retention of stereochemistry at the phosphorus atom.^79^ However, despite having a nonfunctional residue at the site of the original nucleophile, the S102L variant was still able to catalyze transphosphorylation and both the S102L and S102A variants were reasonably active phosphomonoesterases (although (4–10) × 10^2^-fold less so than the wild-type enzyme).^72,80^ The crystal structure of the S102C variant showed Cys102 to be ligated to one of the active-site zinc ions which the original serine was not.^78,81^ Therefore, caution is warranted to assume that the S102C variant utilizes the exactly analogous chemical mechanism as the wild-type alkaline phosphatase.

The β_LG_ data for wild-type ecAGP and H18D variant suggests that proton transfer to the departing leaving group decreases as a consequence of the substitution of the uncharged histidine nucleophile by the negatively charged aspartate (Figure 6). An increase in nucleophilic participation enforced in that way might render the transition state somewhat more associative in nature. There is good evidence from both enzymatic and nonenzymatic studies, however, that phosphate monoester hydrolysis catalyzed by anionic oxygen nucleophiles react through dissociative transition states.^35,62,63,65^ Reversal of causality, namely that the increased nucleophilic participation from Asp18 compels a lowered degree of proton transfer to the leaving group, is thus rated unlikely.

Considering that general-acid catalysis is most effective in transition states involving only partial proton transfer,^19^ the interesting question arises as to why the H18D variant is particularly catalytically impaired (relative to the wild-type enzyme) in reactions with high-p*K*_a_ leaving groups that would benefit most from general-acid catalysis. Herschlag and Jencks^35^ have commented that, setting aside differences in entropic barrier to the reaction for associative and dissociative transition states, the disadvantage of the associative reaction is that it does not allow for the release of electron density from the phosphate oxygen atoms to the expulsion of the leaving group. In an enzyme-catalyzed dissociative reaction accordingly, one might expect cooperative energetics between general-acid catalysis and electrostatic stabilization to drive this electron density release for the development of the metaphosphate-like species in the transition state. In the H18D variant, therefore, the alteration forced upon the general-acid catalysis due to the presence of the Asp18 nucleophile plausibly results in a significant loss in electrostatic stabilization.

The idea that general-acid catalysis, functionally interconnected with electrostatic stabilization, shapes the character of the transition state converges well with current mechanistic thinking on enzymatic phosphoryl transfer.^19,62,82^ It is consistent in particular with findings from kinetic isotope effect studies of protein tyrosine phosphatase, showing that removal of an electrostatically stabilizing arginine through residue change into alanine caused perturbance of the catalysis from the general acid (aspartic acid).^83,84^ This, in turn, caused the originally dissociative transition state to become somewhat more associative, similarly to when the general-acid catalysis was inactivated directly. Simultaneous removal of the two residues in a D356N/R409A double variant of the phosphatase reinstalled the original dissociative character of the transition state.^83^ In bringing the catalytic nucleophile into the overall picture, our analysis represents an important step toward the complete apprehension of the cooperative energetics in phosphatase active sites. The central role of the general-acid catalyst in interconnecting the catalytic functions of the nucleophile and the apparatus for electrostatic stabilization^83^ is thus made evident.

### Implications for Enzyme Engineering and Design

Progress in the design and improvement of enzyme catalysts is a major practical and fundamental challenge.^6,15,85^ Computational design of enzymes has made important advances in this area.^86^ A high degree of sophistication is required of a protein site capable of efficient catalysis. The catalytic devices in the active sites of natural enzymes are functionally interconnected.^5,6,87−89^ Understanding this interconnection in relation to the overall catalytic effectiveness is fundamental to the design of new enzyme active sites.

The reconstruction of nucleophilic catalysis in ecAGP reported here extends beyond traditional mechanistic studies and informs enzyme (re)-design in two important ways. First, it demonstrates functional resilience of the natural active site toward substitution of the original catalytic residue (His18). Limits of resilience delineate the relevant design space for the particular catalytic apparatus considered. Second, it suggests that phosphoryl transfer from the monoester substrate to the enzyme is facilitated by two distinct catalytic modules, one for nucleophilic catalysis and another for electrostatic stabilization. The two modules are functionally interconnected by the general-acid catalytic residue. The catalytic proton transfer to the leaving group modulates the character of the transition state.

The obligatory “p*K*_a_ cycling” of the acid–base residue in the enzymatic reaction during formation and hydrolysis of the phospho-enzyme intermediate (Figure 6) places special demands on the electrostatic complementarity between the acid–base and the nucleophile for their well-orchestrated, cooperative function. Mechanistic characterization of the native-histidine ecAGP and its redesigned-aspartate counterpart thus reveals, and quantitatively delineates, the essential interplay between the core residues of their catalytic apparatus. The relative simplicity of the ecAGP active site has enabled this interplay to be examined largely unmasked from the influence of extended interaction networks into which the core catalytic residues can be embedded in other enzymes (e.g., alkaline phosphatase^87^). Thus, basic requirements, or general design criteria, of a solely protein-derived, minimal catalytic site for proficient phosphoryl transfer are suggested. Here, our study emphasizes the fine level of structural and electronic discrimination^1,5,62,63,90,91^ that a designed phosphatase active site would have to show to rival the catalytic effectiveness of the natural enzyme.

## Materials and Methods

A detailed description of the methods used is given in the Supporting Information.

## Supporting Information Available

Preparation of ecAGP and auxiliary enzymes (Sections S1.2–S1.7); assays used (Section S1.8); crystallization and structure determination (Sections S1.9 and S1.10 and Table S9); kinetic studies and LFER analysis (Sections S1.11 and S1.12 and Table S14); inhibition of enzymatic hydrolysis of 4-nitrophenyl phosphate by 2-nitrophenol (Section S1.13 and Figure S17); isotope exchange studies (Section S1.14 and Figure S9); phospho-enzyme intermediate trapping studies (Sections S1.15–S1.18, Figures S2–S5, and Tables S3–S6); preparation of aryl phosphates (Section S1.19 and Figures S19–S23); synthesis of (*R*_P_)- and (*S*_P_)-[^16^O,^17^O,^18^O]PEP (Sections S1.20–S1.27); stereochemical analysis of enzymatic phosphoryl transfer (Sections S1.28–S1.32, Figures S10–S14, and Tables S7 and S8); and EVB calculations (Sections S1.33–S1.36, Figures S15, S16, and S24–S26, and Tables S10, S13, and S15) (PDF)

## Notes

Coordinates and structure factors of H18D were deposited in the RCSB Protein Data Bank (6RMR).

## Notes

All EVB parameters, input files, and starting structures necessary to reproduce our work are available at the Zenodo repository with DOI: 10.5281/zenodo.5726811.

## Abbreviations

ecAGP: α-d-glucose 1-phosphate phosphatase from *Escherichia coli*
EVB: empirical valence bond
Glc1P: α-d-glucose 1-phosphate
HAD: haloalkanoate dehalogenase
LFER: linear free-energy relationship
Man1P: α-d-mannose 1-phosphate
βPGM: β-phosphoglucomutase
PMS: peptide spectrum matches
PEP: phosphoenolpyruvate
