# Supplementary material for: Essential Functional Interplay of the Catalytic Groups in Acid Phosphatase
Source: ACS Catal. 2022 Feb 28;12(6):3357–70. doi: 10.1021/acscatal.1c05656 (PMC8938923; doi:10.1021/acscatal.1c05656)
Supplement: Supplementary file 1 — cs1c05656_si_001.pdf [file cs1c05656_si_001.pdf]

Supporting Information

The Essential Functional Interplay of the Catalytic Groups in Acid Phosphatase

Martin Pfeiffer[1,2], Rory M. Crean[3], Catia Moreira[3], Antonietta Parracino[3], Gustav Oberdorfer[4], Lothar Brecker[5], Friedrich Hammerschmidt[5], Shina Caroline Lynn Kamerlin[3,*] and Bernd Nidetzky[1,2,*]

[1] Institute of Biotechnology and Biochemical Engineering, Graz University of Technology, NAWI Graz, Petersgasse 12, 8010 Graz, Austria.

[2] Austrian Centre of Industrial Biotechnology, Petersgasse 14, 8010 Graz, Austria.

[3] Department of Chemistry–BMC, Uppsala University, BMC Box 576, S-751 23 Uppsala, Sweden.

[4] Institute of Biochemistry, Graz University of Technology, NAWI Graz, Petersgasse 12, 8010 Graz, Austria.

[5] Department of Organic Chemistry, University of Vienna, Vienna, Währingerstraße 38, 1090 Vienna, Austria.

Functional Cooperativity • Enzyme Catalysis • EVB Simulations • Linear Free-Energy Relationship • Nucleophilic Catalysis • Phosphate Transfer

Corresponding Author

* Bernd Nidetzky, email: bernd.nidetzky@tugraz.at (B.N.)

* Shina Caroline Lynn Kamerlin- lynn.kamerlin@kemi.uu.se (S.C.L.K.)

Table of Contents

S1. Supporting materials and methods **S5**
S1.1 Chemicals, enzymes and microbial strains S5
S1.2 Molecular cloning of ecAGP S5
S1.3 Site-directed mutagenesis S7
S1.4 ecAGP S8
S1.5 YidA S10
S1.6 NahK S10
S1.7 Sucrose phosphorylase S11
S1.8 Assays S11
S1.9 Crystallization and data collection S11
S1.10 Structure determination and refinement S12
S1.11 Phosphatase kinetics S12
S1.12 Phosphoryl transfer S13
S1.13 Inhibition of enzymatic hydrolysis of 4-nitrophenyl phosphate by 2-nitrophenol S14
S1.14 Inorganic phosphate-water ^{18}O exchange S14
S1.15 Detection of the phospho-enzyme intermediate by autoradiography S15
S1.16 Reduction of an aspartyl phosphatase intermediate using borohydride S16
S1.17 Detection of homoserine-containing peptides by LC/MS (YidA) S16
S1.18 Detection of homoserine containing peptides by LC/MS (H18D) S17
S1.19 Preparation of the aryl phosphate substrate S19
S1.20 Improved preparation of *P*-chiral phosphoenolpyruvates and derivatization of D-glucose 6-phosphate to give cyclic methyl D-glucose-4,6-phosphates S20
S1.21 [Carboxy-$^{18}O_2$]benzoic acid {[$^{18}O_2$]**3**} S21
S1.22 (*S*)-(–)-2-Chloro-1-phenylethyl [$^{18}O_2$]benzoate {(*S*)-[$^{18}O_2$]**5**} S21
S1.23 (*S*)-(+)-2-Chloro-1-phenyl-[$^{18}O_1$]ethanol {(*S*)-[$^{18}O_1$]**4**} S22
S1.24 (*R*)-(+)-3-Methyl-1-phenylbutane-1,3-[1-$^{18}O_1$]diol {(*R*)-[$^{18}O_1$]**6**} S23
S1.25 (2*S*,6*R*)-(–)- and (2*R*,6*R*)-(–)-4,4-Dimethyl-6-phenyl-1,3,2-[1-$^{18}O_1$]-Dioxaphosphinane-2-[2-^{17}O]oxides {(*R*,*S*P)- and (*R*,*R*P)-[^{17}O,$^{18}O_1$]**7**} S24
S1.26 Ethyl 2-{(2*R*,6*R*)-((4,4-Dimethyl-2-[^{17}O]oxido-6-phenyl-1,3,2-[1-$^{18}O_1$] dioxaphosphinan-2-yl)oxy}-acrylate and Ethyl 2-{(2*S*,6*R*)-((4,4-Dimethyl-2-[^{17}O]oxido-6-phenyl-1,3,2-[1-$^{18}O_1$]dioxaphosphinan-2-yl)oxy}acrylate {(*R*,*R*P)- and (*R*,*S*P)-[^{17}O,$^{18}O_1$]**8**} S25
S1.27 (*R*P)- and (*S*P)-[^{16}O,^{17}O,^{18}O]PEP as sodium salts S26
S1.28 Phosphoryl transfer catalyzed by wild-type ecAGP and H18D using (*R*P)- or (*S*P)-[^{16}O,^{17}O,^{18}O]PEP as the phosphoryl donor substrate S27
S1.29 Conversion of labeled [^{16}O,^{17}O,^{18}O]Glc6P to cyclic methyl D-glucopyranose 4,6-phosphates S29
S1.30 NMR measurements S31
S1.31 NMR spectroscopy-based investigation of the stereochemical course of phosphoryl transfer S34
S1.32 NMR spectroscopic data of methyl α/β-D-glucopyranose-4,6-[^{16}O,^{18}O] phosphates S36
S1.33 System preparation for the empirical valence bond simulations S37
S1.34 Parameterization of the empirical valence bond simulations S38
S1.35 System equilibration for the empirical valence bond simulations S40
S1.36 EVB simulation analysis S41
S2. Supporting figures **S42**
Figure S1. Active-site architecture of phosphatases catalyzing phosphate ester hydrolysis *via* a covalent phospho-enzyme intermediate. S42

Figure S2. Detection of the phospho-enzyme intermediate using autoradiography. S43
Figure S3. Detection of the aspartyl phosphate intermediate in the active site of H18D and YidA using LC-MS^2 analysis. S44
Figure S4. LC–MS-MS spectra of H18D peptides containing the active-site nucleophile, Asp18, reduced to homoserine. S46
Figure S5. Structural localization of aspartate residues of the H18D variant that were identified by LC-MS^2 to have been converted to homoserine. S47
Figure S6. Time course of phosphoryl transfer reaction from Glc1P to Glc catalyzed by ecAGP variants. S48
Figure S7. Time course of phosphoryl transfer reaction from Glc1P to Man catalyzed by WT and H18D. S49
Figure S8. Time course of phosphoryl transfer reaction from Man1P to Glc catalyzed by H18D and WT. S50
Figure S9. In situ ^{31}P NMR monitoring of ^{18}O incorporation into 10 mM inorganic phosphate (P0) from solvent (< 94% $H_2{}^{18}O$). S51
Figure S10. Stereochemical analysis of phosphoryl transfer catalyzed by wild-type and H18D forms of ecAGP. S52
Figure S11. Distribution of oxygen isotopes in axial and equatorial methyl α/β-D-glucopyranose-4,6-[^{16}O,^{17}O,^{18}O]phosphates after cyclisation and methylation. S53
Figure S12. Distribution of oxygen isotopes in axial and equatorial methyl α/β- D-glucopyranose-4,6-[^{16}O,^{17}O,^{18}O]phosphates after cyclization and methylation. S54
Figure S13. Descriptors for the different isomeric chiral species of the axial and equatorial methyl α/β-D-glucopyranose-4,6-phosphates not containing a ^{17}O isotope, but one or two ^{18}O atoms or no ^{18}O at all. S55
Figure S14. NMR spectra of axial and equatorial methyl α/β-D-glucopyranose-4,6-[^{16}O,^{17}O,^{18}O]phosphates in a ~1:1 mixture of DMSO/CD_3OD. S56
Figure S15. Representative structures of the Michaelis complex (MC), transition state (TS) and product state (PS) over the course of the reaction for the WT and H18D variant reactions with Glc1P. S57
Figure S16. Change in the distances of key reacting atoms in non-enzymatic Glc1P phosphoryl transfer reactions, along the EVB energy gap reaction coordinate ($\Delta\varepsilon_{1,2}$). S58
Figure S17. 2-Nitrophenol is not inhibiting the ecAGP catalyzed hydrolysis of 4-nitrophenyl phosphate. S59
Figure S18. SDS PAGE showing the soluble fraction of the *E. coli* cell extract and the corresponding Strep-Tactin-purified enzymes used in this study. S60
Figure S19. ^{1}H and ^{13}C NMR spectra of the bis(cyclohexylammonium) salt of phenyl phosphate. S61
Figure S20. ^{1}H and ^{13}C NMR spectra of the bis(cyclohexylammonium) salt of 2-nitrophenyl phosphate. S62
Figure S21. ^{1}H and ^{13}C NMR spectra of the bis(cyclohexylammonium) salt of 3-nitrophenyl phosphate. S63
Figure S22. ^{1}H and ^{13}C NMR spectra of the bis(cyclohexylammonium) salt of 3-chlorophenyl phosphate. S64
Figure S23. ^{1}H and ^{13}C NMR spectra of the bis(cyclohexylammonium) salt of 4-cyanophenyl phosphate. S65
Figure S24. Valence bond states used to describe the first chemical step catalyzed by WT ecAGP towards the Glc1P substrate. S66
Figure S25. Valence bond states used to describe the first chemical step catalyzed by the H18D enzyme variant of ecAGP towards the Glc1P substrate. S67

Figure S26. The root mean square deviations (RMSD, Å) of all backbone atoms during our EVB equilibration MD simulations. S68
S3. Supporting tables S69
Table S1. Active-site features of phosphatases catalyzing phosphate monoester hydrolysis *via* a covalent phospho-enzyme intermediate. S69
Table S2. Phosphatase activity with the natural substrate Glc1P. S70
Table S3. Peptides identified in YidA incubated with Glc1P that feature conversion of Asp to homoserine. S71
Table S4. Peptide identified in YidA incubated in the absence of Glc1P. . S71
Table S5. Peptides identified in the H18D variant incubated with Glc1P that feature conversion of Asp to homoserine. S72
Table S6. Peptides identified in the H18D variant incubated in the absence of Glc1P (negative control) that feature conversion of Asp to homoserine. S74
Table S7. Relative ratio of occurrence of different axial and equatorial methyl α/β-D-glucopyranose-4,6-[^{16}O,^{18}O]-phosphates. S75
Table S8. Ratio of occurrence of different axial and methyl α/β-D-glucopyranose-4,6-[^{16}O,^{18}O]-phosphates. S76
Table S9. Data collection and refinement statistics (molecular replacement). S77
Table S10. Hydrogen bonds between the active site residues and the substrate (Sub) at the reactant state (RS) and transition state (TS) complex for the WT enzyme. S78
Table S11. Hydrogen bonds between the active site residues and the substrate (Sub) at the reactant state (RS) and transition state (TS) complex for the H18D enzyme. S79
Table S12. Calculated distances (Å) at the Michaelis complexes (MC), transition states (TS) and product states (PS) obtained from our EVB simulations of WT and H18D ecAGP in complex with the substrate Glc1P. S80
Table S13. Electrostatic contributions of individual amino acids ($\Delta\Delta G^{\ddagger}_{elec}$, kcal mol^{-1}) to the calculated activation free energies for the reaction catalyzed by both the WT and H18D enzyme variant. S81
Table S14. Catalytic efficiency of wild-type ecAGP, H18D, D290A and YidA variants. S82
Table S15. Ionized residues and histidine protonation patterns used in EVB simulations. S83
Table S16. EVB mapping parameters used to describe wild-type and H18D catalyzed reactions. S84
S4. Supporting references S85

S1. Supporting materials and methods

S1.1 Chemicals, enzymes and microbial strains

Unless stated otherwise, all chemicals were of highest purity available from Sigma-Aldrich (Vienna, Austria) or Roth (Karlsruhe, Germany). α-D-Glucose 1-phosphate (Glc1P), D-glucose 6-phosphate (Glc6P), 4-nitrophenyl phosphate (4-O_2N-Ph-OPO_3H_2), phenol (PhOH), 4-cyano-phenol (4-NC-PhOH), 3-nitrophenol (3-O_2N-PhOH), 2-nitrophenol (2-O_2N-PhOH), 3-chloro-phenol (3-Cl-PhOH), phosphoenolpyruvate (PEP) and rabbit muscle pyruvate kinase (PK). All nucleotide primers were from Sigma-Aldrich. Restriction enzymes, Phusion polymerase and GenJET® PCR clean up kit were from Thermo Fisher Scientific (Altham, MA, USA). DNA sequencing was done at LGC Genomics (Berlin, Germany). The *E. coli K-12 BW25113* (agp::kanR) knock out strain was from GE-Healthcare (Chicago, Illinois, USA). Anhydrous 1,4-dioxane was refluxed over sodium under argon atmosphere and distilled prior to use. Dry dimethyl sulfoxide (DMSO) and *N,N*-dimethylformamide (DMF), tri-*n*-butylamine, tri-*n*-octylamine, diphenyl chlorophosphate, and potassium *tert*-butoxide were used as supplied. Cation exchange chromatography was performed with Dowex-50W × 8, H^+ (100-200 mesh). ^{18}O-water (98.3% ^{18}O, 0.4% ^{17}O, 1.3% ^{16}O) and $H_2{}^{17}O$ (90.85% ^{17}O, 6.79% ^{16}O, 2.36% ^{18}O) were from NUKEM Isotopes (Alzenau, Germany). $CDCl_3$ (99.8% D), DMSO-d_6 (99.8% D), CD_3OD (99.8% D) and D_2O (99.9% D) were from Euriso-Top GmbH (Saarbrücken, Germany).

S1.2 Molecular cloning of ecAGP

To allow for periplasmic protein expression, the native ecAGP-signal sequence was introduced into the previously described expression vector pMS470_dsbC_ecAGP[1] using circular polymerase extension cloning.[2] Briefly summarized, the signal sequence was amplified from genomic *E. coli* BL21-Gold (DE3) DNA by PCR (30 cycles (98°C, 15 sec/55°C, 20 sec/72°C, 60 sec/72°C, 120 sec) using Phusion polymerase and the signal sequence primer pair (listed

below). The PCR-product contained the signal sequence and the complementary overlaps to the targeted site in the expression vector. The expression vector was linearized and complementary overlaps to the signal sequence were introduced by PCR (30 cycles (98°C, 15 sec/55°C, 20 sec/72°C, 180 sec/72°C, 300 sec) using Phusion polymerase and the pMS470 primer pair (listed below; sequences overlapping with the vector backbone are underlined).

**primer name**	**primer sequences**
SS_fwd	CTTTAACTTTAAGAAGGAGATATAATGAACAAAACGCTAAT
SS_bwd	TGCGGGTGGCTCCAGCTAGCTTGTGCCTGAGCATT
pMS470_fwd	GCTAGCTGGAGCCACC
pMS470_bwd	TATATCTCCTTCTTAAAGTTAAAG
TEV_fwd	GCCGCTCTGGAAGTACAGGTTCTCGCGCCCTTCGATTTTTTCG
TEV_rev	GAGAACCTGTACTTCCAGAGCGGCCAAACCGTACCGGAAGG

Purified PCR products were used in an overlap-extension-PCR (15 cycles (98°C, 15 sec/65°C, 20 sec/72°C, 180 sec/72°C, 300 sec) to prime each other and to create the final expression vector. The desalted PCR product was transformed into *E. coli XL1 Blue* and transformants were selected on LB-agar plates containing 0.1 mg/mL of ampicillin. Correct assembly of the plasmid was verified by sequencing. The construct pMS470-Strep-signal_sequence-ecAGP encodes a *N*-terminally Strep-tagged ecAGP protein. The sequenced vector was transformed into an *agp* knock-out strain *E. coli K-12 BW25113* (agp::kanR) and transformants were selected on LB-agar plates containing 0.1 mg/mL of ampicillin (Amp) and 25 µg/mL kanamycin (Kan).

For crystallization, a TEV-cleavage site was inserted downstream of the *N*-terminal Strep-tag using a modified protocol of the Quick change PCR.[3] pMS470-Strep-signal_sequence-ecAGP was used as the template. In the first step of the two-step PCR protocol, two separate PCR reactions, using either the forward or the backward primer of the TEV-primer pair (TEV,

see above) were performed for each desired mutation. The reaction profile consisted of a pre-heating step at 98°C for 30 sec followed by 6 cycles (98°C, 15 sec/55-65°C, 20 sec/72°C, 180 sec/72°C, 300 sec). In the second PCR reaction, the PCR products (i.e., the linearized vector and the TEV-sequence with overhang to the vector) were combined 1:1 (by volume) and amplification was continued for 12 cycles (98°C, 15 sec/60-70°C, 20 sec/72°C, 180 sec/72°C, 420 sec). The resulting PCR product was subjected to parental template digest by *Dpn*I and transformed into *E. coli* Top 10 cells by electroporation. Single-colony transformants were selected on agar plates containing 0.1 mg/mL ampicillin. Plasmids were amplified in *E. coli* Top 10 cells, isolated and sent for sequencing. Sequenced plasmids harboring the TEV-cleavage site were transformed into electro-competent *E. coli K-12 BW25113* (agp::kanR) and transformants were selected on LB-agar plates containing 0.1 mg/mL of Amp and 25 µg/mL Kan.

S1.3 Site-directed mutagenesis

Site-directed mutagenesis was performed according to the modified Quick change protocol[3] using Phusion polymerase and primers listed below (the mutated triplet codon is underlined).

**primer name**	**primer sequences**
H18A_fwd	CATGATGAGCCGCGCCAACTTACGTGCGCCGCTG
H18A_bwd	CAGCGGCGCACGTAAGTTGGCGCGGCTCATCATG
H18D_fwd	GATGAGCCGCGACAACTTACGTGC
H18D_bwd	GCACGTAAGTTGTCGCGGCTCATC
D290A_fwd	GTTGGGCACGCCTCCAACATTG
D290A_bwd	CAATGTTGGAGGCGTGCCCAAC
H18D_2_fwd	GATGAGCCGCGATAACTTACGTGC
H18D_2_bwd	GCACGTAAGTTATCGCGGCTCATC

pMS470-Strep-signal_sequence-ecAGP was used for the construction of single point variants that were subsequently used for kinetic measurements, except for the H18D (CAC→GAT) and H18D/D290A variants, where pMS470-Strep-signal_sequence-ecAGP_H18D was used. pMS470-Strep-signal_sequence-TEV-ecAGP was used as the template for the construction of variants subsequently used for crystallization. In the first step of the two-step PCR protocol, two separate PCR reactions, using either the forward or the backward primer, were performed for each desired mutation. The reaction profile consisted of a preheating step at 98°C for 30 sec followed by 6 cycles (98°C, 15 sec/55-65°C, 20 sec/72°C, 180 sec/72°C, 300 sec). Resulting PCR products were combined 1:1 (by volume) and the amplification was continued for 12 cycles (98°C, 15 sec/60-70°C, 20 sec/72°C, 180 sec/72°C, 420 sec). The final PCR product was subjected to parental template digest by *Dpn*I and transformed into *E. coli* Top 10 cells by electroporation and single-colony transformants were selected on agar plates containing 0.1 mg/mL ampicillin. Plasmids were amplified in *E. coli* Top 10 cells, isolated and sent for sequencing. Sequenced plasmids harboring the desired mutation were transformed into electrocompetent *E. coli K-12 BW25113* (agp::kanR). Transformants were selected on LB-agar plates containing 0.1 mg/mL of Amp and 25 µg/mL Kan.

S1.4 ecAGP

Two liters of main culture (LB medium, Amp 0.1 mg/mL, Kan 25 µg/mL) were inoculated to an OD_{600} of 0.1 from an overnight culture and incubated at 37°C and 110 rpm. At OD_{600} of about 0.8, expression was induced by 0.1 mM isopropyl β-D-1-thiogalactopyranoside (IPTG). The temperature was reduced to 18°C and expression continued for 20 h. Cells were harvested by centrifugation at 20,000 × *g* at 4°C for 30 min using a Sorvall RC-5B refrigerated super speed centrifuge (Du Pont Instruments, Newtown, CT, USA). The cell pellet was resuspended into 60 mL Strep-tag-wash buffer (100 mM TRIS, 150 mM NaCl, 1 mM EDTA, pH 8.0). Cells were disrupted by sonication and insoluble protein as well as cell debris were removed by

centrifugation at 4°C and 20,000 × *g* for 30 min. The cell lysate (60 mL) was loaded on two 5-mL Strep-Trap-HP columns (GE Healthcare, Little Chalfont, UK), equilibrated in Strep-tag-wash buffer and mounted in series on an ÄKTA prime plus system (GE Healthcare, Little Chalfont, UK). The system was operated with a flow rate of 3 mL/min at 4°C. Protein elution was monitored at 280 nm. To avoid cross contamination, distinct columns were used for the purification of different enzymes (wild-type, WT; variants: H18D, H18A, D290A). Non-specifically bound protein was washed off with 5 column volumes of Strep-tag-wash buffer. The bound enzyme was eluted with Strep-tag-elution buffer (2.5 mM desthiobiotin, 100 mM TRIS, 150 mM NaCl, 1 mM EDTA, pH 8.0). Eluted enzyme was concentrated and rebuffered with Amicon Ultra-15 Centrifugal Filter Units (Merck Millipore) to a total protein concentration of 15 mg/mL in 50 mM HEPES, pH 7.0. Aliquots of 70 µL were stored at -20°C until further use.

For protein crystallization, the *N*-terminal Strep-Tag was cleaved off with TEV-protease (1 OD_{280} TEV/50 OD_{280} ecAGP in 50 mM TRIS, pH 8.0). Addition of reducing agent was avoided to preserve the disulfide bonds present in ecAGP. The resulting cleavage product was applied to 2 × 5 mL Q-HP columns (GE Healthcare, Little Chalfont, UK) equilibrated in 50 mM TRIS (pH 8.0) and mounted on an ÄKTA prime plus system (GE Healthcare). The system was operated with a flow rate of 3 mL/min at 4°C. Protein elution was monitored at 280 nm. Elution was performed by applying a linear gradient (100 mL) of 0 - 100% 50 mM TRIS (pH 8.0) supplemented with 500 mM NaCl. The main peak containing ecAGP was applied to Strep-Tactin purification (as described above). The flow through was collected. It was concentrated to a total volume of 3 mL and applied onto a size exclusion column (Superdex 200, 16/60, 120 mL; GE Healthcare) mounted on an ÄKTA prime plus (GE Healthcare) system, equilibrated in 10 mM HEPES (pH 7.5) containing 500 mM NaCl and operated at a flow of 0.8 mL/min. The main peak containing ecAGP, eluting at around 70 mL (corresponding to a protein dimer as expected), was concentrated and rebuffered with Amicon Ultra-15 Centrifugal Filter Units

(Merck Millipore) to a total protein concentration of 13 mg/mL in 10 mM NaAc (pH 4.5). Aliquots of 70 µL were stored at -70°C until use. Purity of the protein preparations was verified by SDS-PAGE.

The ecAGP activity was assayed as described below in the section phosphatase kinetics. One unit of ecAGP activity is the amount of enzyme releasing 1 µmol of inorganic phosphate from Glc1P per minute under the conditions applied.

S1.5 YidA

The YidA from *E. coli* was used. A *N*-terminally *His*-tagged enzyme preparation was applied. Enzyme expression and purification were performed as described by Wildberger *et al.* .[1]

The YidA activity was assayed as described below in the section phosphatase kinetics except that buffer was supplemented with 1 mM $MgCl_2$. One unit of YidA activity is the amount of enzyme releasing 1 µmol of inorganic phosphate from Glc1P per minute under the conditions applied.

S1.6 NahK

Plasmid vector for expression of His_6-tagged NahK from *Bifidobacterium longum JCM1217* was kindly provided by Prof Motomitsu Kitaoka (Faculty of Agriculture, Niigata University, Niigata, 950-2181, Japan). The enzyme was purified as described previously.[4] NahK activity assay used 37°C and 50 mM HEPES buffer (pH 7.0) containing 2 mM $MgCl_2$. The substrates were ATP (10 mM) and D-mannose (10 mM). The reaction was started with 25 µM enzyme and consumption of ATP was followed by HPLC.[4] One unit of NahK activity is the amount of enzyme producing 1 µmol of α-D-mannose 1-phosphate (Man1P)/min under the conditions used. The enzyme preparation had a specific activity of 0.02 U/mg.

S1.7 Sucrose phosphorylase

The *N*-terminally *Strep*-tagged sucrose phosphorylase (SPase) from *Leuconostoc mesenteroides* was used. *E. coli* BL21-Gold (DE3) cells harboring the pASK-IBA7+ expression vector encoding the SPase were cultivated in 1-L baffled shaken flasks at 37°C and 110 rpm using 250 mL LB medium containing 0.115 mg/mL Amp. At OD_{600} of about 0.8, the temperature was decreased to 22°C and gene expression was induced with 200 μg/L anhydrotetracycline for 20 h. Enzyme purification was performed as described above for ecAGP. SPase activity was determined in 50 mM MES buffer (pH 7.0) at 37°C using 100 mM inorganic phosphate and 100 mM sucrose as the substrates. The reaction was started with 0.3 μM SPase and consumption of phosphate was measured as described under assays. One unit of SPase activity is the amount of enzyme consuming 1 μmol of phosphate per minute under the conditions applied. The enzyme preparations used had a specific activity of about 110 U/mg.

S1.8 Assays

Inorganic phosphate was determined in a colorimetric assay based on the formation of a blue phosphomolybdate complex at 850 nm.[5] Glc1P and Glc6P were assayed enzymatically using Glc6P dehydrogenase and phosphoglucomutase.[6]

Protein concentrations were determined spectrophotometrically at 280 nm using a DeNovix, DS-11+ Spectrophotometer (Thermo Fisher, Altham, MA, USA). The molar extinction coefficients used were calculated by ProtParam[7] and are the following: ecAGP (60850 M^{-1} cm^{-1}, 46674 Da), NahK (27515 M^{-1} cm^{-1}, 39902 Da), SPase(75750M^{-1} cm^{-1}, 56742Da).

S1.9 Crystallization and data collection

Purified TEV-cleaved ecAGP (H18D variant) was crystallized using the sitting-drop vapor diffusion method. Crystallization was performed using protein concentrated to 13 mg/mL. Sitting drops were set up in 96-well 3-drop Swiss CL plates (Molecular dimensions, Newmarket,

Suffolk UK) against JCSG+, Morpheus (Molecular dimensions, Newmarket, Suffolk UK) and Index (Hampton Research, CA, USA) screens. Sitting drops consisted of 250 nL, 250 nL, 500 nL protein solution mixed with 250 nL, 500 nL, 250 nL of precipitant solution respectively. Crystallization drops were equilibrated against a reservoir containing 30 μL of precipitant solution. Crystal hits were detected under various conditions at 293 K (20°C). Crystals formed within the first 3 days of incubation. The best condition was 0.1 M CHES, pH 9.5, 20 % w/v and PEG 800. Crystals were transferred to a cryo-protectant consisting of 10 % glycerol added to the corresponding precipitate solution and flash-frozen in liquid nitrogen. Data were collected in Hamburg (PETRA III, EMBL c/o DESY BEAMLINE P14 (MX2)). The data were integrated with XDS.[8]

S1.10 Structure determination and refinement

The structure of H18D was determined by molecular replacement with *PHASER*[9] using PDB ID: 1NT4 (H18A) as a search model. Model building was performed with COOT[10] and refinement was carried out with *phenix.*refine.[11] The final structures were evaluated using MolProbity.[12] The number of Ramachandran outliers was below 0.2%. Figures of the structural models were prepared using PyMol 1.3. [13]

S1.11 Phosphatase kinetics

The rate constant k_{cat} and the Michaelis constant K_M were determined by measuring the initial rate of phosphate hydrolysis catalyzed by wild-type ecAGP (2 nM), H18D or H18D-2 (110 μM), D290A (2 nM - 5 μM, depending on the substrate used) or H18D/D290A (110 μM) in 50 mM HEPES buffer (pH 7.0). Reactions were incubated at 37°C on a Thermomixer comfort (Eppendorf, Hamburg, Germany) with agitation at 650 rpm. The reactions were started by addition of enzyme. Glc1P*,* Glc6P, and the bis(cyclohexylammonium) salts of phenyl phosphate, 2-nitrophenyl phosphate, 3-nitrophenyl phosphate, 4-nitrophenyl phosphate, 4-cyanophenyl

phosphate and 3-chlorophenyl phosphate were used as substrates in concentrations ranging from 0.01 mM to 10 mM. Incubation times were adjusted to ensure that the overall substrate hydrolysis was below 10 %. Samples withdrawn were quenched by heating (99°C, 5 min) and the free phosphate concentration was measured in supernatants of centrifuged (16000 × *g*, 15 min, 4°C) samples. All reactions were performed in triplicate.

Kinetic parameters were obtained from non-linear least squares fits (SigmaPlot, Systat, Erkrath, Germany) of initial rates of phosphate release to the Michaelis–Menten equation. V is the initial rate, V_{max} is the maximum initial rate, K_M is the Michaelis constant and [S] is the initial substrate concentration (equation 1). The k_{cat} was obtained from the relationship, $k_{cat} = V_{max}/[E]$, where [E] is the molar concentration of active sites. [E] was obtained from the protein concentration using a molecular mass of the ecAGP subunit of 46674 Da.

$$V = V_{max}\,[S] \,/\, (K_M + [S]) \qquad (1)$$

S1.12 Phosphoryl transfer

Reactions were performed at 37°C in 50 mM HEPES buffer (pH 7.0) containing a phosphate donor (Glc1P or Man1P; 2 mM for H18D and H18A; 20 mM for wild-type ecAGP) and an acceptor (Glc or Man; 200 mM). The reaction was started with enzyme (wild-type ecAGP: 1.4 nM, H18A and H18D: 22 µM). Samples were withdrawn at certain times and quenched using heat inactivation (99°C for 5 min). Precipitated protein was removed (centrifugation 13000 × *g*, 10 min, 4°C). Samples were assayed for G1P, G6P and free phosphate content. The concentration of Man1P product was determined indirectly from the phosphate mass balance (equation 2). The Glc1P consumed in the reaction is distributed between free phosphate (hydrolysis), Glc6P (transphosphorylation to the Glc released in hydrolysis) and Man1P.

$$\Delta[\text{Glc1P}] = [\text{Man1P}] + [\text{Glc6P}] + [\text{phosphate}] \qquad (2)$$

S1.13 Inhibition of enzymatic hydrolysis of 4-nitrophenyl phosphate by 2-nitrophenol

Reactions were performed at 37°C in 50 mM HEPES buffer (pH 7.0). The substrate (4-phenyl phosphate) concentration covered a span between 0.05 K_M and 5 K_M (10, 8, 5, 2.5 1, 0.5, 0.3, 0.1, 0 mM). The inhibitor (2-nitrophenol) was added to the reaction at a final concentration of either 3 mM or 6 mM. Reactions were initiated by 0.26 µM wild-type ecAGP. Samples were withdrawn after 20 minutes and inactivated by thermal treatment (99°C, 5 min). Free phosphate concentration was measured in supernatants of centrifuged (16000 × *g*, 15 min, 4°C) samples.

S1.14 Inorganic phosphate-water ^{18}O exchange

Exchange experiments were performed at 25°C in 50 mM HEPES-buffer pH 7.0 with 10 mM potassium phosphate (pH 7.0) prepared in $H_2{}^{18}O$ (98% ^{18}O enrichment) and a total reaction volume of 500 µL. The reaction was initiated by addition of 11 µM wild-type ecAGP or D290A, 44 µM H18A, 220 µM H18D resulting in a final ^{18}O enrichment of 94%. ^{31}P NMR spectra were recorded as described under NMR measurements. Incorporation of ^{18}O into phosphate results in an upfield shift conveniently detected by ^{31}P NMR. Four distinct ^{18}O labeled phosphate species could be detected in ^{31}P NMR spectra: P0 contains no ^{18}O, P1 contains one ^{18}O atom, P2 contains two ^{18}O atoms, P3 contains three ^{18}O atoms, and P4 contains four ^{18}O atoms. The ^{18}O exchange rate *k* was estimated using the Berkley Madonna suite (Berkeley, California, USA) by fitting *k* to the concentrations of P0, P1, P2, P3 and P4 at distinct time points based on the integral of the chemical shifts recorded during ^{31}P NMR measurements. **Eqs. 3-8** describe the kinetic model used whereas the solvent ^{18}O enrichment factor, f, is 0.94. The kinetic model is based on a model published by Eargle *et al.* [14]

$$P0 \underset{1/4k(1\text{-}f)}{\overset{kf}{\rightleftharpoons}} P1 \underset{2/4k(1\text{-}f)}{\overset{3/4kf}{\rightleftharpoons}} P2 \underset{3/4k(1\text{-}f)}{\overset{2/4kf}{\rightleftharpoons}} P3 \underset{k(1\text{-}f)}{\overset{1/4kf}{\rightleftharpoons}} P4 \qquad (3)$$

$$d/dt(P0) = -k\times P0\times f + k\times P1\times f\times(1\text{-}f) \qquad (4)$$

$$d/dt(P1) = k\times P0\times f - k\times 0.75\times P1\times f + k\times P2\times 0.5\times(1\text{-}f) \qquad (5)$$

$$d/dt(P2) = k\times 0.75\times P1\times f - k\times 0.5\times P2\times f + k\times P3\times 0.75\times(1\text{-}f) - k\times P2\times 0.5\times(1\text{-}f) \qquad (6)$$

$$d/dt(P3) = k\times 0.5\times P2\times f - k\times 0.25\times P3\times f + k\times P4\times(1\text{-}f) - k\times P3\times 0.75\times(1\text{-}f) \qquad (7)$$

$$d/dt(P4) = k\times 0.25\times P3\times f - k\times P4\times(1\text{-}f) \qquad (8)$$

The observed exchange rate constant k is converted into the catalytic rate constant k_{cat} by multiplying it by the ratio of moles of substrate per moles of enzyme.

S1.15 Detection of the phospho-enzyme intermediate by autoradiography

Inorganic $^{32}PO_4$ ion (0.25 µCi/µL) was converted to Glc1^{32}P by SPase (5 µM) in reaction mixtures containing 50 mM $^{32}PO_4$, 100 mM sucrose and 50 mM HEPES (pH 7.0) in a total volume of 40 µL. After 5 min of initial incubation at 37°C (enough to reach full conversion of PO_4), Glc1-^{32}P was hydrolyzed by 4.4 µM wild-type ecAGP or 8.8 µM H18A and H18D. The reaction was incubated for 30 sec (wild-type ecAGP) or 15 min (H18A, H18D) and then stopped by addition of 10 µL SDS Gel Loading Dye (4% SDS, 20% glycerol, 10 % 2-mercaptoethanol, 0.004% bromophenol blue and 125 mM TRIS HCl, pH. 6.8). Samples were immediately loaded onto a 12% polyacrylamide SDS gel and resolved by electrophoresis. Gels were dried at 50°C for 1 h and the ^{32}P-enzyme intermediate was visualized by autoradiography using a photosensitive detection plate exposed for 36 h.

Three control experiments were performed using the same reaction setup as described above with minor variations as follows: control 1 did not contain phosphatase, control 2 lacked sucrose phosphorylase and control 3 lacked sucrose. To probe the validity of the method for aspartyl phosphate detection, a reaction was performed with 16 µM YidA.

S1.16 Reduction of an aspartyl phosphatase intermediate using borohydride

The method was adopted from Cronin *et al.*[15]. Purified ecAGP, H18D or YidA (22 µL each) was incubated in 50 mM HEPES (supplemented with 1 mM $MgCl_2$ in reactions with YidA), in a total volume of 50 µL with 20 mM Glc1P for 1 min at 37°C. Five mM of sodium borohydride was added to the reaction from a stock solution of 200 mM sodium borohydride in dimethyl sulfoxide and further incubated for 10 min at room temperature. The reaction was stopped with 10 µL SDS Gel Loading Dye (4% SDS, 20% glycerol, 10% 2-mercaptoethanol, 0.004% bromophenol blue and 0.125 M TRIS HCl, pH. 6.8). Samples were immediately loaded onto a 12% polyacrylamide SDS gel and subjected to electrophoresis. Bands corresponding to the analyzed enzymes were cut out from the gel and stored at -20° C. For each enzyme, a control experiment without Glc1P was performed.

S1.17 Detection of homoserine-containing peptides by LC/MS (YidA)

Protein spots were excised from gels and digested using trypsin, according to the method of Shevchenko *et al.*.[16] Peptide extracts were dissolved in 0.1% formic acid and separated on a nano-HPLC system (LC20A nano, Shimadzu, Vienna). 70 µL samples were injected and concentrated on the loading column (LC Packings C18 Pep- Map™, 5 µm, 100 Å, 300µm inner diameter × 1 mm) for 5 min using 0.1% formic acid as isocratic solvent at a flow rate of 20 µL/min. The column was then switched into the nanoflow circuit, and the sample was loaded on the nanocolumn (LC-Packings C18 PepMap™, 75 µm inner diameter × 150 mm) at a flow rate of 300 nL/min and separated using the following gradient: solvent A: water, 0.3% formic acid, solvent B: acetonitrile/water 80/20 (v/v), 0.3% formic acid; 0 to 5 min: 4% B, after 40 min 55% B, then for 5 min 90% B and 47 minutes re-equilibration at 4% B. The sample was ionized in a Finnigan nano-ESI source equipped with NanoSpray tips (PicoTip™ Emitter, New

Objective, Woburn, MA, USA) and analyzed in a Thermo-Finnigan LTQ linear iontrap mass-spectrometer (Thermo, San Jose, CA, USA). The MS/MS data were analyzed with Spectrum-Mill Rev. 03.03.078 (Agilent, Darmstadt, Germany) software. Acceptance parameters were a protein score of more than 20 and individual peptide scores above 10. "Peak List Files" from RAW-data were created using the "Data Extractor" of the "Agilent - Spectrum Mill" Software (Rev. A. 03.03.078). The following settings were used; *N*-terminus: Hydrogen; *C*-terminus: free acid; Cys modification: carbamido-methylation; sequence tag length > 1; MH+: 350 – 4500 Da; Scan time: 0 – 60 min; Merge Scans +/- 1 sec, +/- 0.1 m/z; Maximum z = 4; Minimum S/N: 3, Find 12C.

The sequence database was user created and contained the protein sequences of YidA and known *E. coli* background proteins. Database searching was performed with the "MS/MS Search" feature of the "Agilent -Spectrum Mill" Software (Rev. 03.03.078) and the following settings were used; enzyme: trypsin; *N*-terminus: hydrogen; *C*-terminus: free acid; allowed missed cleavages: 2; Cys modification: carbamido-methylation; sequence tag length > 3; minimum detected. peaks: 4; minimum matched peak intensity: 50%; precursor mass tolerance: +/- 2.5 Da; product mass tolerance: +/- 0.7 Da; homology search; possible multiple oxidized methionine, acetylated lysine, deaminated asparagine, homoserine instead of aspartate ($COOH \rightarrow CH_2OH$ / −13.979 Da), and *N*-terminal pyroglutamic acid.

As detection of peptides containing the active site residues of H18D failed using the above described procedure an optimized set up was developed for H18D utilizing the more sensitive Orbitrap Velos Pro mass spectrometer.

S1.18 Detection of homoserine containing peptides by LC/MS (H18D)

Excised gel fragments were cut in 1×1 mm piece and washed with H_2O (5 min, 650 rpm), 100 mM NH_4HCO_3 (5 min, 650 rpm), NH_4HCO_3 1:1 acetonitrile mixture (15 min, 650 rpm) and pure acetonitrile (5 min, 650 rpm). The supernatant was removed and gel pieces were dried in

a SpeedVac. Dry samples were reduced using 10 mM dithiothreitol in 100 mM NH_4HCO_3 buffer incubated for 30 min at 56°C. Fully reduced samples were washed with acetonitrile and alkylated by incubation with 55 mM iodoacetamide solution for 20 min at 37°C, and digested with modified trypsin (Promega, Madison, WI, USA) [16], Glu-C (Sigma-Aldrich) or with chymotrypsin (Roche Applied Sciences, Penzberg, Germany) in 50 mM ammonium bicarbonate and 5 mM $CaCl_2$. Peptides were extracted from the gel matrix in 25 mM NH_4HCO_3, acetonitrile and 5% formic acid. For MS analysis, digest extracts were separated by nano-high-performance liquid chromatography (HPLC) using a Dionex Ultimate 3000 equipped with a C18, 5 µm, 100 Å, 5 × 0.3 mm enrichment column and an Acclaim PepMap RSLC nanocolumn (C18, 2 µm, 100 Å, 500 × 0.075 mm) (all from Thermo Fisher Scientific, Waltham, MA, USA). About one half of digested protein was injected and concentrated on the enrichment column for 2 min at a flow rate of 20 µL/min with 0.5% trifluoroacetic acid as an isocratic solvent.

Separation was carried out on the nanocolumn at a flow rate of 300 nL/min using the following gradient, where solvent A was 0.05% trifluoroacetic acid in water and solvent B was 0.05% trifluoroacetic acid in 80% acetonitrile: 0 to 2 min, 4% B; 2 to 70 min, 4 to 28% B; 70 to 94 min, 28 to 50% B; 94 to 96 min, 50 to 95% B; 96 to 116 min, 95% B; 116 to 116.1 min, 95 to 4% B; 116.1 to 140 min, re-equilibration at 4% B. The sample was ionized in the nanospray source equipped with stainless steel emitters (ES528; Thermo Fisher Scientific, Waltham, MA, USA) and analyzed in an Orbitrap Velos Pro mass spectrometer (Thermo Fisher Scientific, Waltham, MA, USA) operated in positive ion mode, applying alternating full-scan MS (m/z 300 to 2,000) in the ICR cell and tandem MS (MS-MS) by CID of the 20 most intense peaks in the ion trap with dynamic exclusion enabled.

The liquid chromatography (LC)–MS-MS data were analyzed by searching a database containing the protein sequences of wild-type ecAGP, H18D and known background proteins including the public Swissprot database *E. coli* with Proteome Discoverer 1.4 (Thermo Fisher

Scientific, Waltham, Massachusetts, USA) and Mascot 2.4.1 (MatrixScience, London, UK). The search criteria were charge, 2+ or 3+; 2 missed cleavages; precursor mass error, 10 ppm; and product mass error, 0.7 Da, with carbamido-methylation on cysteine as static modification, oxidation on methionine, and modification of aspartic acid to homoserine (COOH→ CH_2OH / −13.979 Da) as variable modifications. A maximum false-discovery rate of 0.01 using a decoy database search, an ion score cut-off of 20, and a minimum of 2 identified peptides were chosen as protein identification criteria.

S1.19 Preparation of the aryl phosphate substrates

The method for the synthesis of monoaryl phosphates was adopted from Hall and Williams[18], and was performed for the preparation of cyclohexylammonium salts of 2-nitrophenyl phosphate, 3-nitrophenyl phosphate, 4-nitrophenyl phosphate, 4-cyanophenyl phosphate and 3-chlorophenyl phosphate. Reactions were performed using a Schlenk-line to ensure exclusion of moisture. In brief, 0.1 mol of phenol or substituted phenol was dissolved in pyridine (10 mL) and added (under stirring) to a cooled (on ice) solution of $POCl_3$ (0.1 mol) in pyridine (15 mL). Stirring was continued for 40 min and then the reaction was quenched by pouring onto 20 g of crushed ice. The pH of the solution was adjusted to 9.0 using cyclohexylamine, and the precipitate formed was collected by filtration. The cyclohexylammonium salts were recrystallized twice from 80% ethanol and yields were above 40%. ^1H and ^{13}C NMR spectra of the isolated compounds were recorded. The free phosphate content of products was assayed colorimetrically and found to be below 5%.

S1.20 Improved preparation of *P*-chiral phosphoenolpyruvates and derivitization of D-glucose-6-phosphate to give cyclic methyl D-glucose-4,6-phosphates

The *P*-chiral phosphoenolpyruvates were prepared according to our published procedure[19] from (*R*)-(–)-2-chloro-1-phenylethanol of ee >99%. The other steps were fine tuned to improve yields. The introduction of the ^{17}O-label with $H_2{}^{17}O$ (90.85% ^{17}O, 6.79% ^{16}O, 2.36% ^{18}O compared to 70% ^{17}O, 28% ^{16}O, 2% ^{18}O in the published procedure) into the mixture of phosphoramidites was modified and furnished a mixture of doubly labeled cyclic *H*-phosphonates (combined yield 86%; 11.6% $^{16}O_2{}^{18}O_1$, 85.5% $^{16}O_1{}^{17}O_1{}^{18}O_1$, 2.9% $^{16}O_1{}^{18}O_2$)[19], which were converted to protected cyclic *cis*- and *trans*-enol phosphates (combined yield 70%). 85% of the molecules were chirally labeled at phosphorus atom beside molecules containing one ^{17}O (1%), one ^{18}O (12%) and two ^{18}O (2%). This labelling translated into the labelling of the *P*-chiral PEPs. Removal of the protecting groups delivered (S_P)- and (R_P)-[^{16}O,^{17}O,^{18}O]phosphoenolpyruvate as sodium salt. The procedure for the conversion of the *P*-chiral D-glucopyranose-6-phosphate to cyclic D-glucopyranose-4,6-phosphates developed by Jarvest *et al.* [20]and Seidel *et al.*[21] had to be modified as Glc6P isolated from the enzymatic reaction mixture contained labeled inorganic phosphate (from virtually nothing up to an equimolar amount) and possibly other minor impurities.

The NMR and MS spectrometers, GC-MS and HPLC equipment for the determination of the ee-values, melting point apparatus and polarimeter were the same as previously described.[22]

S1.21 [Carboxy-$^{18}O_2$]benzoic acid {[$^{18}O_2$]3}

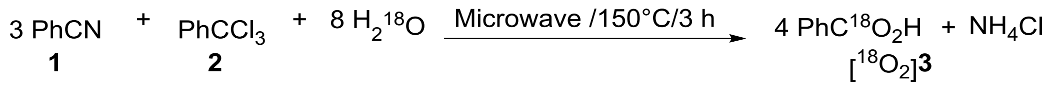

A mixture of $PhCCl_3$ (1.955 g, 10 mmol, 1.05 equiv.), PhCN (2.944 g, 28.57 mmol) and ^{18}O-water (2.0 g, 100 mmol, 98.3% ^{18}O, 0.4% ^{17}O, 1.3% ^{16}O) was heated in the microwave oven at 150°C[23] for 3 h. The labeled benzoic acid of the solid residue was dissolved in CH_2Cl_2. The mixture was dried (Na_2SO_4), filtered and concentrated under reduce pressure. The crystalline residue was heated from 25°C to 90°C/0.8 mbar for drying in the bulb to bulb distillation apparatus to give [carboxy-$^{18}O_2$]benzoic acid {[$^{18}O_2$]**3**} (4.681 g, 98%). The spectroscopic data were identical to those of the literature.[19,23] MS: 2% [$^{16}O_1{}^{18}O_1$]M^-, 98%[$^{18}O_2$]M^-.

S1.22 *(S)*-(–)-2-Chloro-1-phenylethyl [$^{18}O_2$]benzoate {(*S*)-[$^{18}O_2$]5}

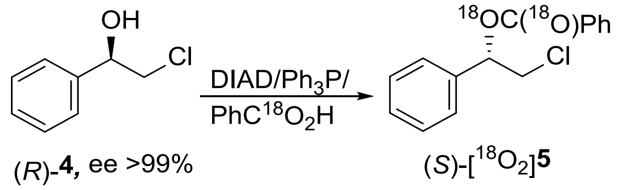

To a stirred mixture of (*R*)-2-chloro-1-phenylethanol [(*R*)-**4**] (7.42 g, 47.38 mmol), Ph_3P (11.496 g, 56.85 mmol, 1.2 equiv.) and [carboxy-$^{18}O_2$]benzoic acid (7.056 g, 56.85 mmol, 1.2 equiv.) in dry toluene (84 mL) at -10°C a solution of diisopropyl azodicarboxylate (DIAD) (11.496 g, 56.85 mmol, 11.19 mL, 1.2 equiv.) in dry toluene (11 mL) was added dropwise. Stirring was continued for 1 h while the temperature of the cooling bath was allowed to rise to 0°C and 1 h at room temperature after removing the cooling bath. Water (1 mL) was added and after 15 min the reaction mixture was concentrated under reduced pressure. The residue was diluted with Et_2O (90 mL) and heptane (90 mL) and cooled at 4 °C for 18 h. The crystals were collected by suction and the filtrate was concentrated under reduced pressure and flash chromatographed (heptane/ethyl acetate/CH_2Cl_2 = 12:1:1, R_f = 0.37) to give ^{18}O-labeled benzoate

(*S*)-[$^{18}O_2$]-**5** (11.940 g, 96%) as nearly colorless oil; $[\alpha]_D^{20}$ = –37.63 (c = 1.6, acetone). The spectroscopic data were identical to those of the literature.[19]

S1.23 (*S*)-(+)-2-Chloro-1-phenyl-[$^{18}O_1$]ethanol {(*S*)-[$^{18}O_1$]4}

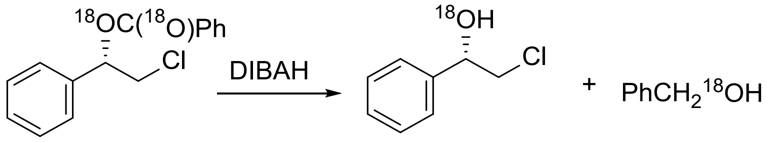

(*S*)-[$^{18}O_2$]**5** (*S*)-[$^{18}O_1$]**4**, ee >99%

Diisobutylaluminum hydride (DIBAH) (113.6 mL, 113.6 mmol, 2.5 equiv., 1 M solution in toluene) was added to a solution of the above benzoate (*S*)-[$^{18}O_2$]**5** (11.940 g, 45.44 mmol) dissolved in dry THF (60 mL) at –78°C under argon. The temperature was allowed to rise to room temperature in the cooling bath in 18 h. The reaction mixture was cooled at 0 °C and water (20 mL) was added very cautiously (note: exothermic reaction and evolution of H_2!) followed by HCl (3 M) to dissolve the formed aluminum hydroxide. After the addition of ethyl acetate (30 mL) the organic layer was separated and the aqueous one was extracted with EtOAc (2 × 30 mL). The combined organic layers were washed with water (2 × 20 mL), dried (Na_2SO_4) and concentrated under reduced pressure. The residue was flash chromatographed (heptane/ethyl acetate/CH_2Cl_2 = 12:1:1, R_f ([^{18}O]benzylalcohol) = 0.13, R_f (^{18}O-alcohol **4**) = 0.28) and bulb-to-bulb distilled to give labeled alcohol (6.521 g, 90%) as colorless liquid; $[\alpha]_D^{20}$ = +44.20 (c = 1.12, acetone); ee >99% by chiral HPLC. The spectroscopic data were in agreement with the literature data.[19]

S1.24 (*R*)-(+)-3-Methyl-1-phenylbutane-1,3-[1-$^{18}O_1$]diol {(*R*)-[$^{18}O_1$]6}

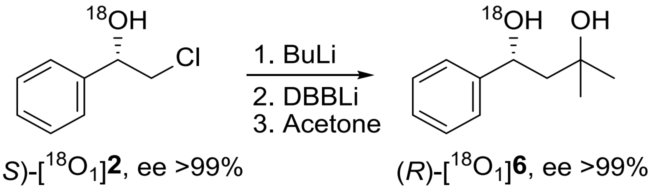

A mixture of 4,4′-di-tert-butylbiphenyl (DBB) (5.328 g, 20 mmol) and Li metal (0.503 g, granular, 0.5% sodium, 4-10 mesh, hammered to a thin foil) in dry THF (50 mL) and a glass-coated stirring bar was sonicated for 5 min at room temperature under an argon atmosphere whereupon a green blue solution started to form. Stirring was continued for 4 h at 0°C and a deep green blue mixture formed.[24–28]

n-BuLi (4 mL, 2.5 M solution in hexanes) was added dropwise to a solution of (*S*)-(+)-2-chloro-1-phenylethanol {(*S*)-[$^{18}O_1$]**4**} (1.264 g, 8 mmol, ee >99%) in dry THF (8 mL) at –78 °C under an argon atmosphere. The solution was cooled to –95°C by adding liquid nitrogen to the dry ice/acetone bath. The above solution of LiDBB was added by using of a syringe. Stirring was continued for 30 min in the cooling bath while the temperature increased to –85°C and color was still blue green. A solution of dry acetone (2 mL, diluted with 2 mL of dry THF) was added drop wise, the temperature was allowed to rise to –78°C and was maintained there. The color changed rapidly to wine red, to brownish and finally to nearly colorless. After 1 h the cooling bath was removed, a mixture of 2 M HCl (25 mL) and a saturated solution of NH_4Cl was added quickly. Ethyl acetate (30 mL) was added. The organic phase was separated, and the aqueous phase was extracted with EtOAc (3 × 30 mL). The combined organic layers were washed with water (2 × 20 mL), dried (Na_2SO_4) and concentrated under reduced pressure. The residue was analyzed by ^1H NMR spectroscopy: Diol:(starting alcohol + 1-phenylethanol) = 82:18 (molar ratio). Flash chromatography (heptane/ethyl acetate 4:1 for elution of DBB, then 3:1, R_f = 0.27 for 1,3-diol) to yield labeled (*R*)-1,3-diol {(*R*)-[$^{18}O_1$]**6**} (1.0 g, 69%); mp.70-71°C (heptane); ee >99% before and after crystallization from heptane; MS: 1%

[$^{16}O_1{}^{18}O_1$]M^++Na, 99% [$^{18}O_2$]M^++Na. The spectroscopic data were in agreement with the literature data.[19]

S1.25 (2*S*,6*R*)-(–)- and (2*R*,6*R*)-(–)-4,4-Dimethyl-6-phenyl-1,3,2-[1-$^{18}O_1$]-dioxaphosphinane-2-[2-^{17}O]oxides {(*R*,*S*P)- and (*R*,*R*P)-[^{17}O,$^{18}O_1$]7}

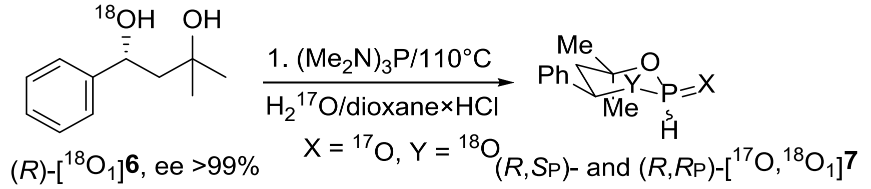

A solution of (*R*)-[^{18}O]diol (*R*)-[$^{18}O_1$]**6** (1.803 g, 10 mmol), $(Me_2N)_3P$ (1.958 g, 12 mmol) and dry CH_3CN (14 mL) was heated at 110°C for 90 min in the microwave oven. After cooling to room temperature, volatiles were removed under reduced pressure (finally at 1 mbar/50 °C). The residue was dissolved in dry THF (12.5 mL) under argon atmosphere and ^{17}O-labeled water (0.28 g, 14.7 mmol, 280 μL, 90.85% ^{17}O, 6.79% ^{16}O, 2.36% ^{18}O; added with a μL syringe) and dioxane × HCl (3.1 mL, about 4 M) were added at 12 °C (bath temperature). After heating the stirred mixture for 10 min at 50°C and cooling at 12°C, hexamethyldisilazane (0.65 mL) was added. The THF was cautiously removed under reduced pressure (1 mbar). Flash chromatography (ethyl acetate, R_f = 0.66) furnished a *cis*/*trans*-mixture of ^{17}O,^{18}O-labeled cyclic *H*-phosphonates (*R*,*S*P)- and (*R*,*R*P)-[^{17}O,$^{18}O_1$]**7** (1.98 g, 86%, ratio of (*R*,*R*P)-[^{17}O,$^{18}O_1$]**7**:/(*R*,*S*P)-[^{17}O,$^{18}O_1$]**7**, = 42:58, by ^{1}H NMR), which crystallized. MS: 11.6% $^{16}O_2{}^{18}O_1$, 85.5% $^{16}O_1{}^{17}O_1{}^{18}O_1$, 2.9% $^{16}O_1{}^{18}O_2$. The spectroscopic data were in agreement with the literature data.[19]

S1.26 Ethyl 2-{(2*R*,6*R*)-((4,4-Dimethyl-2-[^{17}O]oxido-6-phenyl-1,3,2-[1-^{18}O$_1$]dioxaphosphinan-2-yl)oxy}-acrylate and Ethyl 2-{(2*S*,6*R*)-((4,4-Dimethyl-2-[^{17}O]oxido-6-phenyl-1,3,2-[1-^{18}O$_1$]dioxaphosphinan-2-yl)oxy}acrylate {(*R,R*P)- and (*R,S*P)-[^{17}O,^{18}O$_1$]8}

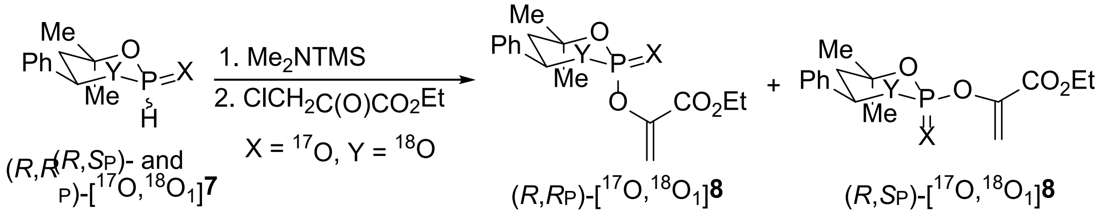

A mixture of *H*-phosphonates (*R*,*S*P)- and (*R*,*R*P)-[^{17}O,^{18}O$_1$]**7** (1.980 g, 8.64 mmol) and *N,N*-dimethyltrimethylsilylamine (3.518 g, 30 mmol, 4.81 mL) was heated with vigorous stirring at 50°C for 1 h. After cooling at room temperature, volatiles were removed at reduced pressure (1 mbar/room temperature, finally at 40°C for 10 min). The residue was dissolved in dry CH_3CN (17 mL) under an argon atmosphere and ethyl 3-chloropyruvate (1.561 g, 10.37 mmol, 1.2 equiv.) dissolved in dry CH_3CN (3.5 mL) was added dropwise at 0°C. After stirring for 5 min at 0°C and 1 h at room temperature, the reaction mixture was concentrated under reduced pressure. The residue ((*R*,*S*P)-[^{17}O,^{18}O$_1$]**8**:(*R*,*R*P)-[^{17}O,^{18}O$_1$]**8** = 1:1, by ^{1}H NMR) was flash-chromatographed (heptane/ethyl acetate 2:1, R_f = 0.3 for *trans*, R_f = 0.12 for *cis*; impure fractions were flash chromatographed again) to give *trans*-enol phosphate: (*R*,*R*P)-[^{17}O,^{18}O$_1$]**8** (0.884 g, 30%) and *cis*-enol phosphate (*R*,*S*P)-[^{17}O,^{18}O$_1$]**8** (1.180 g, 40%) as crystalline compounds. They were crystallized from heptane/1,2-dichloroethane (cooling solution from +30 to –25°C). MS (ESI): 1% ^{17}O$_1$, 12% ^{18}O$_1$, 85% ^{16}O$_1$^{17}O$_1$^{18}O$_1$, 2% ^{18}O$_2$. The spectroscopic data were in agreement with the literature data. [19]

S1.27 (R_P)- and (S_P)-[^{16}O,^{17}O,^{18}O]PEP as sodium salts

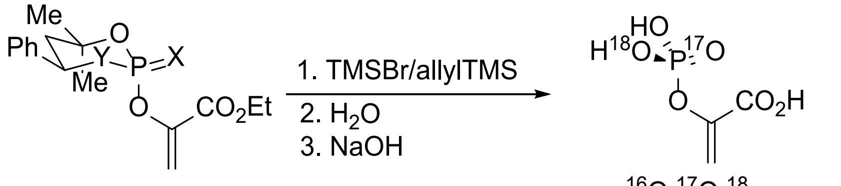

(R,R_P)-[^{17}O,$^{18}O_1$]**8**

(R_P)-[^{16}O,^{17}O,^{18}O)PEP as sodium salt

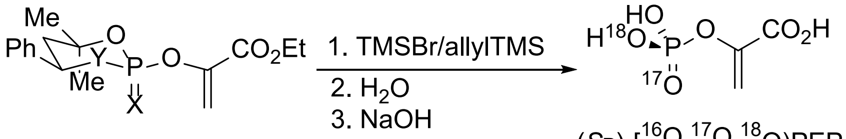

(R,S_P)-[^{17}O,$^{18}O_1$]**8**

(S_P)-[^{16}O,^{17}O,^{18}O)PEP as sodium salt

Allyltrimethylsilane (0.446 g, 3.9 mmol, 3 equiv., 0.62 mL) and bromo(trimethyl)silane (TMSBr) (1.194 g, 7.8 mmol, 6 equiv., 1.03 mL) were added to a solution of the less polar *trans*-enol phosphate (R,R_P)-[^{17}O,$^{18}O_1$]**8** (0.446 g, 1.3 mmol) in 1,2-dichloroethane (5 mL) at room temperature. After stirring for 75 min at room temperature, the reaction mixture was cooled at –15°C and volatiles were removed at reduced pressure (1 mbar/cooling bath was removed). The residue was diluted with 1,2-$C_2H_4Cl_2$ (5 mL) and concentrated at reduced pressure as before (at the end water bath: 25°C - 30°C). NaOH (9.9 mL, 0.5 M) was added to the residue. After stirring vigorously for 5 min, the mixture was extracted with CH_2Cl_2 (3 × 6.5 mL). After the third extraction the aqueous layer was centrifuged (5,600 × g/10 min), rotary evaporated in a weighed round bottomed flask for 2 min without warming to remove last traces of CH_2Cl_2 and left for 3.5 h at room temperature. Then, part of the solution (about 1/3) was removed and applied to Dowex 50W × 8, H^+ (Ø 1.0 cm × 4.5 cm) column and eluted with water until neutral. The eluate was added to the alkaline solution (pH dropped below 6.6). Finally, NaOH (0.25 M) was added to bring the pH to 6.6. The yield of PEP in solution was estimated by ^{31}P NMR spectroscopy, using $H_2NCD_2CH_2PO_3H$ as an internal standard, to be 1.01 mmol (79%) (R_P)-[^{16}O,^{17}O,^{18}O]PEP, as sodium salt. The solution was lyophilized to give salt.

Similarly, the other samples of *trans*-enol phosphate (*R*,*R*P)-[^{17}O,^{18}O$_1$]**8** and *cis*-enol phosphate (*R*,*S*P)-[^{17}O,^{18}O$_1$]**8** (1.3 - 2.88 mmol) were converted to the respective *P*-chiral PEPs (1.14 – 2.1 mmol; yields: 72 - 88%).

S1.28 Phosphoryl transfer catalyzed by wild-type ecAGP and the H18D variant, using (*R*P)- or (*S*P)-[^{16}O,^{17}O,^{18}O]PEP as the phosphoryl donor substrate

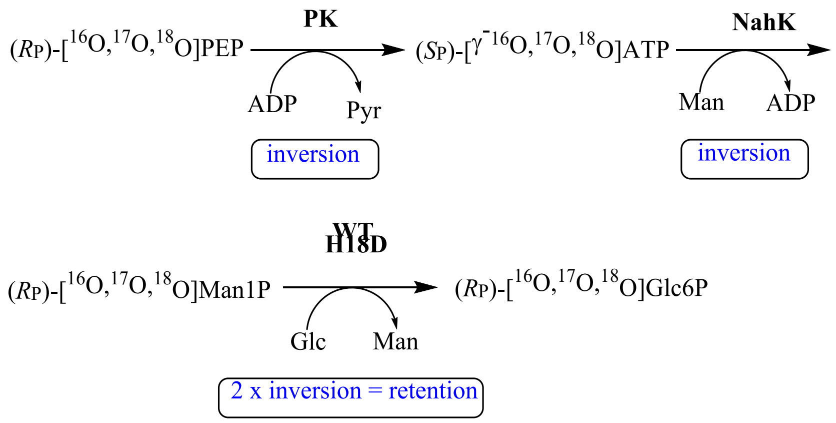

Initially, we examined direct phosphoryl transfer from (*R*P)-[^{16}O,^{17}O,^{18}O]PEP (33 mM) to Glc (800 mM). Reactions were performed at 30°C in in 50 mM HEPES buffer (pH 7.0). Unfortunately, the expected Glc6P was formed in amounts too small to isolate. PEP was not a workable donor substrate for transphosphorylation. We therefore switched to Man1P, which we prepared in two steps, from the *P*-chiral PEP substrates, as show in the scheme above. The reactions of pyruvate kinase (PK) and NahK both proceed with inversion of configuration at phosphorus, leading to retention of *P*-configuration in Man1P.

α-1-Phosphorylation of Man (25 mM) was performed at 30°C in 50 mM HEPES buffer (pH 7.0) with 0.5 mM $MgCl_2$ in a total volume of 30 mL (wild-type ecAGP) or 50 mL (H18D). (*R*P)-[^{16}O,^{17}O,^{18}O]PEP or (*S*P)-[^{16}O,^{17}O,^{18}O]PEP (33 mM) and ADP (1 mM) were used. The reaction was started with PK (0.3 U/mL) and NahK (0.2 U/mL; 25 μM). The reaction was monitored by TLC. One μL of reaction mixture was spotted directly on silica gel plates,

developed using 1-propanol/*n*-butanol/ethanol/H_2O = 2:3:3:2 (by volume) and stained with thymol sugar stain (5 g/L thymol, 5% H_2SO_4 in ethanol). After 24 h, the reaction was stopped by removing the enzyme. Reaction mixtures were centrifuged (20,000 × *g*, 20 min, and 4°C) using Amicon Ultra-15 Centrifugal Filter Units (10 kDa cut-off, Merck Millipore). The reactions gave a yield of approximately 75% (R_P)-Man1P and 50% (S_P)-Man1P, based on TLC analysis.

The reaction mixture was then diluted with 50 mM HEPES (pH 7.0) to a total volume of 100 mL. Glc was added to 800 mM. Transphosphorylation from (R_P)- or (S_P)-Man1P was started by adding enzyme (wild-type ecAGP, 0.07 µM; H18D variant, 22 µM). The formation of α/β-D-glucopyranose-6-[^{16}O,^{17}O,^{18}O]phosphate (in short: [^{16}O,^{17}O,^{18}O]Glc6P)) was monitored over time. Wild-type reactions were complete after 60 min. [^{16}O,^{17}O,^{18}O]Glc6P product concentration from reaction with (R_P)- and (S_P)-Man1P was 5.7 mM and 1.6 mM, respectively. The reasons for this difference were not pursued. Reactions of the H18D variant were complete after about 8 days, with a product concentration of 3.5 mM and 1.0 mM formed from (R_P)- and (S_P)-Man1P, respectively. Enzyme was removed with Amicon Ultra-15 Centrifugal Filter Units (10 kDa cut-off, Merck Millipore; 20,000× *g*, 20 min, 4°C).

The filtered reaction mixture was applied to a FliQ FPLC column (10 mL; 104 × 11.0 mm; Generon, Maidenhead, U.K.) packed with SuperQ-650M (Tosh, Tokyo, Japan) anion-exchange resin. The column was mounted on an ÄKTA prime plus FPLC system (GE Healthcare) and operated at a flow rate of 3 mL/min. The column was pre-equilibrated with 5 mM NH_4HCO_3 in H_2O, pH 8.0. Elution was done with a continuous gradient from 0% to 100% of 100 mM NH_4HCO_3, pH 8.0, in a volume of 120 mL. Fractions were analyzed by TLC. Glc6P-containing fractions were pooled and concentrated to a final volume of 10 mL under reduced pressure, using a rotary evaporator (Heidolph Laborota 4001, Schwabach, Germany) at 40°C.

H_2O (100 mL) was added to the concentrated sample and the concentration process was repeated. Finally, the sample was lyophilized and stored at 4°C until further usage.

Transphosphorylations by wild-type ecAGP and the H18D variant using either (R_P)- or (S_P)-[^{16}O,^{17}O,^{18}O]PEP (1.03 - 2.88 mmol) as the starting material gave [^{16}O,^{17}O,^{18}O]Glc6P (0.1 - 0.34 mmol) in admixture with phosphate (0.04 - 0.1 mmol). The (R_P)- or (S_P)-[^{16}O,^{17}O,^{18}O]Glc6P was further used for analysis of the absolute *P*-configuration.

S1.29 Conversion of labeled [^{16}O,^{17}O,^{18}O]Glc6P to cyclic methyl D-glucopyranose-4,6-phosphates

The procedure for the derivatization of Glc6P in admixture with phosphate was optimized with mixtures of phosphate and commercially available dipotassium Glc6P salt hydrate.[19] The yield of methyl D-glucopyranose-4,6-phosphates was about 10 % as estimated by ^{31}P NMR spectroscopy.

Here, we describe the derivatization of *P*-chiral [^{16}O,^{17}O,^{18}O]Glc6P in admixture with inorganic phosphate, as obtained from conversions of (R_P)-[^{16}O,^{17}O,^{18}O]PEP (1.94 mmol) and the H18D phosphatase, as a representative example.[15,16] The freeze-dried sample of (R_P)-[^{16}O,^{17}O,^{18}O]Glc6P was dissolved in water (8 mL) and gave a slightly brownish colored solution. A sample (100 µL) was withdrawn and mixed with D_2O (350 µL) and a standard solution (50 µL, $H_2NCD_2CH_2PO_3H_2$ in D_2O, 0.1 mmol/mL) in an NMR tube. The sample contained 0.272 mmol of *P*-labeled Glc6P and 0.282 mmol of phosphate in admixture with minor impurities, as estimated by ^{31}P NMR spectroscopy.

The remaining solution (7.9 mL) was applied to a Dowex-50W × 8, H^+ column (Ø 1 cm × 4.5 cm) and eluted with water until neutral. The collected acidic eluate was concentrated under reduced pressure (rotary evaporator, 30°C). Dry dioxane (10 mL) was added to the insoluble residue. The mixture was sonicated for 3 min and again concentrated under reduced pressure. This procedure was repeated twice. Then a solution of tri-*n*-octylamine in dry dioxane (2.33

mL, 0.70 mmol; 0.531 g tri-*n*-octylamine in 5 mL of solution) and MeOH (10 mL, HPLC grade) were added followed by sonication for 3 min whereupon a homogenous solution resulted. The sample was concentration under reduced pressure and the procedure was repeated twice. The resulting solution was divided, and each half was concentrated separately. Both samples were freeze-dried three times with dry dioxane (2 × 5 mL) after sonication for 3 min. The residue of one sample was dissolved in dry dioxane (2 mL) under an argon atmosphere, and solutions of diphenyl chlorophosphate (0.6 mL, 0.43 mmol; 0.970 g, 3.61 mmol in 5 mL solution of dry dioxane) and tri-*n*-butylamine (1.02 mL, 0.43 mmol; 0.394 g, 2.13 mmol in 5 mL solution of dry dioxane) were added at room temperature. After stirring for 10 min, dry DMF (5 mL) was added, followed by a solution of *t*-BuOK (202 mg) in dry DMF (3 mL). The mixture became quickly turbid, and the color changed from nearly colorless to orange-brown.

After 20 min the mixture was centrifuged (5,900 × *g* /10 min/RT). The supernatant was removed and the brownish pellet was suspended in dry DMF (7 mL) under sonication for 3 min and centrifuged again. This procedure was repeated three times. At last, the pellet was dissolved in water (5 mL) and the pH was adjusted to 8.0 ± 0.25 using NaOH. The solution was concentrated in a 25 mL round bottomed flask under reduced pressure. Dry dioxane (5 mL) was added and removed again under reduced pressure. The residue was dried for 20 min at 1 mbar/RT. Dry dioxane (5 mL) was added; the mixture was sonicated for 5 min and freeze-dried (~1 h). This procedure was repeated twice. The last time 18-crown-6 (79 mg) and a stir bar were added and an adaptor with an argon balloon was attached to the flask.

Dry DMSO (1 mL) and methyl iodide (0.15 mL) were added to the residue under argon. The mixture became nearly homogeneous on sonication for 3 min. The flask was covered with an aluminum foil (to protect against light) and the mixture was stirred for 8 h at room temperature. The methylation was complete as proven by ^{31}P NMR spectroscopy (200 μL of sample mixed with 100 μL of dry DMSO and 300 μL of CD_3OD was centrifuged prior to recording

the NMR spectrum). The methyl iodide (MeI) and half (by weight) of the DMSO (800 µL) left was removed at 0.6 mbar and room temperature. The residual DMSO solution was diluted with CH_2Cl_2/MeOH (4:1; 2 mL) whereupon a precipitate was formed.

The brown-red liquid and the precipitate were applied to a column (o.d. 1.3 cm × 40 cm; CH_2Cl_2/MeOH 4:1; elution volume 90 mL, fraction size 11 mL) packed with Merck silica gel 60 (230-400 mesh) in CH_2Cl_2/MeOH (4:1). Brown-red fractions (3-7) were collected concentrated and analyzed by ^{31}P NMR spectroscopy, using CD_3OD in which alone as solvent (the signals were not well resolved especially the one for the cyclic phosphate with the equatorial OMe). The fourth fraction was a mixture of DMSO, product and some impurities. The fifth and sixth (and, in rare cases, the seventh) fraction contained product. Fractions containing the desired cyclic phosphate were pooled and part of the DMSO was removed at 0.7-0.6 mbar/RT. The remaining liquid was assumed to be DMSO for the following calculation. Dry DMSO and CD_3OD were added to get a final volume of 0.55 mL with these components in a ratio of at least 1:1 (by volume; or better 6:4).

S1.30 NMR measurements

Bis(cyclohexylammonium) salts of phenyl phosphates were dissolved in a D_2O (99.90 % D; Euriso-top, Saarbrücken, Germany) in concentrations of ~ 5 mg/mL µg in 600 µL and transferred into 5 mm high precision NMR sample tubes (Promochem, Wesel, Germany). The NMR spectra were recorded on a Bruker AVANCE III 300 spectrometer (Bruker, Rheinstetten, Germany, ^{1}H: 300.36 MHz; ^{13}C: 75.53 MHz) with an autosampler. Chemical shifts δ are referenced to the residual proton and carbon signal of the deuterated solvent ($CDCl_3$: δ = 7.26 ppm (1H), 77.16 ppm (^{13}C); DMSO-d_6: δ = 2.50 ppm (^{1}H), 39.52 ppm (^{13}C); CD_3OD: δ = 3.31 ppm (^{1}H), 49.00 ppm (^{13}C); D_2O: δ = 4.79 ppm (^{1}H)). All measurements have been made at 300 K. Chemical shifts δ are given in ppm (parts per million) and coupling constants J in Hz (Hertz).

Synthetic intermediates (~ 5 mg) isolated during synthesis of methyl α/β-D-glucopyranose-4,6-[^{16}O,^{17}O,^{18}O]-phosphate methyl esters were dissolved in appropriate deuterated solvents in 600 μL and were transferred into 5 mm high precision NMR sample tubes (Promochem). The NMR spectra were recorded on a Bruker AV III-400 AVANCE spectrometer (Bruker) at 400.27 MHz (^{1}H), 100.64 MHz (^{13}C) and 162.03 MHz (^{31}P) and were performed using the Bruker Topspin 3.5 software. All measurements were performed at 298.1 K.

The 1D proton spectra were recorded with a number 32 scans and an acquisition of 64k data points. Fourier transformation led to spectra with a range of 8000 Hz. The 1D ^{13}C spectra were recorded, each with an appropriate number of scans and an acquisition of 64k data points. Lorenz multiplication (1.0 Hz) followed by Fourier transformation led to spectra with a range of 24,000 Hz. The 1D ^{31}P spectra were recorded with an appropriate number of scans and an acquisition of 64k data points. Lorenz multiplication (1.0 Hz) followed by Fourier transformation led to spectra with a range of 16,000 Hz. Chemical shifts were referenced to internal solvent signals for ^{1}H and ^{13}C, respectively. ^{31}P chemical shifts were referenced to external 85% H_3PO_4 (aq) (δ_P 0.00 ppm).

For oxygen isotope exchange investigations, samples were prepared as described in the section "Inorganic Phosphate-Water ^{18}O Exchange" (**Section S.1.14**), and then transferred into 5 mm high precision NMR sample tubes (Promochem). Each sample tube was equipped with a NMR vortex capillary, which was filled with D_2O. Spectra were recorded on a Bruker AV III-600 AVANCE spectrometer (Bruker) equipped with a CryoProbe™ Prodigy (Bruker) and performed using the Bruker Topspin 3.5 software. The 1D ^{31}P spectra were recorded at 242.98 MHz with a number of 64 scans, acquisition of 32k data points, 2.25 sec acquisition time and a relaxation delay of 2.0 sec. Gaussian multiplication (0.075 Hz) and Lorenz multiplication (-0.2 Hz) followed by Fourier transformation led to spectra with a range of 3600 Hz. The ^{31}P NMR chemical shifts were referenced to external 85% H_3PO_4 (aq) (δ_P 0.00 ppm).

The mixtures of mixtures of axial and equatorial methyl α/β-D-glucopyranose-4,6-[^{16}O,^{17}O,^{18}O]phosphates were dissolved in a ~1:1 mixture of DMSO/CD_3OD in concentrations of ~3 µg in 550 µL and transferred into 5 mm high precision NMR sample tubes (Promochem). The spectra were recorded on a Bruker AV III-600 AVANCE spectrometer (Bruker) equipped with a CryoProbe™ Prodigy (Bruker) and were performed using the Bruker Topspin 3.5 software. All measurements were recorded at 298.1 K. The 1D ^{1}H NMR spectra were recorded at 600.27 MHz with a number of 64 scans, acquisition of 64k data points, 2.65 s acquisition time and a relaxation delay of 1.0 s. Fourier transformation led to spectra with a range of 12,000 Hz. ^{1}H NMR chemical shifts were referenced to external acetone (δ_H 2.225 ppm). The 1D ^{31}P spectra were recorded at 242.98 MHz with a number of 5000 scans, acquisition of 5000 data points, 2.50 s acquisition time and a relaxation delay of 2.0 sec. Gaussian multiplication (0.2 Hz) and Lorenz multiplication (-0.4 Hz) followed by Fourier transformation led to spectra with a range of 1000 Hz. The ^{31}P NMR chemical shifts were referenced to external 85% H_3PO_4 (aq) (δ_P 0.00 ppm). The 2D homonuclear ^{1}H/^{1}H-DQF-COSY and ^{1}H/^{1}H-TOCSY (100 ms mixing time) as well as ^{1}H/^{13}C-HSQC and ^{1}H/^{31}P-HSQC spectra were measured with standard Bruker programs.

For each measurement 128 experiments, each with 2048 data points, were recorded with an appropriate number of scans. Linear forward prediction to 256 data points in the f_2 dimension, sinusoidal multiplication in both dimensions and Fourier transformation led to the 2D NMR spectra. Homonuclear 2D spectra were recorded with range of 2400 Hz in both dimensions. ^{1}H/^{13}C-HSQC were recorded have a range of 2400 Hz (^{1}H) and 25,000 Hz (^{13}C) as well as ^{1}H/^{31}P-HSQC were recorded with a range of 2400 Hz (^{1}H) and 1000 Hz (^{31}P), respectively. The ^{13}C NMR chemical shifts were referenced to external acetone (δ_C 30.89 ppm).

S1.31 NMR spectroscopy-based investigation of the stereochemical course of phosphoryl transfer

The products analyzed are axial and equatorial methyl α/β-D-glucopyranose-4,6-[^{16}O,^{18}O]phosphates. Based on the distribution of oxygen isotopes in [^{16}O,^{17}O,^{18}O]PEP (see **Figures S11** and **S12**), this resulted in 32 possible chiral isomeric species of the product with respect to the anomeric centers in the glucose (α or β), the position of the methyl groups in the phosphate esters (axial or equatorial), and the distribution of oxygen isotopes in the phosphate esters. The possible chiral isomeric species and their relative abundance resulting from the distribution of oxygen isotopes in the original (S_P)-[^{16}O,^{17}O,^{18}O]PEP or (R_P)-[^{16}O,^{17}O,^{18}O]PEP are shown in **Figures S11** and **S12**, respectively.

All the isomeric chiral forms, which contain a ^{17}O isotope, lead to large line width at half height of all NMR signals in NMR measurements, which is due to rapid ^{17}O relaxation. Thus, only the 16 possible chiral isomeric species of the axial and equatorial methyl α/β-D-glucopyranose-4,6-[^{16}O,^{18}O]phosphates cause detectable narrow signals.

As shown in **Figure S13**, for these possible chiral isomeric species, descriptors were compiled that take into account the position of the methyl group in phosphate ester (axial or equatorial), as well as the distribution of oxygen isotopes in the phosphate ester. However, the configuration at the anomeric center in the glucose moiety was not considered here. These descriptors were compiled according to the rules of the specification of molecular chirality and used to distinguish these isomeric chiral species.[29]

Figure S14 shows a couple of NMR spectra of the axial and equatorial methyl α/β-D-glucopyranose-4,6-[^{16}O,^{18}O]phosphates derived from the reaction which started with (S_P)-[^{16}O,^{17}O,^{18}O]PEP and where wild-type ecAGP was used. The corresponding NMR spectroscopic data are summarized in **Section S.1.32** "NMR Spectroscopic Data of Methyl α/β-D-Glucopyranose-4,6-[^{16}O,^{18}O]Phosphate".

Figures 2 (main text) and **S10** show the ^{31}P NMR spectra of the products obtained from (R_P)-[^{16}O,^{17}O,^{18}O]PEP and (S_P)-[^{16}O,^{17}O,^{18}O]PEP in reactions with wild-type ecAGP and H18D, respectively. All individual ^{31}P NMR signals can be assigned to the individual chiral species listed in **Figures S11** to **S13**.[19,20] The separate integration of these signals allows a determination of the relative proportions of the different chiral isomers in each reaction. This is possible due to the measurement conditions (see **Section S.1.30** "NMR Measurements"), as well as the comparable relaxation times of the different axial and equatorial methyl α/β-D-glucopyranose-4,6-[^{16}O,^{18}O]phosphates.

In **Table S7**, for the reactions starting from (S_P)-[^{16}O,^{17}O,^{18}O]PEP, the theoretical relative ratios of the isomeric forms resulting from **Figure S10** are summarized, assuming that each (bio)synthetic step takes place with 100% inversion of the configuration at the phosphorus atom in the phosphate. This was compared to the relative integrals of the ^{31}P NMR signals, which result from the spectra shown in **Figure S10**, both for the reactions with wild-type ecAGP and H18D, respectively. The same was done in **Table S8** for the conversions starting from (R_P)-[^{16}O,^{17}O,^{18}O]PEP (**Figure 2**, main text).

For all chiral species not based on the [^{16}O,^{17}O,^{18}O] oxygen isotope distribution, relative abundances of the possible isotopic distributions result, which correspond very well to those of the calculated ones (**Tables S7** and **S8**). Deviations are mostly in the range of +/- 10 % and may have different experimental causes. There was probably a low level of Glc6P with ^{16}O from the preparations of the enzymes. Furthermore, however, slight racemization or hydrolysis may also be possible in the various enzymatic and chemical reaction steps. Likewise, it should be noted that in quantification *via* integrals of NMR spectra errors in the single-digit percentage range are generally not uncommon. In principle, however, the isotope distributions detected *via* the NMR measurements agree very well with those of the theoretical analysis.

S1.32 NMR spectroscopic data of methyl α/β-D-glucopyranose-4,6-[^{16}O,^{18}O]phosphates

Axial methyl α-D-glucopyranose 4,6-[^{16}O,^{18}O]phosphate - ^{1}H NMR (600 MHz, DMSO/CD_3OD) δ 5.17 (d, *J* = 3.9 Hz, 1H, H-1); 4.42 (m_c, 1H, H-6a); 4.21 (m_c, 1H, H-6b). 3.99 (m_c, 1H, H-4); 3.86 (m_c, 1H, H-5); 3.85 (m_c, 1H, H-3); 3.47 (dd, *J* = 9.3, 3.9 Hz, 1H, H-2); ^{13}C NMR (150 MHz, DMSO/CD_3OD) δ (determined from ^{1}H/^{13}C HSQC) 93.5 (CH, C-1); 81.5 (CH, C-4); 72.5 (CH, C-2); 70.5 (CH, C-3); 69.5 (CH_2, C-6). 66.0 (CH, C-5); ^{31}P NMR (242 MHz, DMSO-d_6/CD_3OD) δ -5.16 ((*R*$_P$)-P[4×^{16}O]); -5.16 (3.7 Hz shift) ((*S*$_P$)-P[3×^{16}O, P-^{18}O-CH_3]); -5.16 (10.0 Hz shift) ((*R*$_P$)-P[3×^{16}O, P=^{18}O]); -5.16 (13.8 Hz shift) ((*R*$_P$)-P[2×^{16}O, P=^{18}O, P-^{18}O-CH_3]).

Equatorial methyl α-D-glucopyranose 4,6-[^{16}O,^{18}O]phosphate - ^{1}H NMR (600 MHz, DMSO/CD_3OD) δ 5.18 (d, *J* = 3.9 Hz, 1H, H-1); 4.48 (m_c, 1H, H-6a); 4.30 (m_c, 1H, H-6b). 4.14 (dd, *J* = 9.0, 9.0 Hz, 1H, H-4); 3.87 (m_c, 1H, H-3); 3.86 (m_c, 1H, H-5); 3.48 (dd, *J* = 9.3, 3.9 Hz, 1H, H-2); ^{13}C NMR (150 MHz, DMSO-d_6/CD_3OD) δ (determined from ^{1}H/^{13}C HSQC) 93.5 (CH, C-1); 80.5 (CH, C-4); 72.5 (CH, C-2); 71.0 (CH, C-3); 68.5 (CH_2, C-6). 66.0 (CH, C-5); ^{31}P NMR (242 MHz, DMSO/CD_3OD) δ -3.23 ((*S*$_P$)-P[4×^{16}O]); -3.23 (4.6 Hz shift) ((*R*$_P$)-P[3×^{16}O, P-^{18}O-CH_3]); -3.23 (10.5 Hz shift) ((*S*$_P$)-P[3×^{16}O, P=^{18}O]); -3.23 (15.3 Hz shift) ((*S*$_P$)-P[2×^{16}O, P=^{18}O, P-^{18}O-CH_3]).

Axial methyl β-D-glucopyranose 4,6-[^{16}O,^{18}O]phosphate - ^{1}H NMR (600 MHz, DMSO/CD_3OD) δ 4.68 (d, *J* = 7.8 Hz, 1H, H-1); 4.47 (m_c, 1H, H-6a); 4.20 (m_c, 1H, H-6b). 3.98 (m_c, 1H, H-4); 3.84 (m_c, 1H, H-5); 3.66 (dd, *J* = 9.2, 8.8 Hz, 1H, H-3); 3.22 (dd, *J* = 9.2, 7.8 Hz, 1H, H-2); ^{13}C NMR (150 MHz, DMSO/CD_3OD) δ (determined from ^{1}H/^{13}C HSQC) 98.0 (CH, C-1); 81.5 (CH, C-4); 75.0 (CH, C-2); 73.5 (CH, C-3); 69.0 (CH_2, C-6). 66.0 (CH, C-5); ^{31}P NMR (242 MHz, DMSO-d_6/CD_3OD) δ -5.10 ((*R*$_P$)-P[4×^{16}O]); -5.10 (3.7 Hz shift)

((S_P)-P[3×^{16}O, P-^{18}O-CH_3]); -5.10 (10.0 Hz shift) ((R_P)-P[3×^{16}O, P=^{18}O]); -5.10 (13.8 Hz shift) ((R_P)-P[2×^{16}O, P=^{18}O, P-^{18}O-CH_3]).

Equatorial methyl β-D-glucopyranose 4,6-[^{16}O,^{18}O]phosphate - ^{1}H NMR (600 MHz, DMSO-d_6/CD_3OD) δ 4.69 (d, J = 7.9 Hz, 1H, H-1); 4.55 (m_c, 1H, H-6a); 4.31 (m_c, 1H, H-6b). 4.13 (dd, J = 9.0, 9.0 Hz, 1H, H-4); 3.95 (m_c, 1H, H-5); 3.66 (m_c, 1H, H-3); 3.24 (dd, J = 9.0, 7.9 Hz, 1H, H-2); ^{13}C NMR (150 MHz, DMSO/CD_3OD) δ (determined from ^{1}H/^{13}C HSQC) 98.0 (CH, C-1); 80.5 (CH, C-4); 75.5 (CH, C-2); 73.0 (CH, C-3); 68.0 (CH_2, C-6). 66.0 (CH, C-5); ^{31}P NMR (242 MHz, DMSO/CD_3OD) δ -3.30 ((S_P)-P[4×^{16}O]); -3.30 (4.6 Hz shift) ((R_P)-P[3×^{16}O, P-^{18}O-CH_3]);-3.30 (10.5 Hz shift) ((S_P)-P[3×^{16}O, P-^{18}O-CH_3]); -3.30 (15.3 Hz shift) ((S_P)-P[2×^{16}O, P=^{18}O, P=^{18}O]).

S1.33 System preparation for the empirical valence bond simulations

Empirical valence bond (EVB) simulations were performed using the *Q6* simulation package[30], modeling the first reaction step (**Figure 1** of the main text) of the reactions catalyzed by both the wild-type (WT) and H18D variants of ecAGP. Two structures for ecAGP are available, one of H18D generated in this study (PDB ID: 6RMR) and one from H18A in complex with beta-Glc1P published by Lee et al (PDB ID: 1NT4.)[31]. These structures have a different secondary structure and conformation in the region relatively close to the active site for residues 23-30 (whilst 1NT4 is largely in α-helical conformation over these residues, 6RMR has a loop like structure). Pymol was used for *in silico* substitution of residue 18 (WT to His, H18D to Asp). Appropriate rotamers that did not introduce clashes and made chemical sense were chosen for both structures. Despite structural differences, EVB tests on WT ecAGP with both structures available showed no significant difference in the activation energies calculated, although the 1NT4 structure was notably more stable over the course of the equilibration simulations (as determined by the $C_α$ RMSD, data not shown). We therefore used this (1NT4)

structure for the full-scale production simulations of both enzymes. Docking of the substrate α-D-glucose 1-phosphate (Glc1P) was performed using Autodock vina v.1.1.2[32,33] (docking prepared with MGLTools v.1.5.6.) with all parameters set to their default values, except for the exhaustiveness which was set to 256. Docking poses were filtered by matching reacting distances and their Autodock energy score, from which one low energy and chemically sensible pose was identified.

Any required Asn or Gln side chain flips or histidine tautomerization state changes were performed using MolProbity[12]. Both systems were solvated in a 23 Å radius droplet of TIP3P[34] water molecules described using the surface constrained all-atom solvent (SCAAS)[35] model, with the water droplet centered on the C_β atom of residue 18 (His in the WT enzyme and Asp in the H18D variant). Consistent with this model, residues within 85% of the 23 Å sphere are fully mobile, whilst those in the remaining 15% are restrained to their crystallographic positions using 10 kcal mol^{-1} $Å^{-2}$ harmonic restraints. Any residue outside the sphere is restrained with harmonic positional restraints, but with a force constant of 200 kcal mol^{-1} $Å^{-2}$ (essentially fully immobilized). Restrained residues (those in both the 15% region and outside the droplet) were simulated in their uncharged forms in order to avoid introducing system instabilities by including charged residues outside the explicit water droplet. Residues with the 85% region of the sphere were simulated with ionization states as predicted from pK_a estimates with PROPKA v. 3.1.[36] Residues simulated in their nonstandard protonated states and His tautomerization state assignments are provided in **Table S15**.

S1.34 Parameterization of the empirical valence bond simulations

EVB simulations require a well-defined reference state in order to calibrate the EVB parameters: the off-diagonal coupling element (H_{ij}), and the gas-phase shift (α), for further details, see *e.g.* refs. [37,38] Central to the EVB philosophy is a well-defined reference state (for example the wild-type enzyme against a series of variants or the non-enzymatic reaction in solution of

vacuum). The EVB parabola are fit to reproduce activation and reaction free energies obtained preferably experimentally, or from high level quantum chemical calculations in the absence of experimental data, and the resulting parameter set is then used unchanged to describe all systems of interest. This is feasible due to the phase-independence of the EVB off-diagonal coupling element, as demonstrated in *e.g.* refs. [39,40].

In the present study, we used as our reference state model systems describing the non-enzymatic versions of the reactions catalyzed by WT ecAGP and the H18D variants. This involved modeling nucleophilic attack of either 4-ethyl-1H-imidazole (model for WT ecAGP) or priopionate (model for the H18D variant) on Glc1P, with a second propionate molecule acting as a general acid (as a model for protonated D290 in the enzyme active site). The corresponding valence bond states used to describe this reaction are shown in **Figures S24** and **S25**. We calibrated our EVB parameters to reproduce $\Delta G^{\ddagger}$ values of 26.5 and 34.2 kcal mol^{-1} and ΔG_0 values of 2.6 and 10.3 kcal mol^{-1} for the WT and H18D non-enzymatic reactions, respectively, extrapolating from data presented in refs. [41–43]. This calibration resulted in the EVB parameters provided in **Table S16**.

The OPLS-AA force field was used in consistency with our prior studies. Partial charges for the substrate, product and phosphorylated residues were calculated using the standard restrained electrostatic potential (RESP)[44] fitting procedure with Gaussian 16 Rev. A.03[45] at HF/6-31G(d) level of theory. All other force field parameters for the substrates and phosphorylated residues were obtained using the "ffld_server", from Schrödinger's MacroModel suite.[46] These parameters were converted into *Q6* compatible format using Qtools v0.5.10 (DOI: 10.5281/zenodo.842003). The Morse bonding and non-bonding parameters for the H18D reaction mechanism were taken from our previous EVB study[43] of the analogous conversion of β-glucose-1-phosphate to β-glucose-6-phosphate by the enzyme β-phosphoglucomutase, in which the same reacting atoms and reaction mechanism used are the same as for the H18D

variant. For the WT reaction mechanism, the Morse bonding and non-bonding parameters were taken from our previous EVB study[47] of the enzymes serum paraoxonase 1 (PON1) and diisopropyl fluorophosphatase (DFPase), where both enzymes contain an aspartic residue that had been suggested to be involved in direct nucleophilic attack on the organophosphate substrate, leading to a covalent phosphoenzyme intermediate.[48–50] All EVB parameters, input files and starting structures necessary to reproduce our work are available at the Zenodo repository with DOI: 10.5281/zenodo.5726811.

S1.35 System equilibration for the empirical valence bond simulations

EVB simulations in this work were performed using the *Q6* simulation package,[30] with the OPLS-AA force field. A 10 Å cut-off was set for all non-bonded interactions, except for those involved in the chemical reactions, for which the cut-off was set to 99 Å (effectively no cut-off). Long-range electrostatic interactions were described using the local reaction field (LRF) approach,[51] with temperature regulated by the Berendsen thermostat.[52] A sequence of short equilibration and heating simulations were first used to gradually remove any steric clashes and bad contacts in the system, prior to equilibration at 300 K. At 300 K, all restraints on the mobile region of the protein were removed, and weak (0.5 kcal mol^{-1} $Å^{-2}$) harmonic positional restraints were kept on the reacting atoms. Following this, we performed a further 20 ns of equilibration to fully equilibrate the system (backbone RMSDs over the equilibration period for both systems are provided in **Figure S26**).

All equilibrations were performed at the approximate EVB transition state ($\lambda = 0.5$), as this allows for EVB trajectories in the reactant and product directions to be propagated simultaneously. We performed 30 replicas of both equilibration and production EVB simulations for both systems in order to generate reproducible results. The final structure from each individual equilibration run was used as the starting point for a subsequent EVB production simulation. Production EVB simulations were performed using the valence bond states depicted in **Figures**

S24 and **S25**. The weak harmonic positional restraints of 0.5 kcal mol^{-1} $Å^{-2}$ placed on the reactive atoms during the equilibration were retained for the production EVB simulations as well.

S1.36 EVB simulation analysis

The root mean square deviations (RMSD) of all backbone atoms and hydrogen bonding interactions over the course of our simulations were determined with CPPTRAJ,[53] alongside the clustering (DBSCAN algorithm). A hydrogen bond was defined as being present if the donor-acceptor distance was ≤3.5 Å and if the donor-hydrogen-acceptor angle was 180 ± 45°. All other analyses were performed using Qtools v0.5.10 (DOI: 10.5281/zenodo.842003). The centroids of the top ranked cluster at each EVB λ value were used as the representative structures as seen in **Figures 4** and **S15**.

S2. Supporting figures

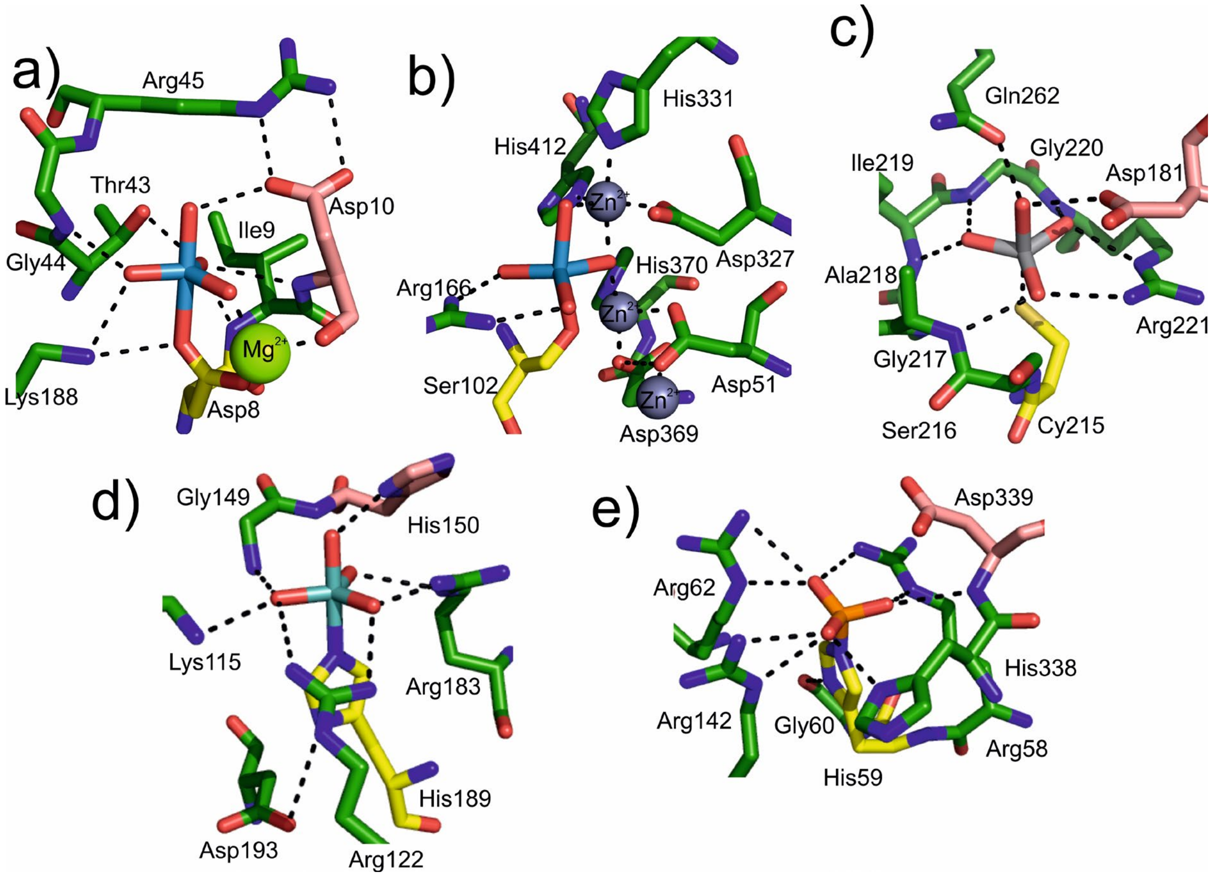

Figure S1. Active-site architecture of phosphatases catalyzing phosphate ester hydrolysis *via* a covalent phospho-enzyme intermediate. (**a**) HAD-phosphatase (PDB ID: 2RB5)[54]. (**b**) Alkaline phosphatase (PDB ID: 5C66)[55]. (**c**) Protein tyrosine phosphate (PDB ID: 1Z12)[56]. (**d**) Histidine-dependent phosphatase (PDB ID: 1EOI)[57] and (**e**) Histidine acid phosphatases/phytases (PDB ID: 1QWO)[58]. Color coding: nucleophile, yellow; acid-base residue, light red; residues involved in phosphate binding and transition state stabilization, green.

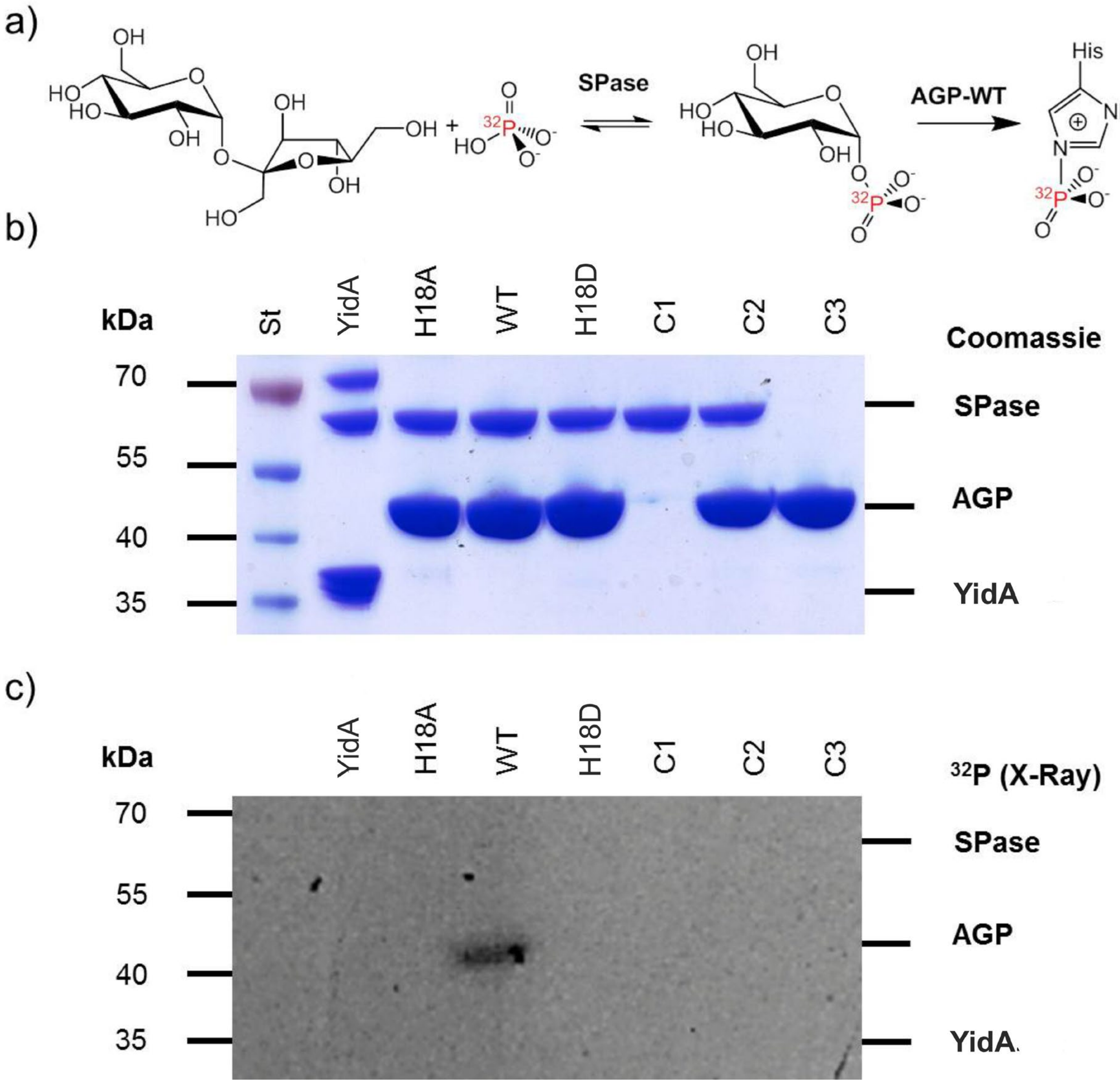

Figure S2. Detection of the phospho-enzyme intermediate using autoradiography. (**a**) Radioactively labeled Glc1-^{32}P was synthesized enzymatically and was offered as substrate for YidA (16 µM), H18A (8.8 µM), H18D (8.8 µM) and wild-type AGP (8.8 µM). (**b**) Quenched reactions were analysed by SDS-PAGE. (**c**) Phosphorylated enzyme was detected by autoradiography. In control C1, no phosphatase was applied. In control C2, no sucrose phosphorylase (SPase) was used and so no Glc1-^{32}P was present. Control C3 lacked the sucrose substrate for formation of Glc1-^{32}P. Detection of the phospho-enzyme intermediate was not possible for YidA and H18D. The aspartyl phosphate may have been too labile for detection. The half-life of carboxyl phosphates is relatively small ($t_{1/2}$ = ~20h).[59]

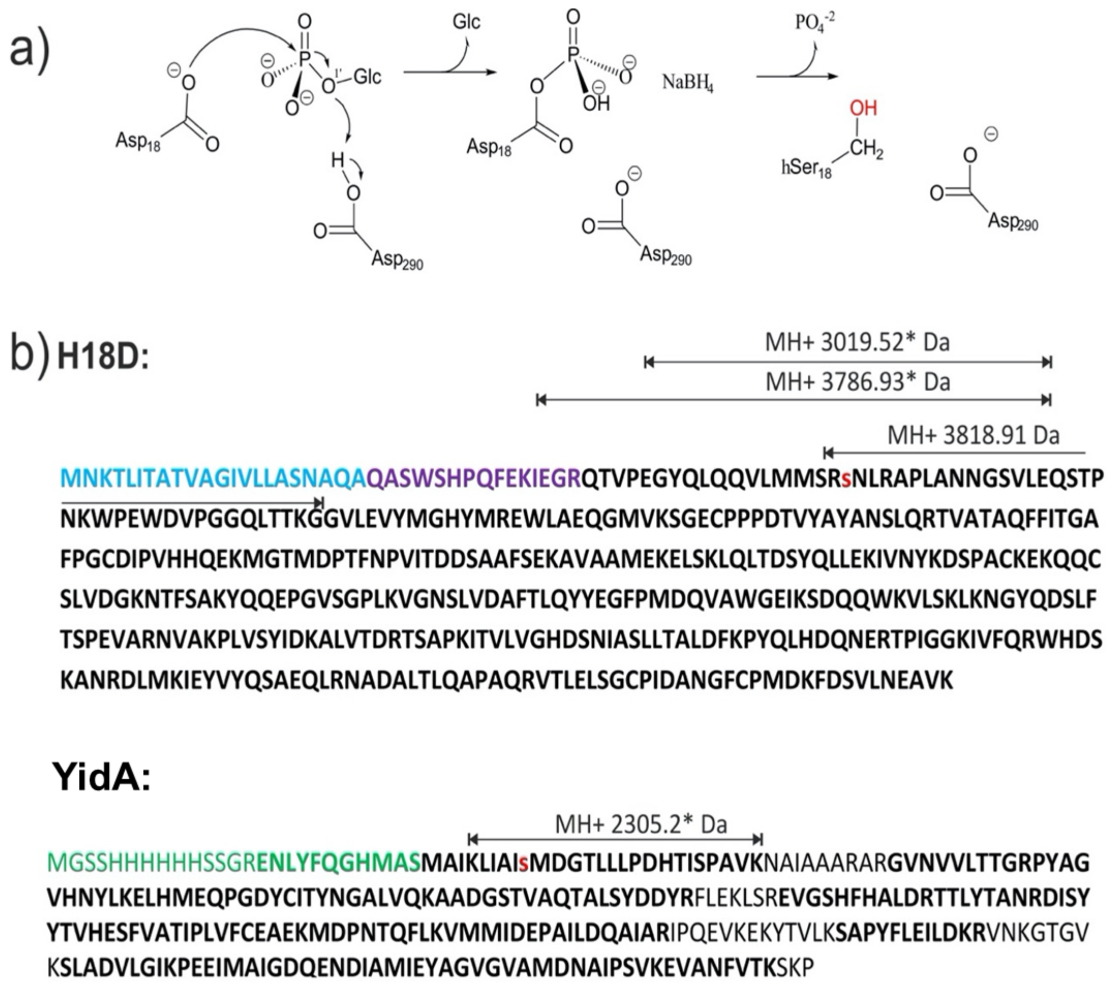

Figure S3. Detection of the aspartyl phosphate intermediate in the active site of H18D and YidA using LC-MS^2 analysis. (**a**) The carboxyl phosphate intermediate is converted to homoserine by NaBH4. (**b**) Detected peptides covering the protein sequence are highlighted in bold letters. Peptides containing homoserine indicative of the aspartyl phosphate intermediate are marked by a double arrow with the corresponding mass indicated. *Mass considers methionine oxidation.

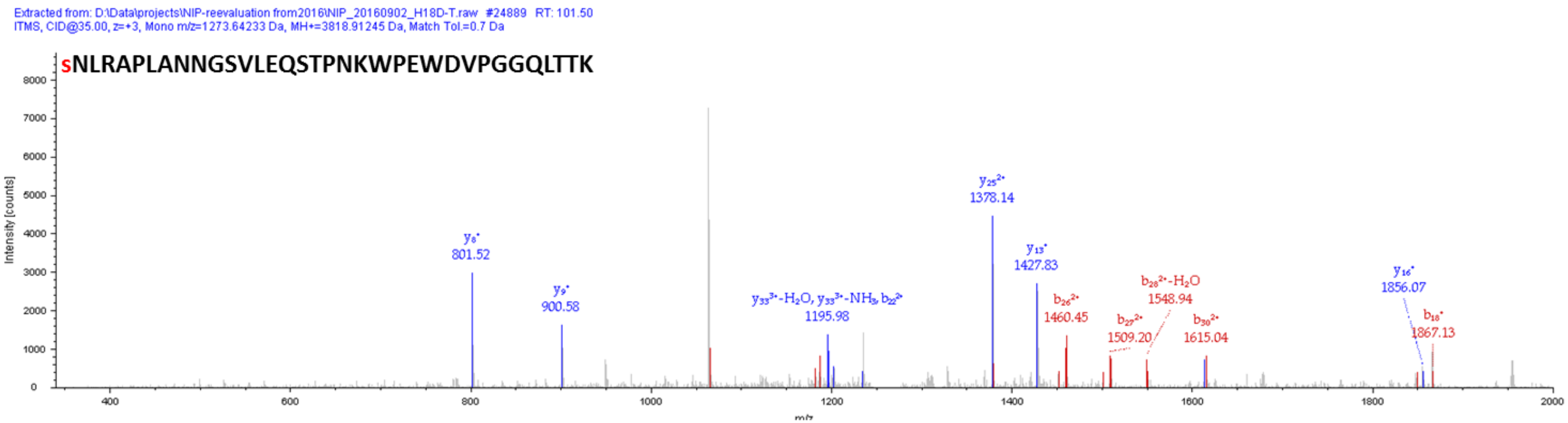
Extracted from: D:\Data\projects\NIP-reevaluation from2016\NIP_20160902_H18D-T.raw #24889 RT: 101.50
ITMS, CID@35.00, z=+3, Mono m/z=1273.64233 Da, MH+=3818.91245 Da, Match Tol.=0.7 Da
sNLRAPLANNGSVLEQSTPNKWPEWDVPGGQLTTK
Intensity [counts]
801.52
900.58
1195.98
1378.14
1427.83
1460.45
1509.20
1548.94
1615.04
1856.07
1867.13
m/z

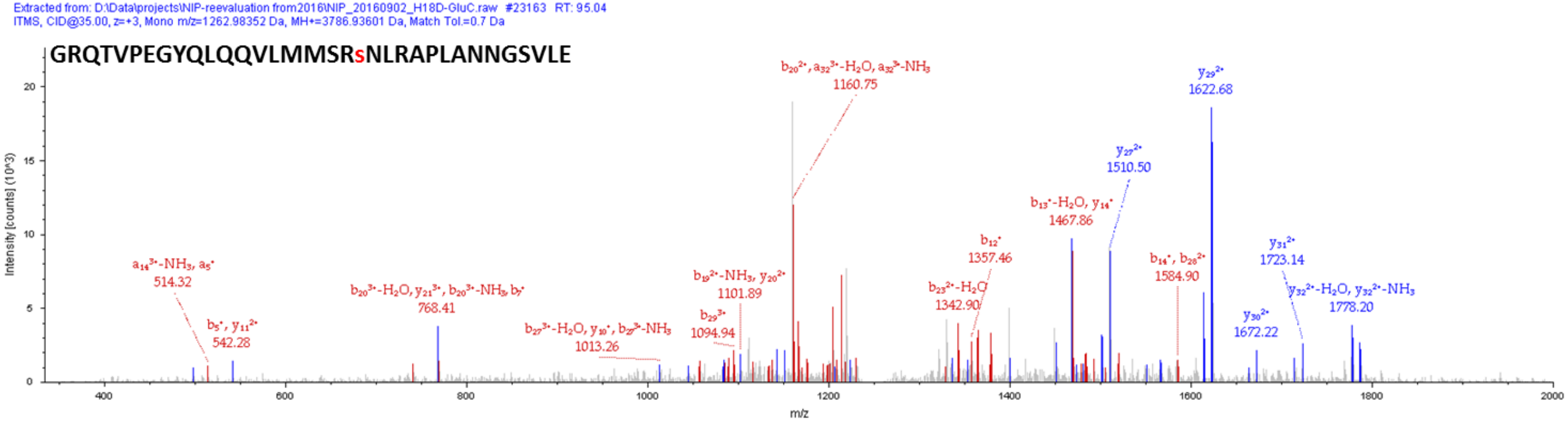
Extracted from: D:\Data\projects\NIP-reevaluation from2016\NIP_20160902_H18D-GluC.raw #23163 RT: 95.04
ITMS, CID@35.00, z=+3, Mono m/z=1262.98352 Da, MH+=3786.93601 Da, Match Tol.=0.7 Da
GRQTVPEGYQLQQVLMMSRsNLRAPLANNGSVLE
Intensity [counts] (10^3)
514.32
542.28
768.41
1013.26
1094.94
1101.89
1160.75
1342.90
1357.46
1467.86
1510.50
1584.90
1622.68
1672.22
1723.14
1778.20
m/z

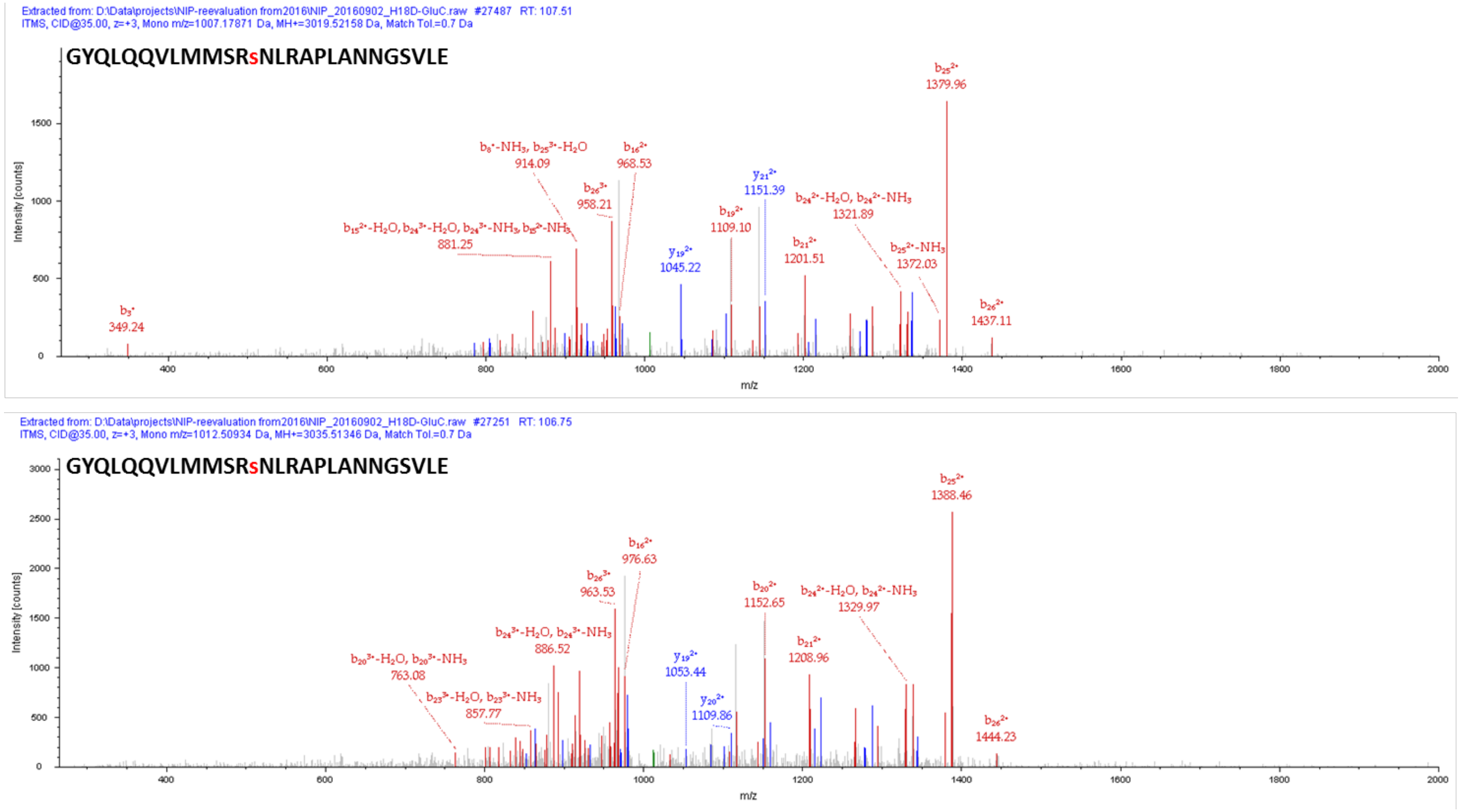

Figure S4. LC–MS-MS spectra of H18D peptides containing the active-site nucleophile, Asp18, reduced to homoserine.

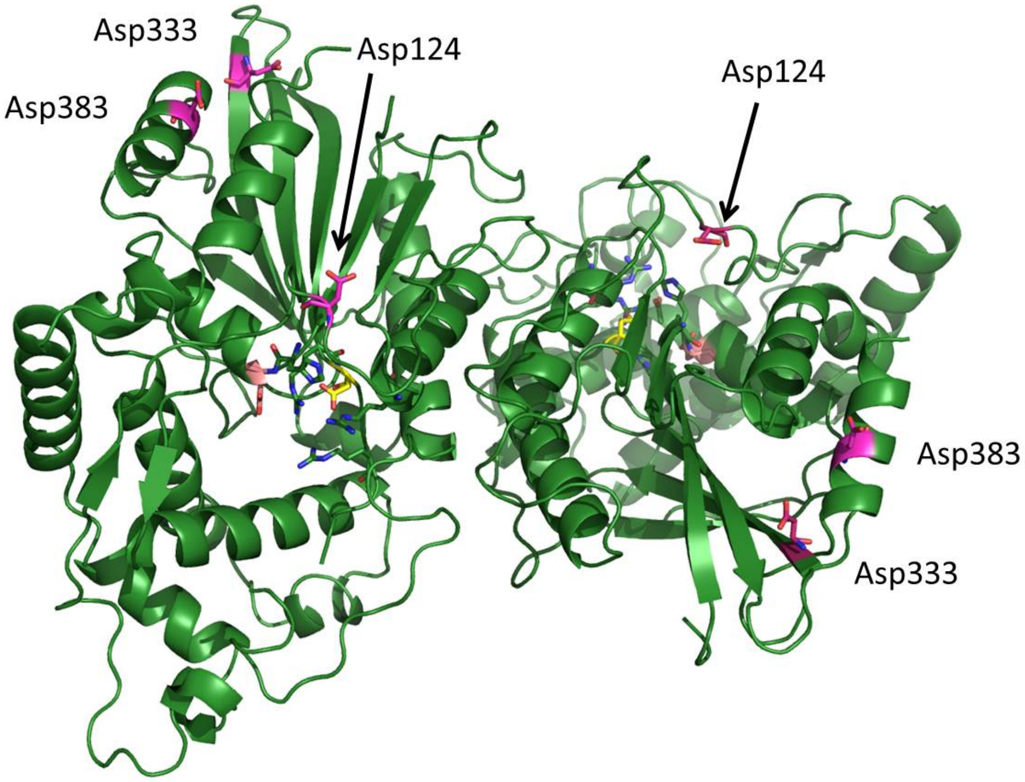

Figure S5. Structural localization of the aspartate residues of the H18D variant that were identified by LC-MS^2 to have been converted to homoserine. The active-site nucleophile Asp18 (yellow) is modified specifically, *i.e.* only when the enzyme is incubated in the presence of Glc1P. Aspartate residues that are converted unselectively (i.e. in the presence and absence of Glc1P) are located on the protein surface. They are highlighted in pink. Note: the acid-base catalytic residue (Asp290) in the active site is never modified. This emphasizes the selectivity of conversion of Asp18 to homoserine and supports the formation of aspartyl phosphate at position 18.

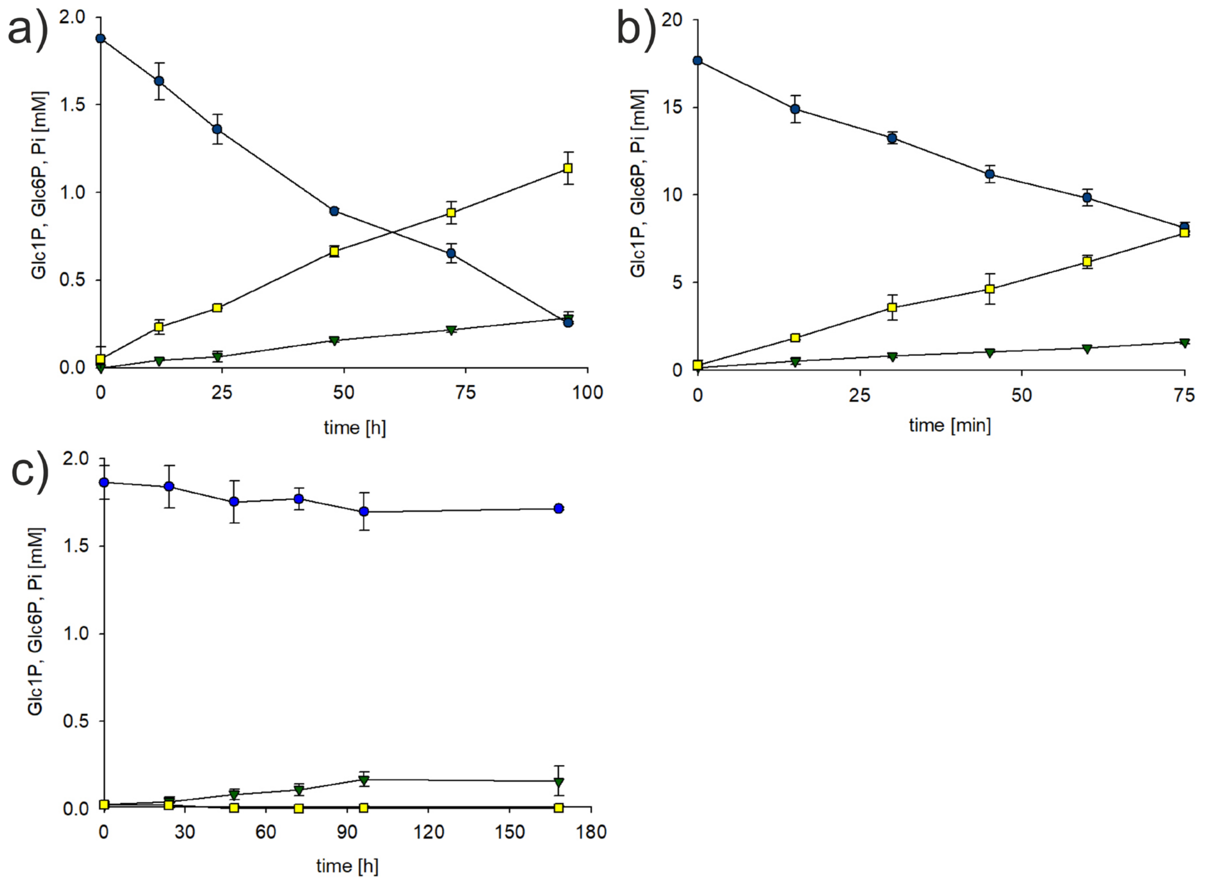

Figure S6. Time course of phosphoryl transfer reaction from Glc1P to Glc catalyzed by ecAGP variants. (**a**) 44 µM H18D variant, (**b**) 0.02 µM wild-type ecAGP and (**c**) 44 µM H18A variant from 2 mM or 20 mM Glc1P (blue) to 200 mM Glc producing Glc6P (yellow) and phosphate (green).

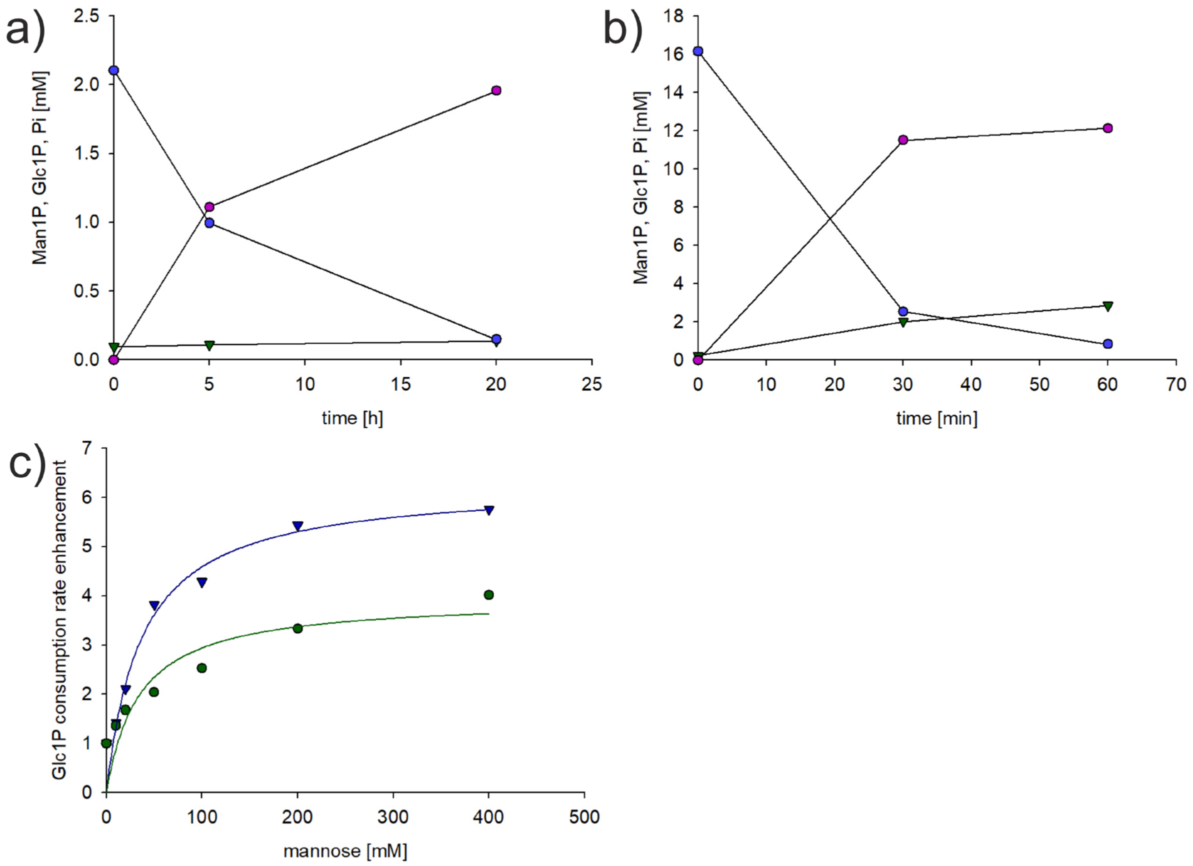

Figure S7. Time course of phosphoryl transfer reaction from Glc1P to Man catalyzed by WT and H18D. (**a**) 44 µM H18D variant and (**b**) 0.02 µM wild-type ecAGP from 2.0 or 16 mM Glc1P (blue) to 200 mM Man producing Man1P (purple) and phosphate (P_i, green). (**c**) Rate enhancement in Glc1P consumption dependent on the Man concentration (0 mM - 400 mM). Data are shown (H18D, blue; wild-type enzyme, green) with symbols and lines show hyperbolic fits. The fits give half-saturation constants of 36 ± 11 mM (H18D) and 34 ± 4 mM (wild-type enzyme).

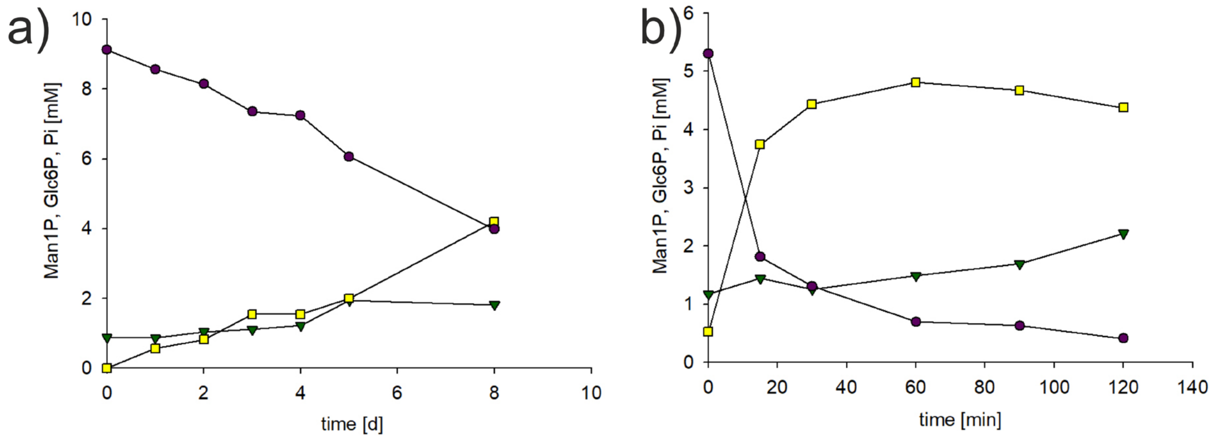

Figure S8. Time course of phosphoryl transfer reaction from Man1P to Glc catalyzed by H18D and WT. (**a**) 44 μM H18D and (**b**) 0.07 μM wild-type ecAGP from 10 mM Man1P (purple) to 200 mM Glc, producing Glc6P (yellow). The hydrolysis product phosphate (P_i, green) is also shown.

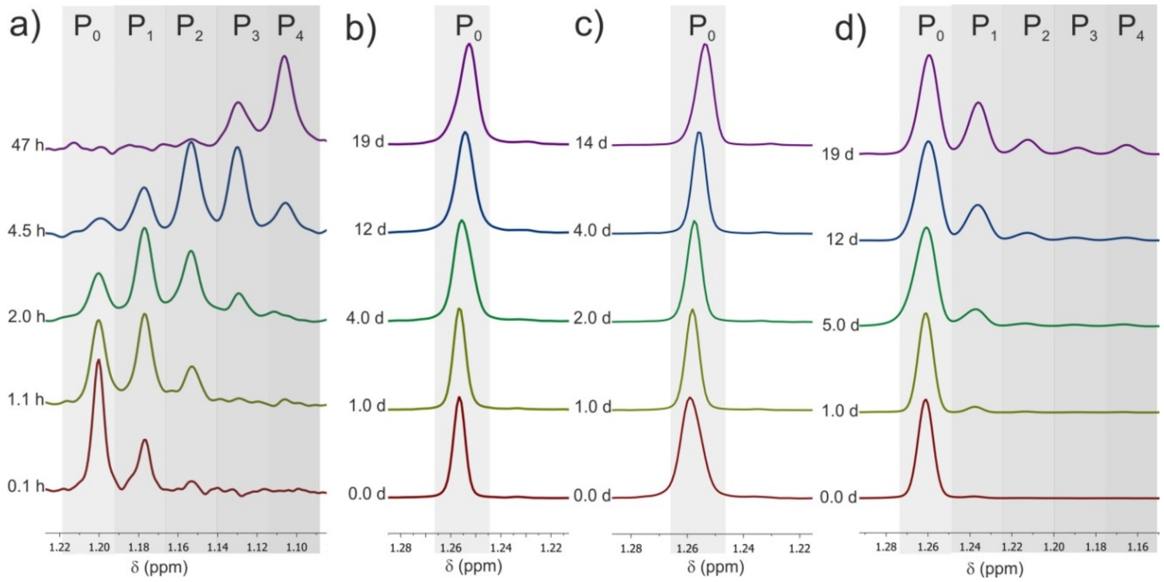

Figure S9. *In situ* ^{31}P NMR monitoring of ^{18}O incorporation into 10 mM inorganic phosphate (P0) from solvent (< 94% $H_2{}^{18}O$). Reaction are catalyzed by (**a**) 11 μM wild-type ecAGP, (**b**) 11 μM D290A, (**c**) 44 μM H18A and (**d**) 220 μM H18D. P_0 contains no ^{18}O atom, P_1 one ^{18}O atom, P_2 two ^{18}O atoms, P_3 three ^{18}O atoms, and P_4 four ^{18}O atoms.

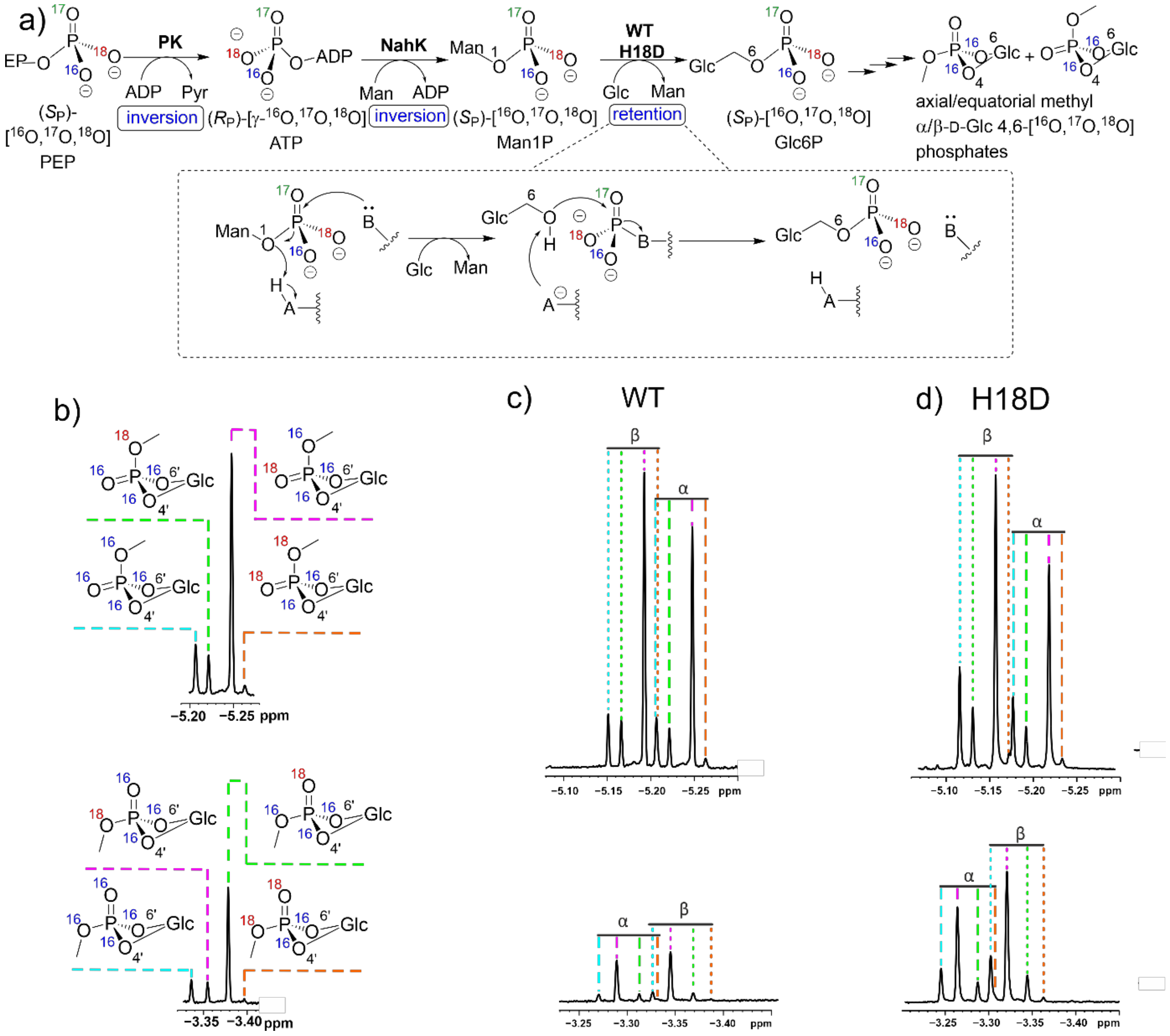

Figure S10. Stereochemical analysis of phosphoryl transfer catalyzed by wild-type and H18D forms of ecAGP. (**a**) The analytical principle, starting from (S_P)-[^{16}O,^{17}O,^{18}O]PEP to give the correspondingly chiral Man1P donor for enzymatic transphosphorylation to glucose, is shown. The Glc6P thus produced is derivatized via cyclization and methylation and is then analyzed by ^{31}P NMR. (**b-d**) The four detectable ^{31}P NMR signals (not including ^{17}O labeled products, as ^{17}O relaxation causes large line widths at half height for all NMR signals) of α/β-D-glucopyranose-4,6-[^{16}O,^{17}O,^{18}O]phosphate methyl esters are shown. The also observed, doubly labeled (2x ^{16}O or 2x ^{18}O) cyclic phospho-methyl-esters originate from a small amount of non-uniform isotope labeling during [^{16}O,^{17}O,^{18}O]PEP synthesis and phosphoryl transfer from ATP (**Figures S11** and **S12**). The signals of α- and β-configured product are indicated with dashed and dotted lines, respectively. Further details are provided in the **Section S.1.32** ("NMR Spectroscopy-based investigation of the stereochemical course of phosphoryl transfer"), as well as in **Figures S11** to **S14** and **Tables S7** and **S8**.

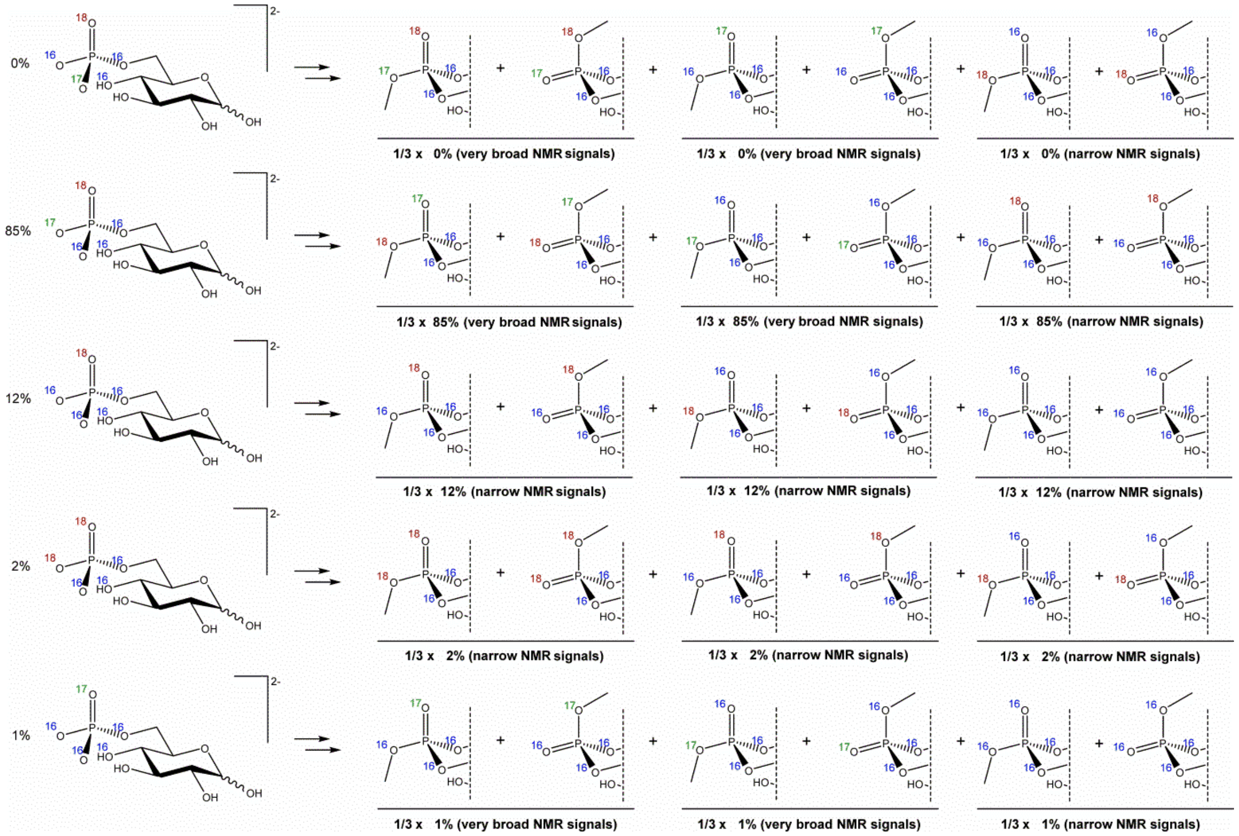

Figure S11. Distribution of oxygen isotopes in axial and equatorial methyl α/β-D-glucopyranose-4,6-[^{16}O,^{17}O,^{18}O]phosphates after cyclisation and methylation. Relative ratio of occurrence of different axial and equatorial methyl α/β-D-glucopyranose-4,6-[^{16}O,^{18}O]phosphates is shown for (S_P)-[^{16}O,^{17}O,^{18}O]PEP as starting material. The observed doubly labeled (2×^{16}O or 2×^{18}O) methyl α/β-D-glucopyranose-4,6-phosphates originate from minor amounts of non-uniform isotope labeling during [^{16}O,^{17}O,^{18}O]PEP synthesis and phosphoryltransfer from ATP.

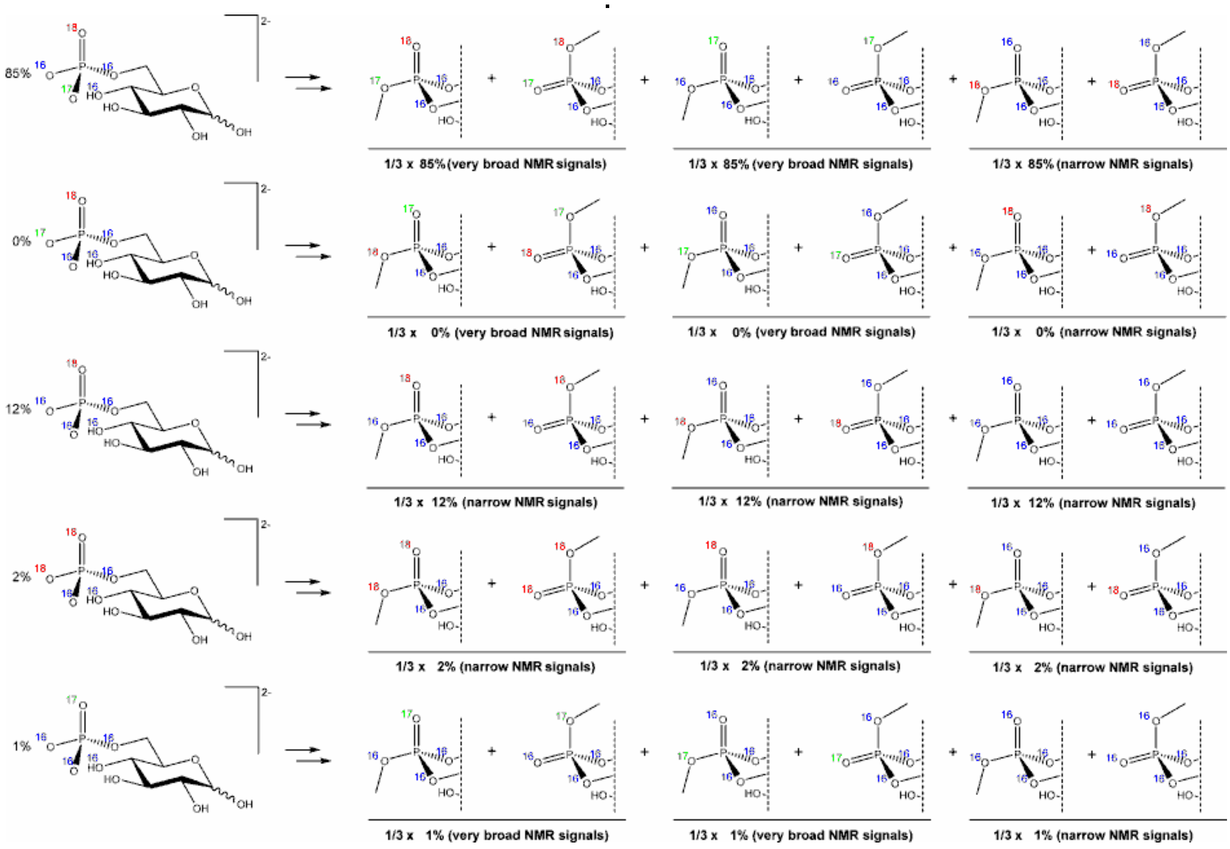

Figure S12. Distribution of oxygen isotopes in axial and equatorial methyl α/β-D-glucopyranose-4,6-[^{16}O,^{17}O,^{18}O]phosphates after cyclization and methylation. Relative ratio of occurrence of different axial and equatorial methyl α/β-D-glucopyranose-4,6-[^{16}O,^{18}O]phosphates is shown for (R_P)-[^{16}O,^{17}O,^{18}O]PEP as starting material. The observed doubly labeled (2×^{16}O or 2×^{18}O) methyl α/β-D-glucopyranose-4,6-phosphates originate from minor amounts of non-uniform isotope labeling during [^{16}O,^{17}O,^{18}O]PEP synthesis and phosphoryltransfer from ATP.

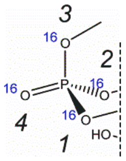

axial methyl D-glucopyranose-4,6-{(S_{P})-[4×^{16}O]}phosphate

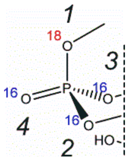

axial methyl D-glucopyranose-4,6-{(S_{P})-[3×^{16}O, P-^{18}O-CH_3]}phosphate

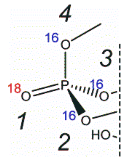

axial methyl D-glucopyranose-4,6-{(R_{P})-[3×^{16}O, P=^{18}O]}phosphate

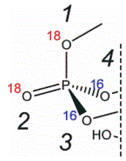

axial methyl D-glucopyranose-4,6-{(S_{P})-[2×^{16}O, P=^{18}O, P-^{18}O-CH_3]}phosphate

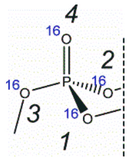

equatorial methyl D-glucopyranose-4,6-{(R_{P})-[4×^{16}O]}-phosphate

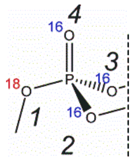

equatorial methyl D-glucopyranose-4,6-{(R_{P})-[3×^{16}O, P-^{18}O-CH_3]}phosphate

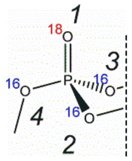

equatorial methyl D-glucopyranose-4,6-{(S_{P})-[3×^{16}O, P=^{18}O]}phosphate

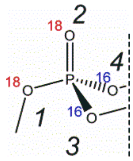

equatorial methyl D-glucopyranose-4,6-{(R_{P})-[2×^{16}O, P=^{18}O, P-^{18}O-CH_3]}phosphate

Figure S13. Descriptors for the different isomeric chiral species of the axial and equatorial methyl α/β-D-glucopyranose-4,6-phosphates containing a ^{17}O isotope, but one or two ^{18}O atoms or no ^{18}O at all. The structures are shown without indicating the α and β anomeric configurations. The italicized numbers illustrate the order of priority of the substituents on the phosphorus atom. These as well as the resulting descriptors were determined according to the rules of the specification of molecular chirality[29] [see chapter 2.2 (b) in reference].

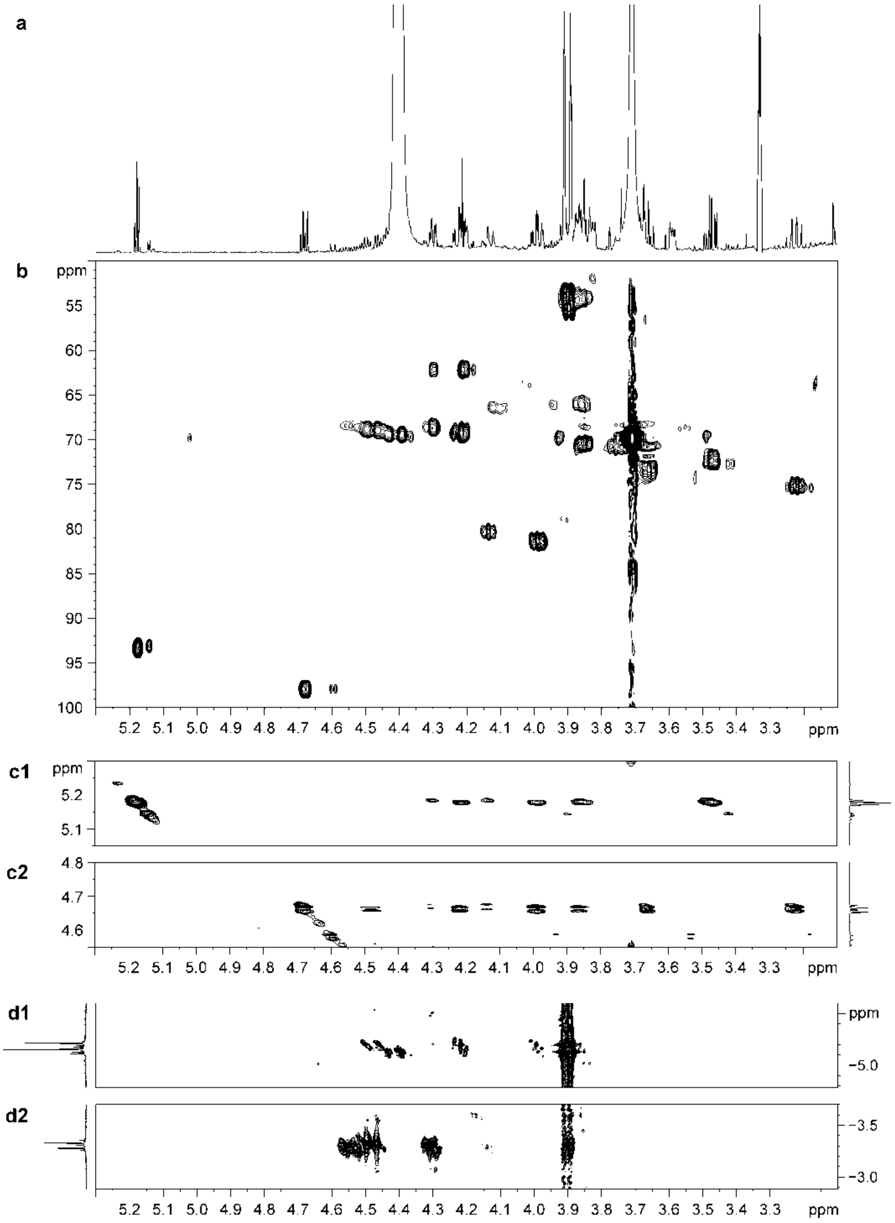

Figure S14. NMR spectra of axial and equatorial methyl α/β-D-glucopyranose-4,6-[^{16}O,^{17}O,^{18}O]phosphates in a ~1:1 mixture of DMSO/CD_3OD. Detectable signals belong to the isomers not containing ^{17}O, as rapid ^{17}O relaxation caused large line width at half height for all NMR signals of ^{17}O containing molecules. Shown are (**a**) the ^{1}H NMR spectrum, (**b**) ^{1}H/^{13}C-HSQC, (**c**) traces from ^{1}H/^{1}H-TOCSY (100 ms mixing time) with couplings of the anomeric protons (H-1) in axial and equatorial methyl α-D-glucopyranose-4,6-[^{16}O,^{17}O,^{18}O]phosphates (c1) and in axial and equatorial methyl β-D-glucopyranose-4,6-[^{16}O,^{17}O,^{18}O]phosphates (c2) as well as traces from ^{1}H/^{31}P-HSQC with ^{1}H-^{31}P couplings in the axial species of methyl α/β-D-glucopyranose-4,6-[^{16}O,^{17}O,^{18}O]phosphates (**d1**) and equatorial species of methyl α/β-D-glucopyranose-4,6-[^{16}O,^{17}O,^{18}O]phosphates (**d2**). ^{1}H, ^{13}C and ^{31}P NMR chemical shifts of all isomers not containing ^{17}O are listed in **Section S.1.33** "NMR Spectroscopic Data of Methyl α/β-D-Glucopyranose-4,6-[^{16}O,^{18}O]phosphate".

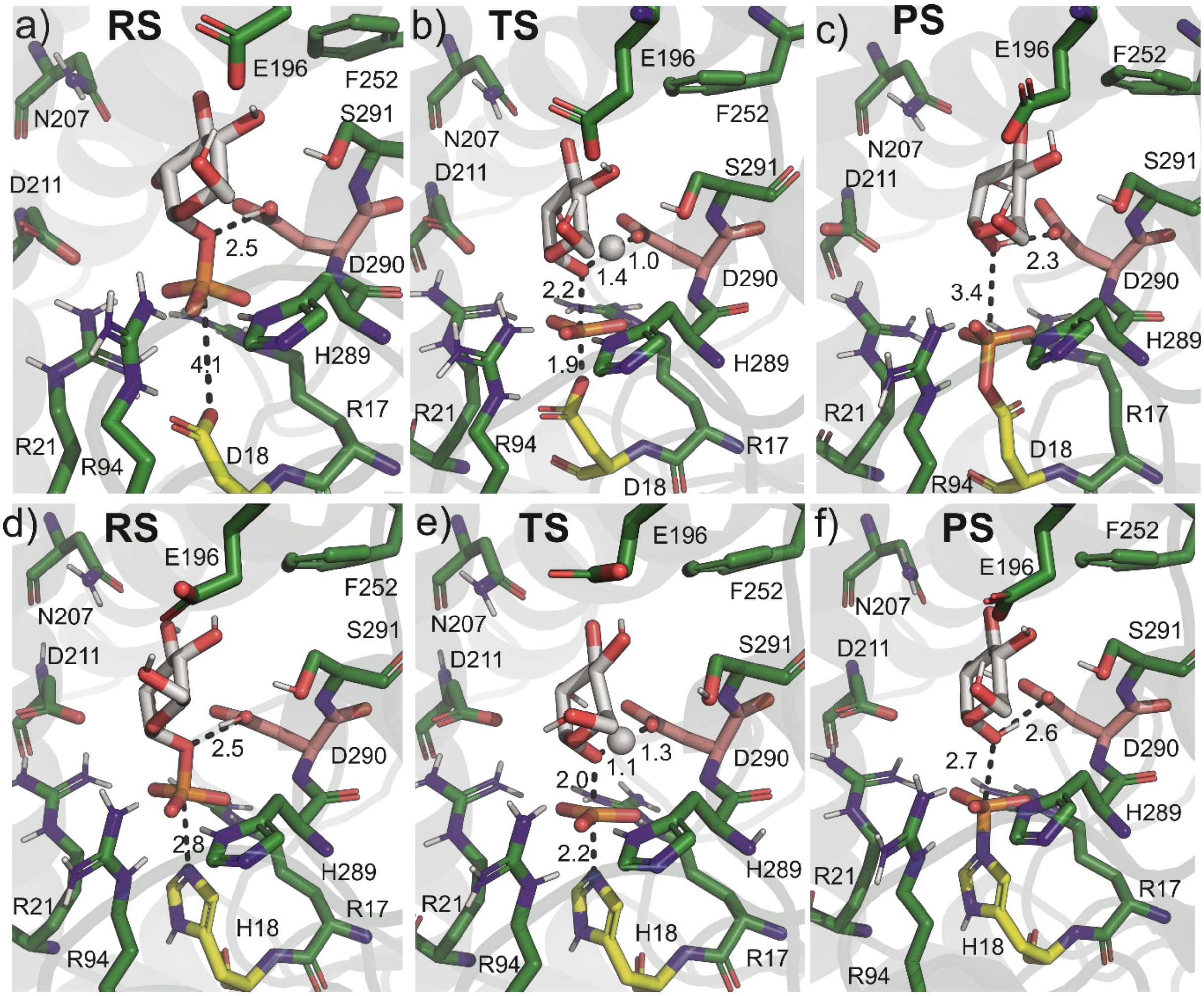

Figure S15. Representative structures of the Michaelis complex (MC), transition state (TS) and product state (PS) over the course of the reaction for the WT and H18D variant reactions with Glc1P. Key catalytic distances for each state are shown in **Table S12**. Structures were obtained by clustering the heavy atoms of the active site residues (any residue within 6 Å of the ligand) and taking the centroid (*i.e.*, most representative structure) of the most populated cluster (see **Section S1.36** "EVB Simulation Analysis", for further details).

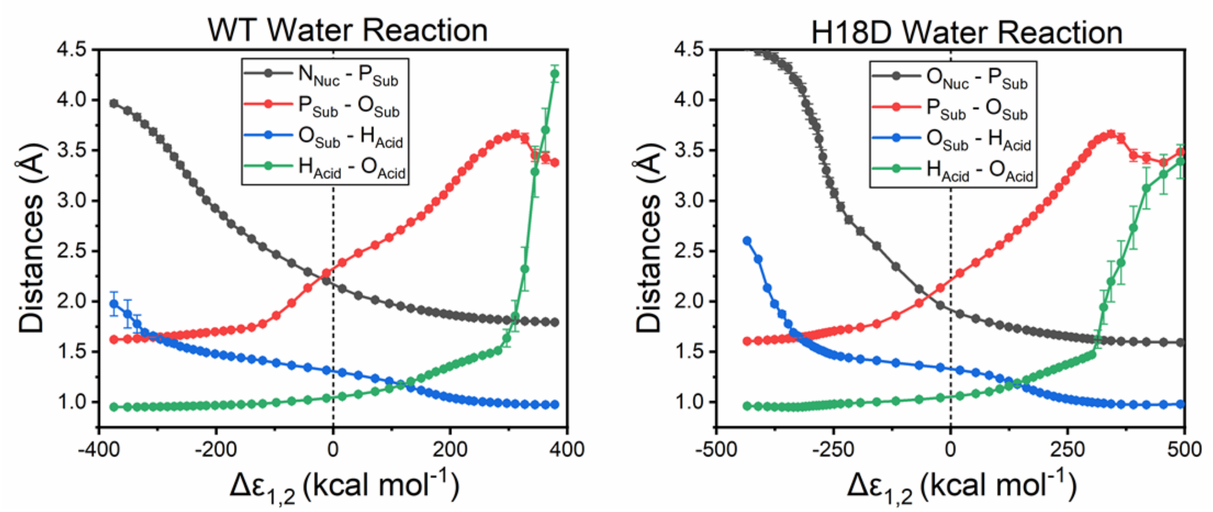

Figure S16. Change in the distances of key reacting atoms in non-enzymatic Glc1P phosphoryl transfer reactions, along the EVB energy gap reaction coordinate ($\Delta\varepsilon_{1,2}$). The approximate location of the transition state ($\Delta\varepsilon_{1,2} = 0$) is marked on both graphs with a dotted line and the points at which the reacting atoms "cross-over" (*i.e.* phosphoryl and proton transfer occur) are circled for clarity. The calculated average reacting atom distances are provided in **Table S12**. Note that our EVB calculations were performed using a 2-state model, that simultaneously describes proton transfer and phosphoryl group transfer, see **Figure S24-S25**.

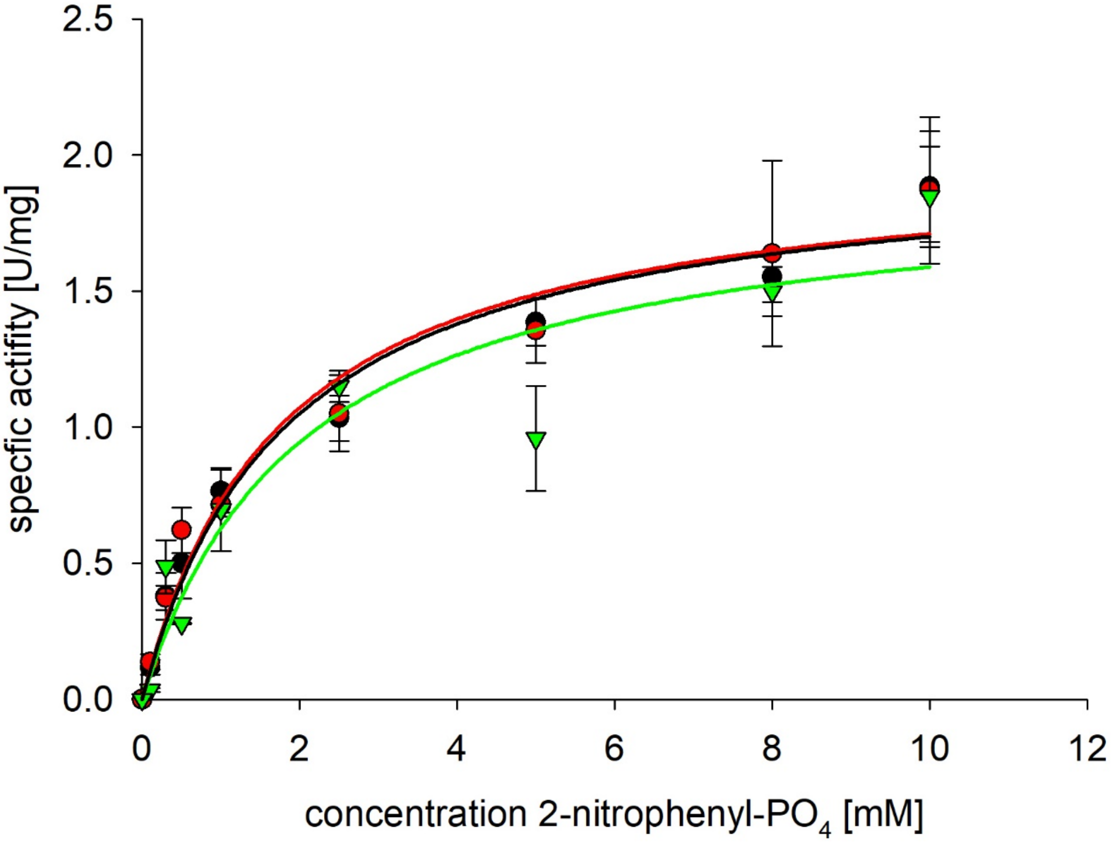

Figure S17. Analysis of inhibition by 2-nitrophenol of the ecAGP catalyzed hydrolysis of 4-nitrophenyl phosphate. Black: no 2-nitrophenol, red: 3 mM 2-nitrophenol and green: 6 mM 2-nitrophenol.

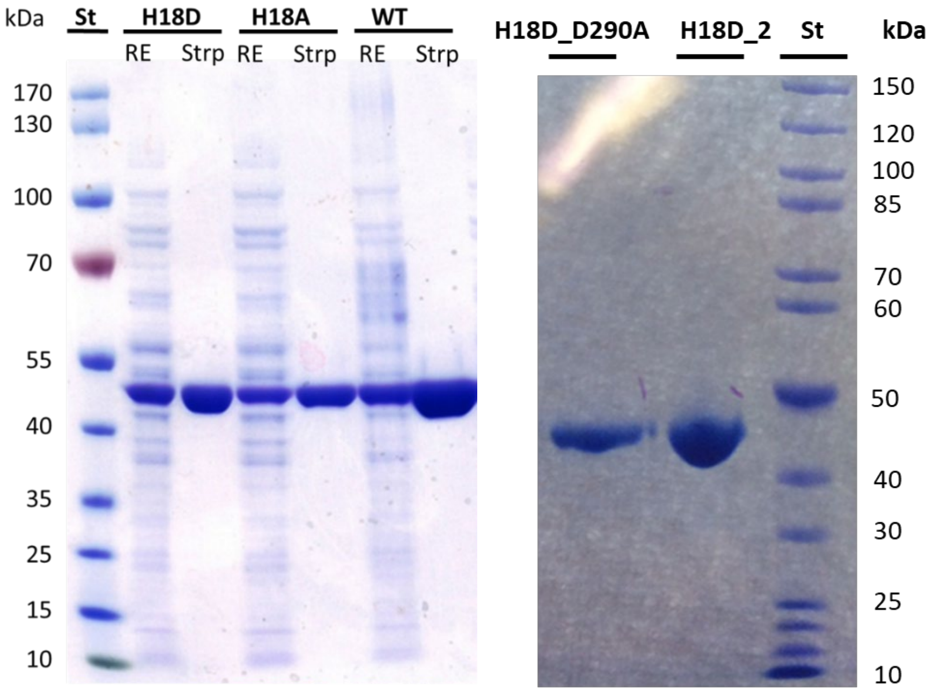

Figure S18. SDS PAGE showing the soluble fraction of the *E. coli* cell extract (RE) and the corresponding Strep-Tactin-purified enzymes used in this study.

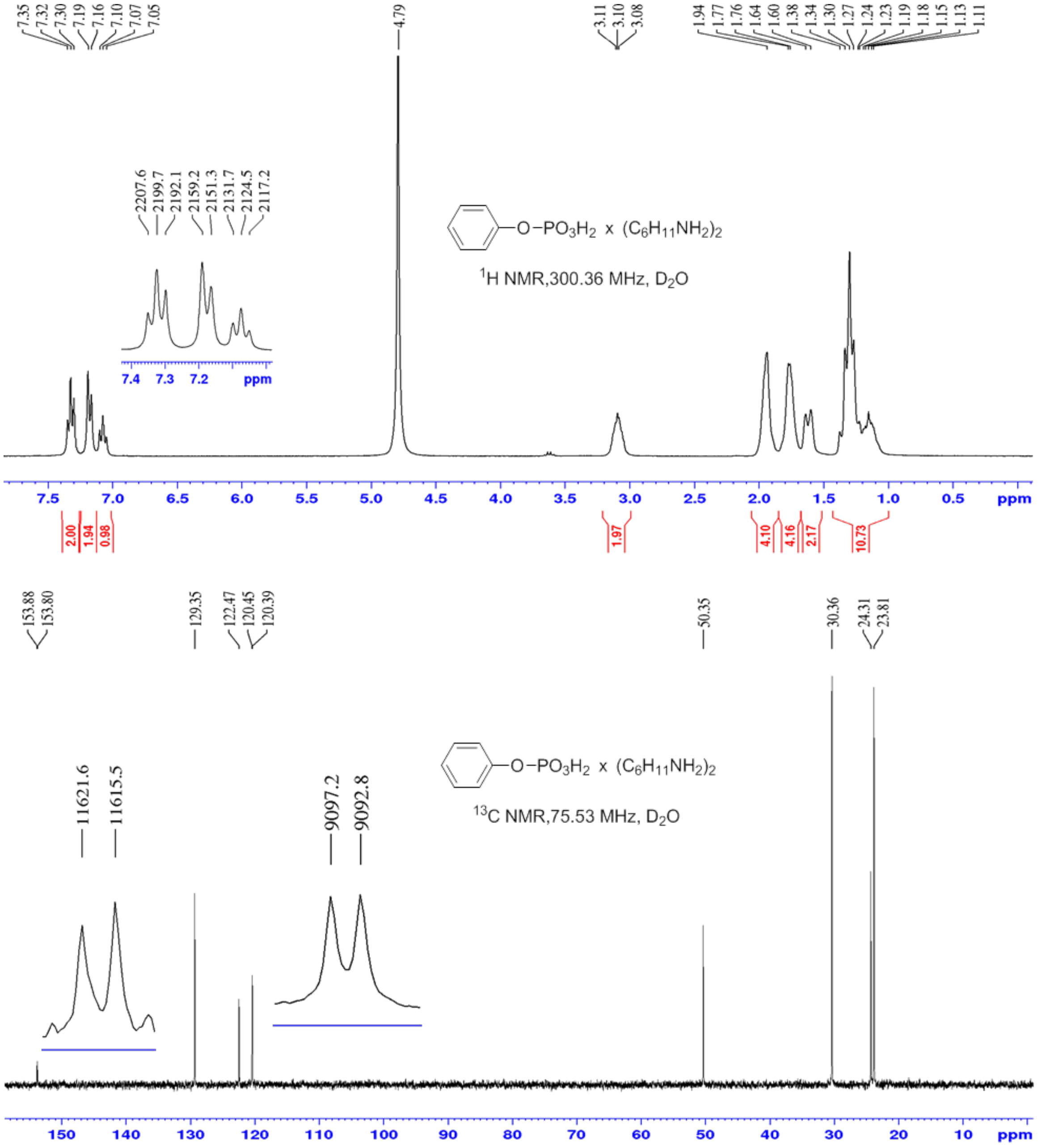

Figure S19. ^{1}H and ^{13}C NMR spectra of the bis(cyclohexylammonium) salt of phenyl phosphate. ^{1}H NMR (300.36 MHz, D_2O, δ_{HDO} = 4.79): δ 7.32 (t, *J* = 7.8 Hz, 2H), 7.17 (d, *J* = 7.8 Hz, 2H), 7.07 (t, *J* = 7.2 Hz, 1H), 3.19 – 3.00 (m, 2H), 2.03 - 1.85 (m, 4H), 1.85 – 1.69 (m, 4H), 1.69 - 1.55 (m, 2H), 1.40 – 1.03 (m, 10H), ^{13}C NMR (75.53 MHz, D_2O) δ 153.84 (d, *J* = 6.0 Hz), 129.35, 122.47, 120.42 (d, *J* = 4.5 Hz), 50.35, 30.36, 24.31, 23.81.

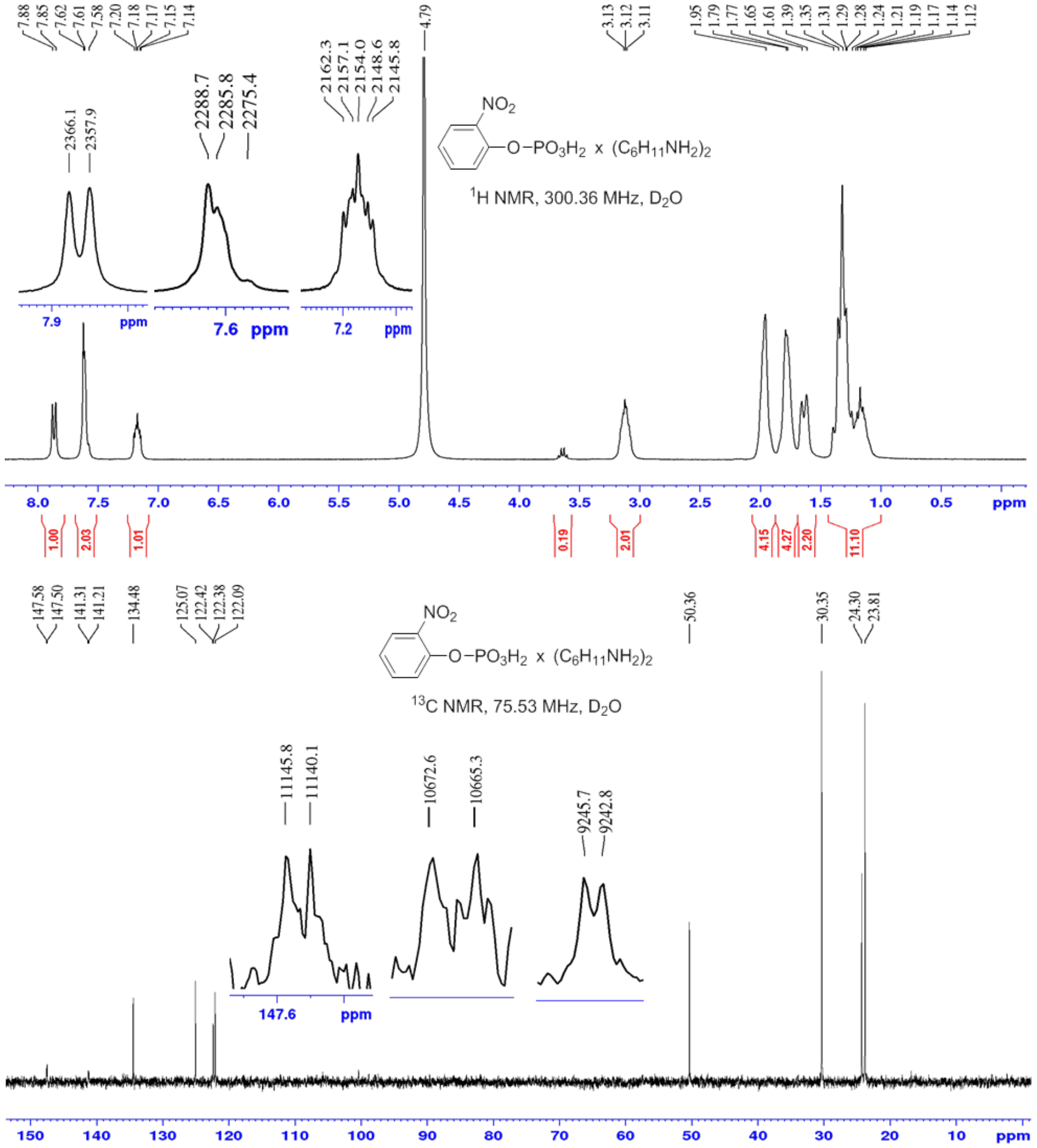

Figure S20. ^{1}H and ^{13}C NMR spectra of the bis(cyclohexylammonium) salt of 2-nitrophenyl phosphate. ^{1}H NMR (300 MHz, D_2O, δ_{HDO} = 4.79) δ 7.86 (d, *J* = 8.2 Hz, 1H), 7.68 – 7.52 (m, 2H), 7.23 – 7.10 (m, 1H), 3.23 – 3.01 (m, 2H), 2.07 – 1.87 (m, 4H), 1.87 – 1.70 (m, 4H), 1.70 – 1.52 (m, 2H), 2.07 – 1.03 (m, 10H). ^{13}C NMR (75.53 MHz, D_2O) δ 147.52 (d, *J* = 5.7 Hz), 141.25 (d, *J* = 7.4 Hz), 134.48, 125.07, 122.39 (d, *J* = 3.0 Hz), 122.09, 50.36, 30.35, 24.30, 23.81.

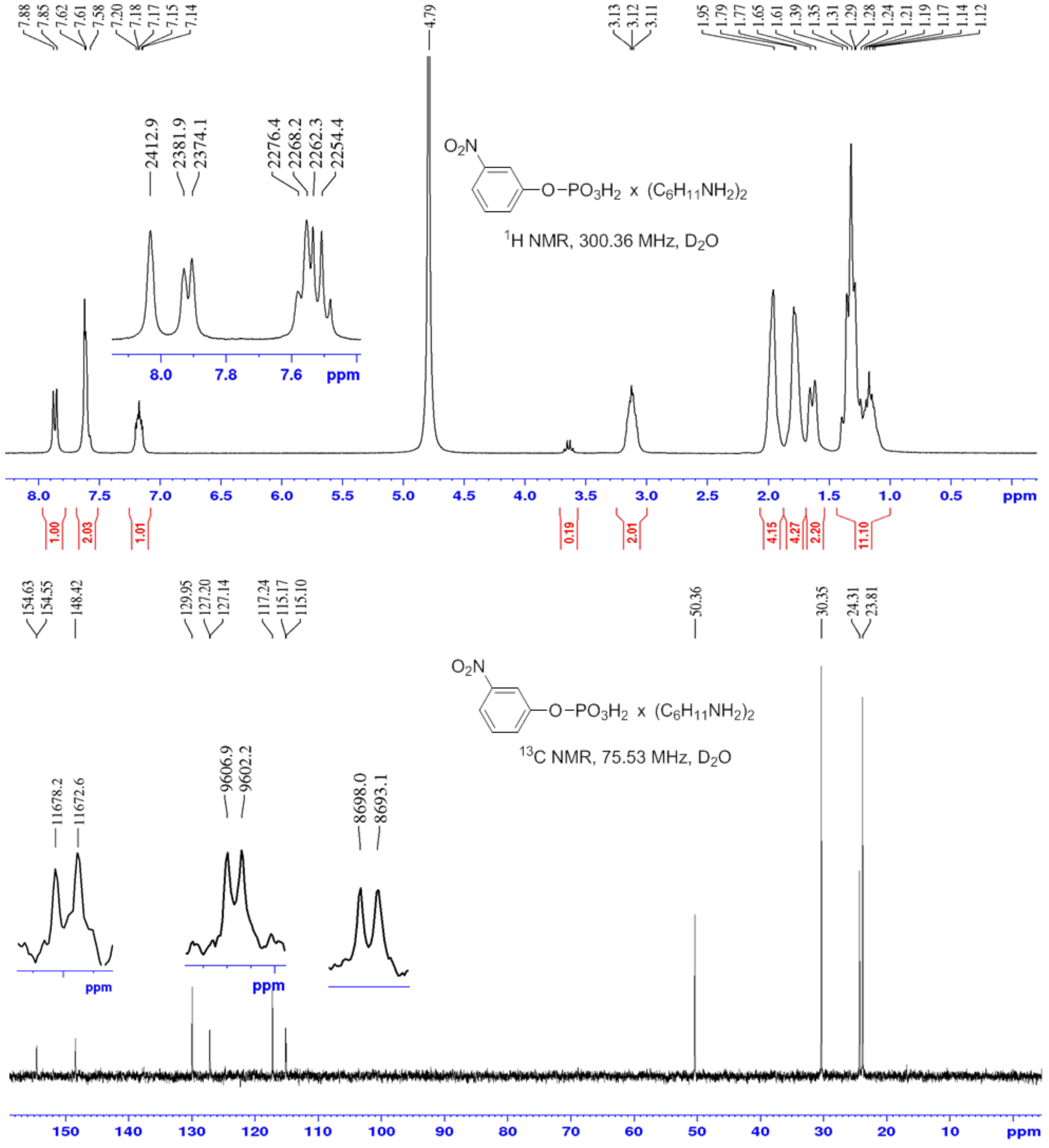

Figure S21. ^{1}H and ^{13}C NMR spectra of the bis(cyclohexylammonium) salt of 3-nitrophenyl phosphate.^{1}H NMR (300.36 MHz, D_2O, δ_{HDO} = 4.79): δ 8.03 (br.s, 1H), 7.91 (br.d, *J* = 8.0 Hz, 1H), 7.57 (br.d, *J* = 8.0 Hz, 1H), 7.51 (t, *J* = 8.0 Hz, 1H), 3.22 – 3.02 (m, 2H), 2.06 - 1.87 (m, 4H), 1.87 – 1.69 (m, 4H), 1.69 - 1.53 (m, 2H), 1.42 – 1.02 (m, 10H). ^{13}C NMR (75.53 MHz, D_2O) δ 154.58 (d, *J* = 5.6 Hz), 148.42, 129.95, 127.16 (d, *J* = 4.5 Hz), 117.24, 115.12 (d, *J* = 4.9 Hz), 50.36, 30.35, 24.31, 23.81.

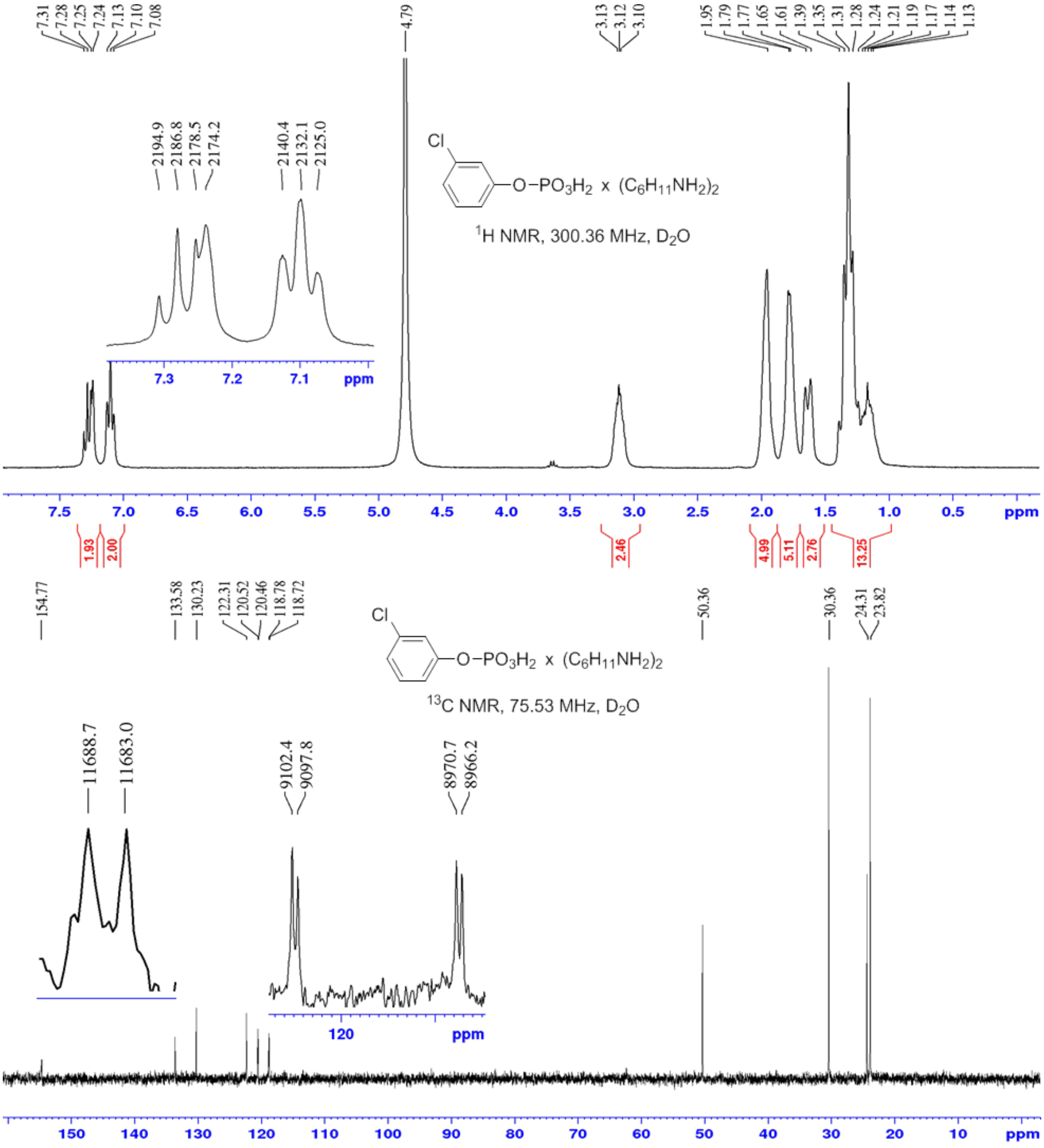

Figure S22. ^{1}H and ^{13}C NMR spectra of the bis(cyclohexylammonium) salt of 3-chlorophenyl phosphate. Possible impurities include 0.46 mol of the cyclohexylammonium salt of hydrochloric acid. ^{1}H NMR (300.36 MHz, D_2O, δ_{HDO} = 4.79): δ 7.28 (t, *J* = 8.1 Hz, 1H), 7.24 (br.s, 1H), 7.18 – 7.01 (m, 2H), 3.23 – 3.00 (m, 2H), 2.06 - 1.87 (m, 4H), 1.87 – 1.70 (m, 4H), 1.70 - 1.52 (m, 2H), 1.45 – 1.01 (m, 10H); only resonances for phosphate salt are given. ^{13}C NMR (75.53 MHz, D_2O) δ 154.72 (d, *J* = 4.7 Hz), 133.58, 130.23, 122.31, 120.48 (d, *J* = 4.6 Hz), 118.74 (d, *J* = 4.5 Hz), 50.36, 30.36, 24.31, 23.82.

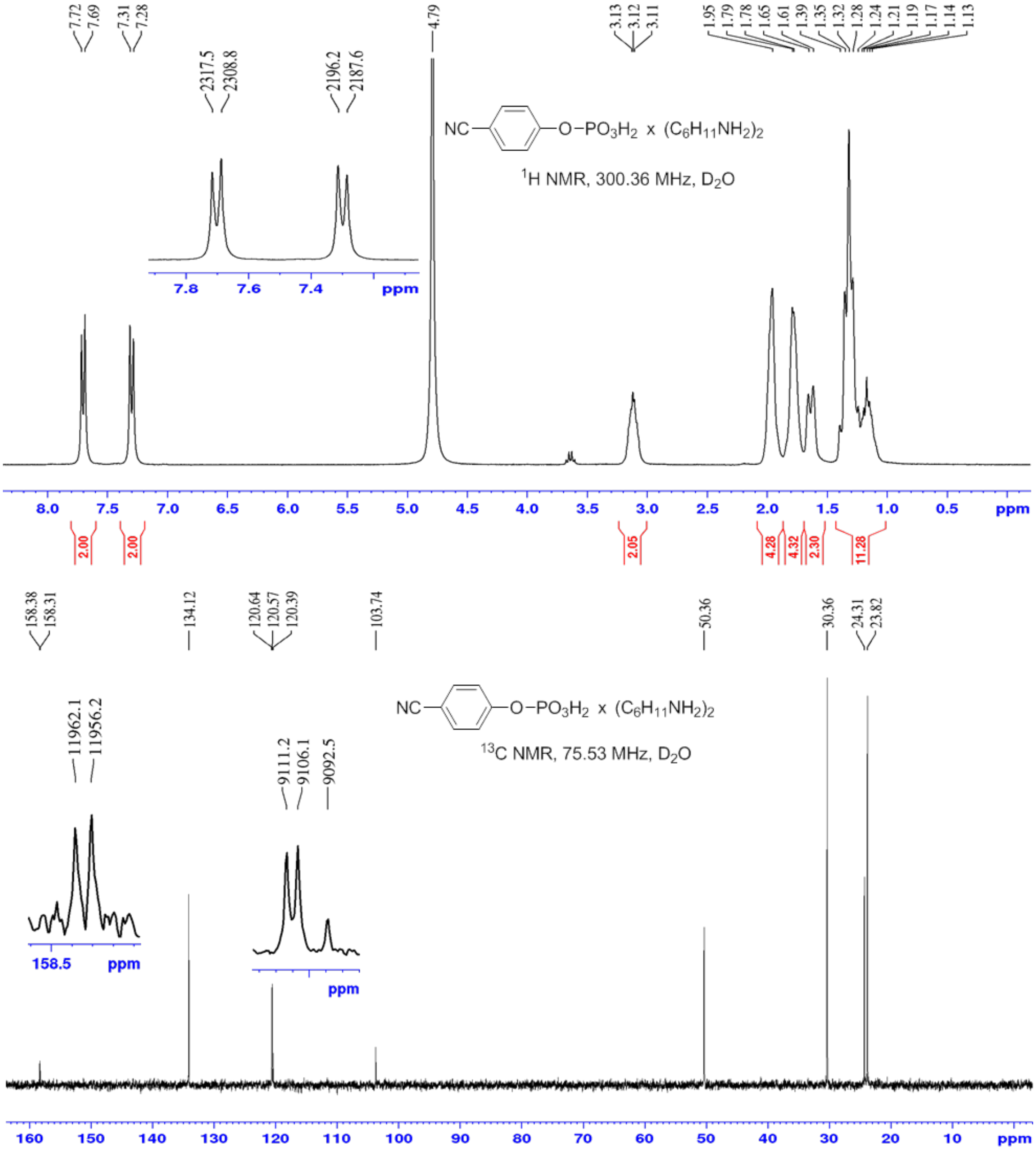

Figure S23. ¹H and ¹³C NMR spectra of the bis(cyclohexylammonium) salt of 4-cyanophenyl phosphate.¹H NMR (300.36 MHz, D_2O, δ_{HDO} = 4.79): δ 7.70 (d, *J* = 8.7 Hz, 2H), 7.30 (d, *J* = 8.7 Hz, 2H), 3.24 – 2.98 (m, 2H), 2.09 - 1.86 (m, 4H), 1.86 – 1.69 (m, 4H), 1.69 - 1.49 (m, 2H), 1.46 – 0.99 (m, 10H). ¹³C NMR (75.53 MHz, D_2O) δ 158.34 (d, *J* = 5.8 Hz), 134.12, 120.60 (d, *J* = 4.1 Hz), 120.39, 103.74, 50.36, 30.36, 24.31, 23.82.

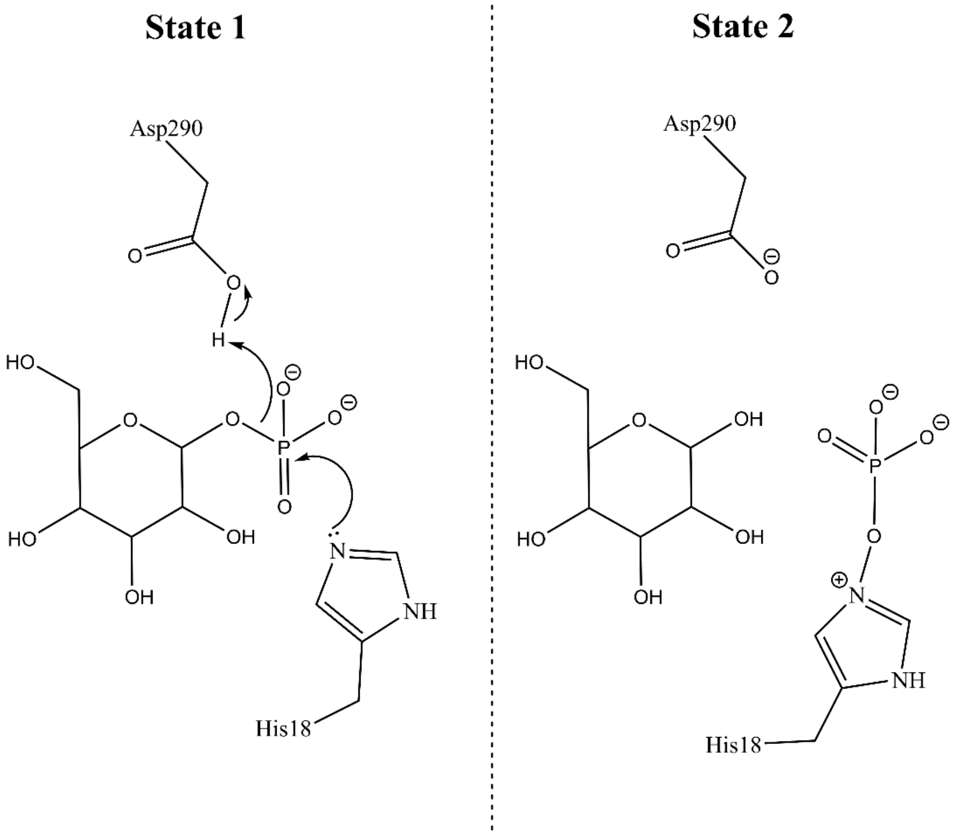

Figure S24. Valence bond states (reactant on the left, product on the right) used to describe the first chemical step (**Figure 1**, main text) catalyzed by WT ecAGP towards the Glc1P substrate. Note that for the non-enzymatic reaction, we use a truncated system whereby only the side chains of the reacting amino acid residues are used, as described in **Section S1.34** "Parameterization of the Empirical Valence Bond Simulations".

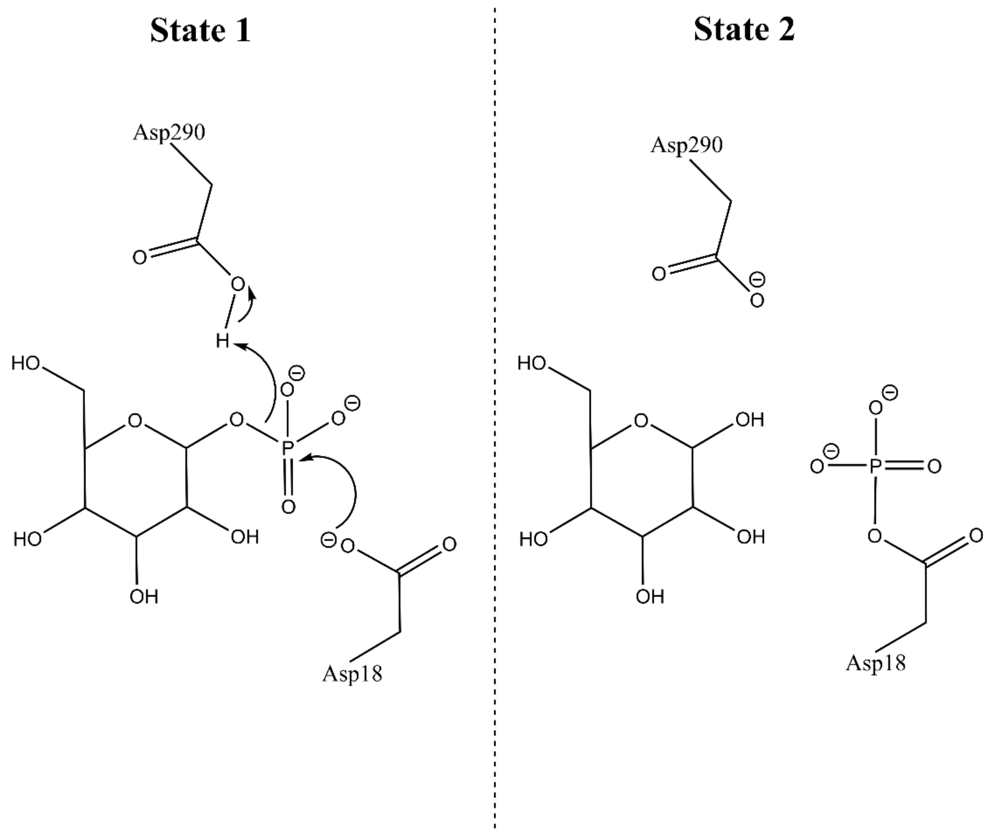

Figure S25. Valence bond states (reactant on the left, product on the right) used to describe the first chemical step (**Figure 1**, main text) catalyzed by the H18D enzyme variant of ecAGP towards the Glc1P substrate. Note that for the non-enzymatic reaction, we use a truncated system whereby only the side chains of the reacting amino acid residues are used, as described in **Section S1.34** "Parameterization of the Empirical Valence Bond Simulations).

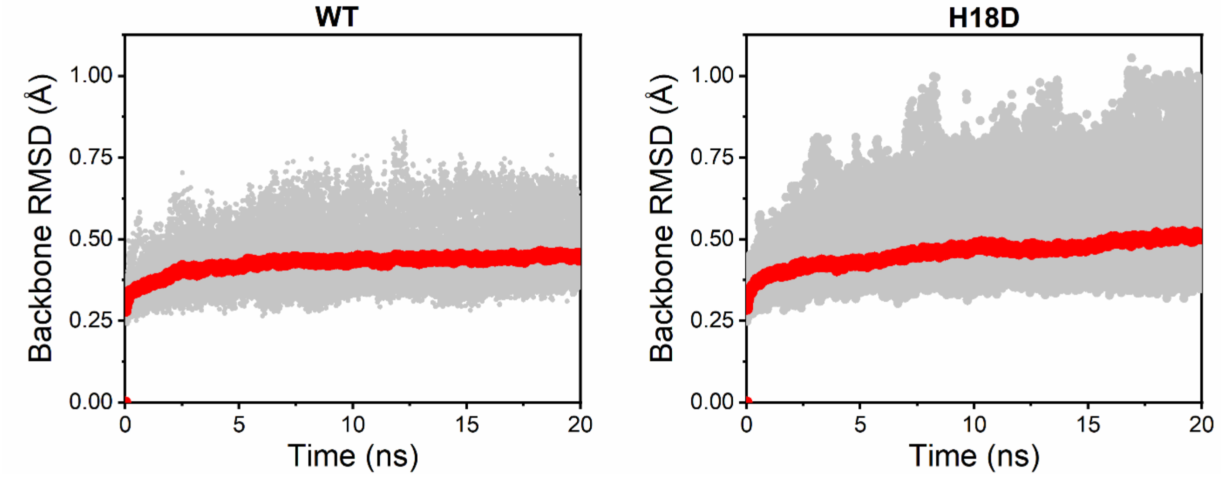

Figure S26. The root mean square deviations (RMSD, Å) of all backbone atoms during our EVB equilibration MD simulations. Equilibration simulations were performed at the approximate EVB transition states ($\lambda = 0.5$) for the reactions catalyzed by each enzyme. Data was collected every 10 ps from 30 replicas each of length 20 ns. The grey lines show the 30 individual runs, whilst the red line shows a rolling average RMSD from all 30 replicas.

S3. Supporting tables

Table S1. Active-site features of phosphatases catalyzing phosphate monoester hydrolysis *via* a covalent phospho-enzyme intermediate.

Family name	PDB ID	Catalytic nucleophile	Phosphate binding / transition state stabilization	Catalytic acid-base	Active-site net charge
Halo-alkanoic acid dehalogenase	2RB5 sugar-phosphate phosphatase	Asp8	Asp10, Ile9, Lys188, Gly44, Mg^{2+}	Asp10	+1
Phospho-tyrosine protein phosphatases	1Z12 protein tyrosine phosphatase	Cys215	Ser216, Gly217, Ala218, Ile219, Gly220, Asp181, Arg221, Gln262	Asp181	0 (neglecting dipole moment from helix)
Histidine acid phosphatase	1QWO phytase	His189	Lys115, Gly149, His150, Arg183, Arg122	Glu[88]	+3 (wild-type ecAGP) +2 (H18D)
Alkaline phosphatase	5C66 alkaline phosphatase	Ser102 alkoxide (Zn activated)	Arg166, Zn^{2+} (bound by Asp369, Asp51, His370, Asp327, His412, His331)	Zn^{2+}	+2 (Ser-O-) +3 (Ser-OH)
Histidine-dependent phosphatase	1EOI acid phosphatase	His189	Lys115, Gly149, His150, Arg183, Arg122	His150	+2

Table S2. Phosphatase activity with the natural substrate Glc1P.

ecAGP	k_{cat} (s^{-1})	K_M (mM)
WT	35 ± 1.7	0.09 ± 0.02
H18D (CAC→GAC)	0.0002 ± 0.0001	0.09 ± 0.02
H18D (CAC→GAT)	0.0002 ± 0.0001	0.12± 0.03
D290A	0.014 ± 0.01	1.06 ± 0.12
H18D_D290A	0.000043 ± 0.00001	n.d.
YidA	20 ± 1.3	0.21 ± 0.03

n.d. Not determined

Table S3. Peptides identified in YidA incubated with Glc1P that feature conversion of Asp to homoserine.[a]

Sequence	Z/score	MH+ [Da]	Modification	# Missed Cleavages	Peptide fragmentation
5-LIAIsmDGTLLLPDHTISPAVK-27	3 /14.58	2305.2	s: homoserine, m: oxidized methionine	0	L I|A|I|s m/D G/T L|L/L/P D H T I S\P A\V K

/ indicates y-fragment-ion type

\ indicates b-fragment-ion type

| indicates b and y-fragment-ion type

[a] The modified active site Asp9 residue(s) is highlighted in red.

Table S4. Peptide identified in YidA incubated in the absence of Glc1P (control).[a]

Sequence	Z/score	MH+ [Da]	Modification	# Missed Cleavages	Peptide fragmentation
5-LIAIDmDGTLLLPDHTISPAVK-27	2 /19.74	2333.3	m: oxidized methionine	0	L I/A I D/m D|G T|L|L L|P D/H T I|S\P A V\K(N)

/ indicates y-fragment-ion type

\ indicates b-fragment-ion type

| indicates b and y-fragment-ion type

[a] The peptide containing the unmodified active-site nucleophile Asp9 is detected (red).

Table S5. Peptides identified in the H18D variant incubated with Glc1P that feature conversion of Asp to homoserine.[a]

Sequence	PSMs	MH+ [Da]	ΔM [ppm]	Ion Score	Expect Value	Modification	# Missed Cleavages
18-DNLRAPLANNGSVLEQSTPNK-28	14	2238.16	3.32	104	7.56×10^{-10}		1
18-sNLRAPLANNGSVLEQSTPNKWPEWDVPGGQLTTK-52	1	3818.91	-8.84	23	9.5×10^{-2}	s: homoserine	2
18-DNLRAPLANNGSVLEQSTPNKWPEWDVPGGQLTTK-52	13	3832.93	0.30	58	3.3×10^{-5}		2
-2-GRQTVPEGYQLQQVLMMSRsNLRAPLANNGSVLE-29	1	3786.93	-0.52	28	3.3×10^{-2}	s: homoserine, m: oxidized methionine	2
-2-GRQTVPEGYQLQQVLMMSRDNLRAPLANNGSVLE-32	1	3784.92	-0.98	37	2.6×10^{-3}		2
6-GYQLQQVLMMSRDNLRAPLANNGSVLE-32	9	3017.53	1.54	44	5.3×10^{-4}		1
6-GYQLQQVLMMSRsNLRAPLANNGSVLE-32	2	3019.52	-7.87	39	1.6×10^{-3}	m: oxidized methionine; s: homoserine	1
6-GYQLQQVLMMSRDNLRAPLANNGSVLE-32	10	3033.53	0.72	47	2×10^{-4}	m: oxidized methionine	1
6-GYQLQQVLMMSRsNLRAPLANNGSVLE-32	1	3035.51	-8.82	28	1.8×10^{-2}	m: oxidized methionine; s: homoserine	1
6-GYQLQQVLMMSRDNLRAPLANNGSVLE-32	5	3049.52	-0.70	50	1.3×10^{-4}	m: oxidized methionine	1
102-FITGAFPGCDIPVHHQEKMGTMDPTFNPVITDDSAA-137	1	3916.81	3.26	44	3.7×10^{-2}	c: carbamidomethyl	0
102-FITGAFPGCDIPVHHQEKMGTMDPTFNPVITsDSAA-137	1	3918.81	-1.04	40	3.7×10^{-2}	c: carbamidomethyl; m: oxidized methionine; s: homoserine	0
102-FITGAFPGCDIPVHHQEKMGTMDPTFNPVITDDSAA-137	6	3932.82	6.81	60	6.5×10^{-4}	c: carbamidomethyl; m: oxidized methionine	0
120-MGTMDPTFNPVITDDSAAFSEK-141	48	2374.06	0.63	103	1.1×10^{-9}		0
120-MGTMDPTFNPVITsDSAAFSEK-141	2	2360.06	-9.60	64	8.5×10^{-6}	s: homoserine	0
120-MGTMDPTFNPVITDDSAAFSEK-141	80	2390.05	-1.53	118	2.8×10^{-11}	m: oxidized methionine	0
120-MGTMDPTFNPVITDDSAAFSEK-141	28	2406.04	-1.44	128	2.8×10^{-12}	m: oxidized methionine	0
120-MGTMDPTFNPVITDDSAAFSEKAVAAMEK-148	2	3074.413	-0.91	71	1.6×10^{-6}		1
120-MGTMDPTFNPVITDDSAAFSEKAVAAMEK-148	1	3090.41	1.02	87	4.1×10^{-8}	m: oxidized methionine	1
120-MGTMDPTFNPVITsDSAAFSEKAVAAMEK-148	1	3092.41	-5.74	68	3.2×10^{-6}	m: oxidized methionine	1
362-VTLELSGCPIDANGFCPMDKFDSVLNEAVK-391	22	3326.56382	-3.16	78	3.2×10^{-7}	c: carbamidomethyl	1
362-VTLELSGCPIDANGFCPMDKFsSVLNEAVK-391	3	3328.58969	-0.09	54	7.8×10^{-5}	c: carbamidomethyl; m: oxidized methionine; s: homoserine	1
362-VTLELSGCPIDANGFCPMDKFDSVLNEAVK-391	14	3342.5676	-0.49	80	2.1×10^{-7}	c: carbamidomethyl; m: oxidized methionine	1

333-sLmKIEYVYQSAEQLR-348	1	1988.01	-2.59	40	1.9×10^{-3}	s: homoserine; m: oxidized methionine	1

[a] The modified Asp residues (D) are highlighted in red. Peptides in bold letters contain the active-site nucleophile, whereas other peptides contain non-specifically modified Asp residues. (PSM = peptide spectrum matches).

Table S6. Peptides identified in the H18D variant incubated in the absence of Glc1P (negative control) that feature conversion of Asp to homoserine.[a]

Sequence	PSMs	MH+ [Da]	ΔM [ppm]	Ion Score	Expect Value	Modification	# Missed Cleavages
283-ITVLVGHDSNIASLLTALsFKPYQLHDQNER-313	1	3493.82	-6.42	27	9.3×10^{-2}	s: homoserine	0
283-ITVLVGHDSNIASLLTALDFKPYQLHDQNER-313	67	3507.84	7.08	100	4.5×10^{-9}		0
120-mGTmDPTFNPVITDDSAAFSEK-141	19	2406.05	0.79	85	4.1×10^{-8}	m: oxidized methionine	0
120-mGTMDPTFNPVITDDSAAFSEK-141	58	2390.06	2.96	110	1.4×10^{-10}	m: oxidized methionine	0
120-MGTMsPTFNPVITDDSAAFSEK-141	1	2360.07	-1.53	51	1.1×10^{-4}	s: homoserine	0
120-MGTMDPTFNPVITDDSAAFSEK-141	40	2374.06	1.25	82	8.0×10^{-8}		0
120-mGTMDPTFNPVITDDSAAFSEKAVAAMEK-148	2	3090.42	1.49	78	1.9×10^{-7}	m: oxidized methionine	1
120-MGTMDPTFNPVITDDSAAFSEKAVAAMEK-148	1	3074.42	2.19	67	2.4×10^{-6}		1

[a] The modified Asp residues (D) are highlighted in red

Table S7. Relative ratio of occurrence of different axial and equatorial methyl α/β-D-glucopyranose-4,6-[^{16}O,^{18}O]-phosphates. ¶

	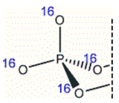	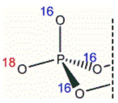	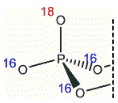	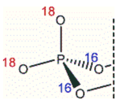
a)				
from Glc-6-(R_P)-[^{16}O,^{17}O,^{18}O]P	-	1/3 × 0% = **0.00%**	-	-
from Glc-6-(S_P)-[^{16}O,^{17}O,^{18}O]P	-	-	1/3 × 85% = **28.33%**	-
from Glc-6-[2x^{16}O,^{18}O]P	1/3 × 12% = **4.00%**	1/3 × 12% = **4.00%**	1/3 × 12% = **4.00%**	-
from Glc-6-[^{16}O,2x^{18}O]P	-	1/3 × 2% = **0.66%**	1/3 × 2% = **0.66%**	1/3 × 2% = **0.66%**
from Glc-6-[2x^{16}O,^{17}O]P	1/3 × 1% = **0.33%**	-	-	-
summed percentages	4.33%	4.66%	33.00%	0.66%
relative ratios	10.2	10.9	77.4	1.5
b)				
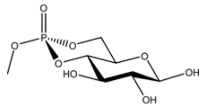	n.d.	11.8	70.3	3.9
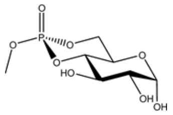	11.7	11.8	71.3	n.d.
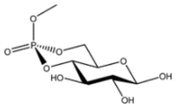	15.8	12.3	68.1	3.8
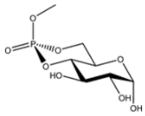	14.6	12.3	69.6	3.5
c)				
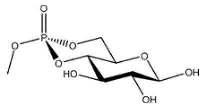	14.7	14.0	67.4	n.d.
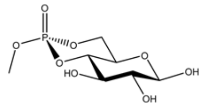	n.d.	14.0	66.4	3.4
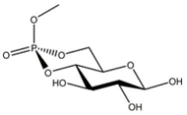	18.2	13.1	65.0	3.7
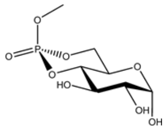	15.6	13.9	66.6	3.9

¶ Shown here are (**a**) calculated theoretical values for 100% inversion in each enzymatic step starting from (S_P)-[^{16}O,^{17}O,^{18}O]PEP as indicated in **Figure S11**. (**b**, **c**) Determined from ^{31}P NMR (**Figure S10**) after transformation with wild-type ecAGP or H18D, respectively. n.d.: not determined due to signal overlap. The observed doubly labeled (2x ^{16}O or 2x ^{18}O) cyclic phospho-methyl-esters originate from a small amount of non-uniform isotope labeling during [^{16}O,^{17}O,^{18}O]PEP synthesis and phosphoryl transfer from ATP.

Table S8. Ratio of occurrence of different axial and methyl α/β-D-glucopyranose-4,6-[^{16}O,^{18}O]-phosphates.¶

	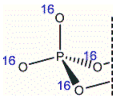	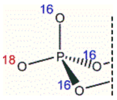	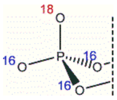	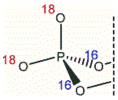
		a)		
from Glc-6-(R_P)-[^{16}O,^{17}O,^{18}O]P	-	1/3 × 85% = **28.33%**	-	-
from Glc-6-(S_P)-[^{16}O,^{17}O,^{18}O]P	-	-	1/3 × 0% = **0.00%**	-
from Glc-6-[2×^{16}O,^{18}O]P	1/3 × 12% = **4.00%**	1/3 × 12% = **4.00%**	1/3 × 12% = **4.00%**	-
from Glc-6-[^{16}O,2×^{18}O]P	-	1/3 × 2% = **0.66%**	1/3×x 2% = **0.66%**	1/3 × 2% = **0.66%**
from Glc-6-[2×^{16}O,^{17}O]P	1/3 × 1% = **0.33%**	-	-	-
summed percentages	4.33%	33.00%	4.66%	0.66%
relative ratios	10.2	77.4	10.9	1.5
		b)		
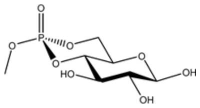	n.d.	71.8	12.8	2.5
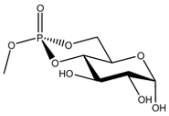	12.0	73.3	11.9	n.d.
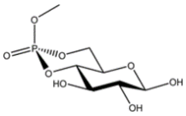	13.6	70.1	12.8	n.d
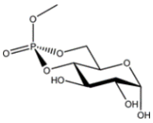	n.d.	67.4	11.7	4.0
		c)		
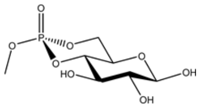	n.d.	60.0	13.4	3.4
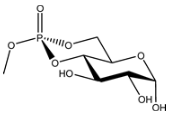	21.0	61.6	13.8	n.d.
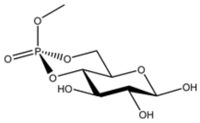	21.5	60.6	13.5	4.4
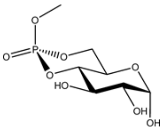	22.0	59.6	14.0	4.4

¶ Shown here are (**a**) calculated theoretical values for 100% inversion in each enzymatic step starting from (R_P)-[^{16}O,^{17}O,^{18}O]PEP as indicated in **Figure S12**. (**b**, **c**) Determined from ^{31}P NMR (Figure 2, Main text) after transformation with wild-type ecAGP or H18D, respectively. n.d.: not determined due to signal overlap. The observed doubly labeled (2x ^{16}O or 2x ^{18}O) cyclic phospho-methyl-esters originate from a small amount of non-uniform isotope labeling during [^{16}O,^{17}O,^{18}O]PEP synthesis and phosphoryl transfer from ATP.

Table S9. Data collection and refinement statistics (molecular replacement).

Data collection*	H18D
Space group	P 21 21 21
Cell dimensions	
a, *b*, *c* (Å)	64.03, 101.65, 114.92
α, β, γ (°)	90, 90, 90
Resolution (Å)	42.8 - 2.5 (2.589 - 2.5) †
R_{sym} or R_{merge}	0.1393 (1.257)
$I / \sigma I$	10.39 (1.27)
Completeness (%)	99.19 (99.12)
Redundancy	6.5 (6.8)
Refinement	
Resolution (Å)	42.8 - 2.5 (2.589 - 2.5)
No. reflections	26437
R_{work} / R_{free}	0.2245 / 0.2664
No. atoms	5913
Protein	5737
Ligand/ion	80
Water	96
B-factors	53.6
Protein	55.55
Ligand/ion	63.18
Water	54.26
R.m.s.d.	
Bond lengths (Å)	0.005
Bond angles (°)	0.70

R.m.s.d., root mean squared deviation

*One crystal was used for data collection and refinement.

†Values in parentheses are for the highest resolution shell.

Table S10. Hydrogen bonds between the active site residues and the substrate (Sub) at the reactant state (RS) and transition state (TS) complex for the wild-type (WT) enzyme.[a]

Interaction		Occupancy (%)		
Acceptor	Donor	MC	TS	Δ(MC→TS)
H18_MC	G317_MC	0.2	0.2	0.0
L20_MC	H18_SC	0.1	0.1	0.0
N19_MC	H18_SC	0.7	0.7	0.0
D290_MC	A294_MC	0.6	0.7	0.1
D290_SC	S291_MC	0.4	0.6	0.2
D290_SC	Q248_SC	0.2	0.2	0.0
D290_SC	Sub	0.1	0.5	0.4
Sub	R17_SC	1.7	2.0	0.3
Sub	R21_SC	0.9	0.9	0.0
Sub	R94_SC	1.6	1.7	0.1
Sub	H289_SC	1.3	1.5	0.2
Sub	D290_MC	0.8	0.8	0.0
Sub	S291_SC	0.4	0.4	0.0
E196_SC	Sub	0.8	0.7	-0.1
Total		9.8	11.0	1.2

[a] Hydrogen bonds are subdivided into whether they are formed by the main chain (MC) or side chain (SC) of a given residue. Reacting atom hydrogen bonds were removed from the totals. Hydrogen bonds were identified with CPPTRAJ,[53] with a hydrogen bond defined as being present if the donor-acceptor distance was ≤3.5 Å and if the donor-hydrogen-acceptor angle was 180 ± 45°. Average values are generated from 6000 snapshots (obtained from our production EVB simulations at the corresponding λ value).

Table S11. Hydrogen bonds between the active site residues and the substrate (Sub) at the reactant state (RS) and transition state (TS) complex for the H18D enzyme.[a]

Interaction		Occupancy		
Acceptor	Donor	MC	TS	Δ(MC→TS)
D18_SC	R17_SC	0.3	0.4	0.1
D18_SC	R21_SC	0.6	0.4	-0.2
D18_SC	R94_SC	1.3	0.5	-0.8
D290_SC	S291_MC	0.2	0.5	0.3
D290_MC	A294_MC	0.9	0.8	-0.1
D290_SC	Sub	0.1	0.4	0.3
Sub	R17_SC	1.5	1.1	-0.4
Sub	R21_SC	1.1	1.0	-0.1
Sub	R94_SC	1.0	1.5	0.5
Sub	H289_SC	0.8	0.9	0.1
Sub	S291_SC	0.7	0.6	-0.1
E196_SC	Sub	1.0	0.7	-0.3
G1P	D290_MC	0.8	0.7	-0.1
Total		10.3	9.5	-0.8

[a] Hydrogen bonds are subdivided into whether they are formed by the main chain (MC) or side chain (SC) of a given residue. Reacting atom hydrogen bonds were removed from the totals. Hydrogen bonds were identified with CPPTRAJ,[53] with a hydrogen bond defined as being present if the donor-acceptor distance was ≤ 3.5 Å and if the donor-hydrogen-acceptor angle was 180 ± 45°. Average values are generated from 6000 snapshots (obtained from our production EVB simulations at the corresponding λ value).

Table S12. Calculated distances (Å) at the Michaelis complexes (MC), transition states (TS) and product states (PS) obtained from our EVB simulations of wild-type (WT) and H18D ecAGP in complex with the substrate Glc1P.[a]

System		Catalytic Distances			
		$N_{nuc}/O_{nuc} - P$	$P - O_{lg}$	$O_{lg} - H_{acid}$	$H_{acid} - O_{acid}$
Wild-type Enzyme (Histidine Nucleophile)					
Non-Enzymatic Reaction (His nucleophile)	MC	3.83 ± 0.05	1.63 ± 0.01	2.01 ± 0.11	0.95 ± 0.01
	TS	2.08 ± 0.02	2.15 ± 0.02	1.29 ± 0.02	1.06 ± 0.01
	PS	1.81 ± 0.01	3.48 ± 0.05	0.98 ± 0.01	1.85 ± 0.14
WT	MC	3.03 ± 0.03	1.65 ± 0.01	1.60 ± 0.05	0.98 ± 0.01
	TS	2.16 ± 0.02	1.97 ± 0.02	1.10 ± 0.01	1.29 ± 0.01
	PS	1.81 ± 0.01	3.05 ± 0.04	0.97 ± 0.01	1.81 ± 0.11
H18D Variant (Aspartic acid Nucleophile)					
Non-Enzymatic Reaction (Asp nucleophile)	MC	4.15 ± 0.17	1.64 ± 0.03	1.66 ± 0.15	0.95 ± 0.03
	TS	1.97 ± 0.11	2.13 ± 0.14	1.35 ± 0.08	1.02 ± 0.05
	PS	1.62 ± 0.03	3.62 ± 0.25	0.99 ± 0.03	1.52 ± 0.09
H18D	MC	4.18 ± 0.11	1.64 ± 0.01	2.14 ± 0.15	0.95 ± 0.01
	TS	1.99 ± 0.02	2.12 ± 0.03	1.34 ± 0.01	1.02 ± 0.01
	PS	1.61 ± 0.01	3.34 ± 0.06	0.97 ± 0.01	1.63 ± 0.11

[a] The non-enzymatic (reference) reactions catalytic distances are also shown. Errors shown are standard error of the mean obtained from the 30 replicas performed per system. $N_{nuc}/O_{nuc} - P$ denotes the distance between the His/Asp side chain and the phosphorus atom of the phosphate group, $P - O_{lg}$ denotes the distance between the phosphorus atom and the leaving group oxygen in the cleavage step, $O_{lg} - H_{acid}$ denotes the distance between the leaving group oxygen atom and the hydrogen atom being donated by the D290. $H_{acid} - O_{acid}$ denotes the distance between the oxygen and hydrogen atoms on D290.

Table S13. Electrostatic contributions of individual amino acids ($\Delta\Delta G^{\ddagger}_{elec}$, kcal mol^{-1}) to the calculated activation free energies for the reaction catalyzed by both the wild-type (WT) and H18D enzyme variant.[a]

Amino Acid	WT	H18D
R17	-2.6 ± 0.2	-2.5 ± 0.9
N19	-0.3 ± 0.03	0.9 ± 0.1
R21	1.3 ± 0.1	3.4 ± 0.5
D43	-0.7 ± 0.02	-0.8 ± 0.03
K52	0.4 ± 0.01	0.6 ± 0.02
E57	-1.4 ± 0.03	-2.0 ± 0.04
R94	6.3 ± 0.1	8.0 ± 0.5
E118	-0.5 ± 0.01	-0.5 ± 0.02
K119	0.6 ± 0.01	0.6 ± 0.02
D124	-0.7 ± 0.02	-0.7 ± 0.03
E196	-1.3 ± 0.1	-1.4 ± 0.1
N207	0.6 ± 0.1	0.4 ± 0.04
D211	1.7 ± 0.1	2.2 ± 0.1
E219	-0.7 ± 0.02	-0.8 ± 0.04
K244	-6.0 ± 0.3	-7.1 ± 0.3
N245	0.7 ± 0.01	0.7 ± 0.02
Q248	0.9 ± 0.3	0.9 ± 0.2
D249	2.4 ± 0.02	2.4 ± 0.1
G288	-1.4 ± 0.02	-1.1 ± 0.02
H289	-0.2 ± 0.1	-0.7 ± 0.2
S291	-1.4 ± 0.1	-1.2 ± 0.02
A294	-0.6 ± 0.01	-0.6 ± 0.01
E312	3.1 ± 0.2	3.6 ± 0.2
R313	-2.3 ± 0.1	-2.4 ± 0.1
T314	0.7 ± 0.1	0.9 ± 0.1
K319	1.1 ± 0.1	1.6 ± 0.1

[a] Data was obtained from our calculated EVB trajectories using the linear response approximation (LRA)[60] and is presented as average values and standard error of the mean over 30 individual trajectories per system. Only residues with contributions of ≥0.5 kcal mol^{-1} are shown here for clarity. This data is presented visually in **Figure 4**.

Table S14. Catalytic efficiency of wild-type ecAGP, H18D, D290A and YidA variants.[a]

Substrate	pK_a	WT<br>k_{cat}/K_m (M^{-1} s^{-1})	D290A<br>k_{cat}/K_m (M^{-1} s^{-1})	H18D<br>k_{cat}/K_m (M^{-1} s^{-1})	YidA<br>k_{cat}/K_m (M^{-1} s^{-1})	H18D_2<br>k_{cat}/K_m (M^{-1} s^{-1})
4-NO_2-Ph-P	7.14	$1.39 \times 10^3 \pm 0.70 \times 10^3$	$2.76 \times 10^4 \pm 3.0 \times 10^3$	$2.7 \times 10^{-1} \pm 6.0 \times 10^{-2}$	$2.7 \times 10^2 \pm 9.0 \times 10^1$	$6.2 \times 10^{-1} \pm 3.12 \times 10^{-1}$
2-NO_2-Ph-P	7.23	$4.32 \times 10^3 \pm 0.63 \times 10^3$	$9.47 \times 10^3 \pm 1.3 \times 10^3$	$3.0 \times 10^{-1} \pm 0.1$	$1.9 \times 10^2 \pm 5.0 \times 10^1$	$2.9 \times 10^{-1} \pm 8.0 \times 10^{-2}$
4-CN-Ph-P	7.95	$1.34 \times 10^3 \pm 0.34 \times 10^3$	$4.01 \times 10^3 \pm 5.7 \times 10^2$	$3.0 \times 10^{-2} \pm 0.01$	$6.0 \times 10^1 \pm 1.0 \times 10^1$	
3-NO_2-Ph-P	8.38	$2.13 \times 10^3 \pm 0.29 \times 10^3$	$6.14 \times 10^3 \pm 6.1 \times 10^2$	$5.0 \times 10^{-2} \pm 0.02$	$1.6 \times 10^2 \pm 3.0 \times 10^1$	
3-Cl-Ph-P	9.08	$1.77 \times 10^3 \pm 0.15 \times 10^3$	$4.10 \times 10^2 \pm 7.0 \times 10^1$	$5.0 \times 10^{-2} \pm 0.01$	$3.6 \times 10^2 \pm 5.0 \times 10^1$	
Ph-P	10	$1.63 \times 10^3 \pm 0.26 \times 10^3$	$4.0 \times 10^1 \pm 1.0 \times 10^1$	$3.0 \times 10^{-2} \pm 0.01$	$2.6 \times 10^2 \pm 4.0 \times 10^1$	
Glc1P	12	$3.89 \times 10^5 \pm 8 \times 10^4$	$2.2 \times 10^1 \pm 5 \times 10^0$	$2.0 \times 10^1 \pm 1$	$9.5 \times 10^4 \pm 1.3 \times 10^4$	$2 \times 10^1 \pm 1.0 \times 10^1$
Glc6P	14.6	$8.81 \times 10^4 \pm 1.4 \times 10^4$	$1.3 \times 10^1 \pm 2 \times 10^0$	$2.0 \times 10^2 \pm 0.02$	$1.4 \times 10^4 \pm 1.5 \times 10^3$	

[a] Errors are standard deviations from measurements performed in triplicate.

Table S15. List of ionized residues and histidine protonation patterns used in EVB simulations.[a]

Residue Name	Residue Number
Asp	43, 124, 211, 249, 309, 351
Glu	41, 57, 118, 196, 219, 312, 338, 345
Lys	52, 119, 192, 204, 263, 271, 319, 336
Arg	17, 21, 65, 94, 348, 361
His-ε[b]	62, 115
His-δ[c]	116, 289, 308, 326

[a] All other ionizable residues not specified in the table were simulated in their neutral state during the simulations, as they fell outside the explicit solvent sphere (see **Section S1.33** "System Preparation for the Empirical Valence Bond Simulations"). [b] His-ε corresponds to a histidine singly protonated on its $N_{\epsilon 2}$ nitrogen atom. [c] His-δ corresponds to a histidine singly protonated on its $N_{\delta 1}$ nitrogen atom.

Table S16. EVB mapping parameters used to describe wild-type (WT) and H18D catalyzed reactions. [a]

Reaction	$\Delta G^{\ddagger}$ (kcal mol^{-1})	ΔG_o (kcal mol^{-1})	H_{ij}	α
WT non-enzymatic reaction	26.5 ± 0.06	2.6 ± 0.08	128.3	17.5
H18D non-enzymatic reaction	34.2 ± 0.05	10.3 ± 0.10	155.3	1.6

[a] The values presented are the averages and standard errors of the mean for the calculated activation ($\Delta G^{\ddagger}$) and reaction (ΔG_0) free energies for the non-enzymatic model systems of both the WT and H18D variant, and the values of H_{ij} and α are calibrated to reproduce these energies. The calibration procedure is described in **Section S1.34** "Parameterization of the Empirical Valence Bond Simulations". For a detailed description of the physical meaning of these parameters, see *e.g.,* refs. [38,61] .

S4. Supporting references

(1) Wildberger, P.; Pfeiffer, M.; Brecker, L.; Rechberger, G. N.; Birner-Gruenberger, R.; Nidetzky, B. Phosphoryl Transfer from α-D-Glucose 1-Phosphate Catalyzed by *Escherichia Coli* Sugar-Phosphate Phosphatases of Two Protein Superfamily Types. *Appl. Environ. Microbiol.* **2015**, *81* ,1559–1572.

(2) Quan, J.; Tian, J. Circular Polymerase Extension Cloning for High-Throughput Cloning of Complex and Combinatorial DNA Libraries. *Nat. Protoc.* **2011**, *6*, 242–251.

(3) Edelheit, O.; Hanukoglu, A.; Hanukoglu, I. Simple and Efficient Site-Directed Mutagenesis Using Two Single-Primer Reactions in Parallel to Generate Mutants for Protein Structure-Function Studies. *BMC Biotechnol.* **2009**, *9*, 61.

(4) Pfeiffer, M.; Johansson, C.; Krojer, T.; Kavanagh, K. L.; Oppermann, U.; Nidetzky, B. A Parsimonious Mechanism of Sugar Dehydration by Human GDP-Mannose-4,6-Dehydratase. *ACS Catal.* **2019**, *9*, 2962–2968.

(5) Saheki, S.; Takeda, A.; Shimazu, T. Assay of Inorganic Phosphate in the Mild PH Range, Suitable for Measurement of Glycogen Phosphorylase Activity. *Anal. Biochem.* **1985**, *148*, 277–281.

(6) Eis, C.; Nidetzky, B. Characterization of Trehalose Phosphorylase from Schizophyllum Commune. *Biochem J.* **1999**, *341*, 385–393.

(7) Gasteiger, E.; Hoogland, C.; Gattiker, A.; Wilkins, M. R.; Appel, R. D.; Bairoch, A.; others. Protein Identification and Analysis Tools on the ExPASy Server. In *The proteomics protocols handbook*; Springer, 2005; 571–607.

(8) Kabsch, W. XDS. *Acta Crystallogr. D Biol. Crystallogr.* **2010**, *66*, 125–132.

(9) McCoy, A. J.; Grosse-Kunstleve, R. W.; Adams, P. D.; Winn, M. D.; Storoni, L. C.; Read, R. J. Phaser Crystallographic Software. *J. Appl. Crystallogr.* **2007**, *40*, 658–674.

(10) Emsley, P.; Lohkamp, B.; Scott, W. G.; Cowtan, K. Features and Development of Coot. *Acta Crystallogr. D Biol. Crystallogr.* **2010**, *66*, 486–501.

(11) Adams, P. D.; Afonine, P. V.; Bunkóczi, G.; Chen, V. B.; Davis, I. W.; Echols, N.; Headd, J. J.; Hung, L.-W.; Kapral, G. J.; Grosse-Kunstleve, R. W.; McCoy, A. J.; Moriarty, N. W.; Oeffner, R.; Read, R. J.; Richardson, D. C.; Richardson, J. S.; Terwilliger, T. C.; Zwart, P. H. PHENIX: A Comprehensive Python-Based System for Macromolecular Structure Solution. *Acta Crystallogr. D Biol. Crystallogr.* **2010**, *66*, 213–221.

(12) Chen, V. B.; Arendall, W. B.; Headd, J. J.; Keedy, D. A.; Immormino, R. M.; Kapral, G. J.; Murray, L. W.; Richardson, J. S.; Richardson, D. C. MolProbity: All-Atom Structure Validation for Macromolecular Crystallography. *Acta Crystallogr. D Biol. Crystallogr.* **2010**, *66*, 12–21.

(13) Schrödinger, LLC. The AxPyMOL Molecular Graphics Plugin for Microsoft PowerPoint, Version 1.8, 2015.

(14) Eargle, D. H.; Ličko, V.; Kenyon, G. L. Kinetic Studies of ^{18}O Exchange of Inorganic Phosphate Using Mass Spectral Measurements on the Tris-(Trimethylsilyl) Derivative. *Anal. Biochem.* **1977**, *81*, 186–195.

(15) Cronin, A.; Homburg, S.; Dürk, H.; Richter, I.; Adamska, M.; Frère, F.; Arand, M. Insights into the Catalytic Mechanism of Human SEH Phosphatase by Site-Directed Mutagenesis and LC-MS/MS Analysis. *J. Mol. Biol.* **2008**, *383*, 627–640.

(16) Shevchenko, A.; Wilm, M.; Vorm, O.; Mann, M. Mass Spectrometric Sequencing of Proteins from Silver-Stained Polyacrylamide Gels. *Anal. Chem.* **1996**, *68*, 850–858.

(17) Gross, J. W.; Hegeman, A. D.; Vestling, M. M.; Frey, P. A. Characterization of Enzymatic Processes by Rapid Mix−Quench Mass Spectrometry: The Case of dTDP-Glucose 4,6-Dehydratase. *Biochemistry* **2000**, *39*, 13633–13640.

(18) Hall, A. D.; Williams, A. Leaving Group Dependence in the Phosphorylation of *Escherichia Coli* Alkaline Phosphatase by Monophosphate Esters. *Biochemistry* **1986**, *25*, 4784–4790.
(19) Malová Križková, P.; Prechelmacher, S.; Roller, A.; Hammerschmidt, F. Chemical Synthesis of (*R*P)- and (*S*P)-[^{16}O,^{17}O,^{18}O]Phosphoenol Pyruvate. *J. Org. Chem.* **2017**, *82*, 10310–10318.
(20) Jarvest, R. L.; Lowe, G.; Potter, B. V. L. Analysis of the Chirality of [^{16}O,^{17}O,^{18}O] Phosphate Esters by ^{31}P Nuclear Magnetic Resonance Spectroscopy. *J. Chem. Soc. Perkin 1* **1981**, 3186–3195.
(21) Seidel, H. M.; Freeman, S.; Schwalbe, C. H.; Knowles, J. R. Phosphonate Biosynthesis: The Stereochemical Course of Phosphoenolpyruvate Mutase. *J. Am. Chem. Soc.* **1990**, *112*, 8149–8155.
(22) Malová Križková, P.; Prechelmacher, S.; Roller, A.; Hammerschmidt, F. Chemical Synthesis of (*R*P)- and (*S*P)-[^{16}O,^{17}O,^{18}O]Phosphoenol Pyruvate. *J. Org. Chem.* **2017**, *82*, 10310–10318.
(23) Kapeller, D. C.; Hammerschmidt, F. Enantiopure Chiral (2,4,6-Triisopropylbenzoyl)Oxy-[D1]Methyllithium: Configurational Stability, Reactions, and Mechanistic Studies. *J. Org. Chem.* **2009**, *74*, 2380–2388.
(24) Barluenga, J.; Flórez, J.; Yus, M. β-Substituted Organolithium Compounds from Chlorohydrins: Application to the Direct Synthesis of Bifunctionalized Organic Compounds. *J. Chem. Soc. Perkin 1* **1983**, *0*, 3019–3026.
(25) Streiff, S.; Ribeiro, N.; Désaubry, L. Unexpected Cleavage of Tetrahydrofuran by Catalytic Reductive Lithiation. *Chem. Commun.* **2004**, *0*, 346–347.
(26) Müller, D. S.; Untiedt, N. L.; Dieskau, A. P.; Lackner, G. L.; Overman, L. E. Constructing Quaternary Stereogenic Centers Using Tertiary Organocuprates and Tertiary Radicals. Total Synthesis of Trans-Clerodane Natural Products. *J. Am. Chem. Soc.* **2015**, *137*, 660–663.
(27) Shen, Z.-L.; Sommer, K.; Knochel, P. Expedient Preparation of Aryllithium and Arylzinc Reagents from Aryl Chlorides Using Lithium 4,4′-Di-Tert-Butylbiphenylide and Zinc(II) Chloride. *Synthesis* **2015**, *47*, 2617–2630.
(28) Tay, G. C.; Gesinski, M. R.; Rychnovsky, S. D. Formation of Highly Substituted Tetrahydropyranones: Application to the Total Synthesis of Cyanolide A. *Org. Lett.* **2013**, *15*, 4536–4539.
(29) Cahn, R. S.; Ingold, C.; Prelog, V. Specification of Molecular Chirality. *Angew. Chem. Int. Ed. Engl.* **1966**, *5*, 385–415.
(30) Bauer, P.; Barrozo, A.; Purg, M.; Amrein, B. A.; Esguerra, M.; Wilson, P. B.; Major, D. T.; Åqvist, J.; Kamerlin, S. C. L. Q6: A Comprehensive Toolkit for Empirical Valence Bond and Related Free Energy Calculations. *SoftwareX* **2018**, *7*, 388–395.
(31) Lee, D. C.; Cottrill, M. A.; Forsberg, C. W.; Jia, Z. Functional Insights Revealed by the Crystal Structures of *Escherichia Coli* Glucose-1-Phosphatase. *J. Biol. Chem.* **2003**, *278*, 31412–31418.
(32) Trott, O.; Olson, A. J. AutoDock Vina: Improving the Speed and Accuracy of Docking with a New Scoring Function, Efficient Optimization, and Multithreading. *J. Comput. Chem.* **2010**, *31*, 455–461.
(33) Eberhardt, J.; Santos-Martins, D.; Tillack, A. F.; Forli, S. AutoDock Vina 1.2.0: New Docking Methods, Expanded Force Field, and Python Bindings. *J. Chem. Inf. Model.* **2021**, *61* (8), 3891–3898. https://doi.org/10.1021/acs.jcim.1c00203.
(34) Jorgensen, W. L.; Chandrasekhar, J.; Madura, J. D.; Impey, R. W.; Klein, M. L. Comparison of Simple Potential Functions for Simulating Liquid Water. *J. Chem. Phys.* **1983**, *79* (2), 926–935. https://doi.org/10.1063/1.445869.
(35) King, G.; Warshel, A. A Surface Constrained All-atom Solvent Model for Effective Simulations of Polar Solutions. *J. Chem. Phys.* **1989**, *91*, 3647–3661.

(36) Søndergaard, C. R.; Olsson, M. H. M.; Rostkowski, M.; Jensen, J. H. Improved Treatment of Ligands and Coupling Effects in Empirical Calculation and Rationalization of pK_a Values. *J. Chem. Theory Comput.* **2011**, *7*, 2284–2295.
(37) Warshel, A.; Weiss, R. M. An Empirical Valence Bond Approach for Comparing Reactions in Solutions and in Enzymes. *J. Am. Chem. Soc.* **1980**, *102*, 6218–6226.
(38) Shurki, A.; Derat, E.; Barrozo, A.; Kamerlin, S. C. L. How Valence Bond Theory Can Help You Understand Your (Bio)Chemical Reaction. *Chem. Soc. Rev.* **2015**, *44*, 1037–1052.
(39) Rosta, E.; Warshel, A. Origin of Linear Free Energy Relationships: Exploring the Nature of the Off-Diagonal Coupling Elements in SN2 Reactions. *J. Chem. Theory Comput.* **2012**, *8*, 3574–3585.
(40) Hong, G.; Rosta, E.; Warshel, A. Using the Constrained DFT Approach in Generating Diabatic Surfaces and Off Diagonal Empirical Valence Bond Terms for Modeling Reactions in Condensed Phases. *J. Phys. Chem. B* **2006**, *110*, 19570–19574.
(41) Gerstein, Joseph.; Jencks, W. P. Equilibria and Rates for Acetyl Transfer among Substituted Phenyl Acetates, Acetylimidazole, O-Acylhydroxamic Acids, and Thiol Esters. *J. Am. Chem. Soc.* **1964**, *86*, 4655–4663.
(42) Briggs, P. J.; Satchell, D. P. N.; White, G. F. Acylation. Part XXX. Metal Ion Catalysed Hydrolysis of Acetyl Phosphate. *J. Chem. Soc. B Phys. Org.* **1970**, No. 0, 1008–1012.
(43) Barrozo, A.; Liao, Q.; Esguerra, M.; Marloie, G.; Florián, J.; H. Williams, N.; Lynn Kamerlin, S. C. Computer Simulations of the Catalytic Mechanism of Wild-Type and Mutant β-Phosphoglucomutase. *Org. Biomol. Chem.* **2018**, *16*, 2060–2073.
(44) Bayly, C. I.; Cieplak, P.; Cornell, W.; Kollman, P. A. A Well-Behaved Electrostatic Potential Based Method Using Charge Restraints for Deriving Atomic Charges: The RESP Model. *J. Phys. Chem.* **1993**, *97*, 10269–10280.
(45) Frisch, M. J.; Trucks, G. W.; Schlegel, H. B.; Scuseria, G. E.; Robb, M. A.; Cheeseman, J. R.; Scalmani, G.; Barone, V.; Petersson, G. A.; Nakatsuji, H.; Li, X.; Caricato, M.; Marenich, A. V.; Bloino, J.; Janesko, B. G.; Gomperts, R.; Mennucci, B.; Hratchian, H. P.; Ortiz, J. V.; Izmaylov, A. F.; Sonnenberg, J. L.; Williams; Ding, F.; Lipparini, F.; Egidi, F.; Goings, J.; Peng, B.; Petrone, A.; Henderson, T.; Ranasinghe, D.; Zakrzewski, V. G.; Gao, J.; Rega, N.; Zheng, G.; Liang, W.; Hada, M.; Ehara, M.; Toyota, K.; Fukuda, R.; Hasegawa, J.; Ishida, M.; Nakajima, T.; Honda, Y.; Kitao, O.; Nakai, H.; Vreven, T.; Throssell, K.; Montgomery Jr., J. A.; Peralta, J. E.; Ogliaro, F.; Bearpark, M. J.; Heyd, J. J.; Brothers, E. N.; Kudin, K. N.; Staroverov, V. N.; Keith, T. A.; Kobayashi, R.; Normand, J.; Raghavachari, K.; Rendell, A. P.; Burant, J. C.; Iyengar, S. S.; Tomasi, J.; Cossi, M.; Millam, J. M.; Klene, M.; Adamo, C.; Cammi, R.; Ochterski, J. W.; Martin, R. L.; Morokuma, K.; Farkas, O.; Foresman, J. B.; Fox, D. J. *Gaussian 16 Rev. C.01*; Wallingford, CT, 2016.
(46) *MacroModel, Schrödinger Release 2017-1. Schrödinger: New York 2017.*
(47) Purg, M.; Elias, M.; Kamerlin, S. C. L. Similar Active Sites and Mechanisms Do Not Lead to Cross-Promiscuity in Organophosphate Hydrolysis: Implications for Biotherapeutic Engineering. *J. Am. Chem. Soc.* **2017**, *139*, 17533–17546.
(48) Blum, M.-M.; Timperley, C. M.; Williams, G. R.; Thiermann, H.; Worek, F. Inhibitory Potency against Human Acetylcholinesterase and Enzymatic Hydrolysis of Fluorogenic Nerve Agent Mimics by Human Paraoxonase 1 and Squid Diisopropyl Fluorophosphatase. *Biochemistry* **2008**, *47*, 5216–5224.
(49) Blum, M.-M.; Mustyakimov, M.; Rüterjans, H.; Kehe, K.; Schoenborn, B. P.; Langan, P.; Chen, J. C.-H. Rapid Determination of Hydrogen Positions and Protonation States of Diisopropyl Fluorophosphatase by Joint Neutron and X-Ray Diffraction Refinement. *Proc. Natl. Acad. Sci.* **2009**, *106*, 713–718.

(50) Blum, M.-M.; Löhr, F.; Richardt, A.; Rüterjans, H.; Chen, J. C.-H. Binding of a Designed Substrate Analogue to Diisopropyl Fluorophosphatase: Implications for the Phosphotriesterase Mechanism. *J. Am. Chem. Soc.* **2006**, *128*, 12750–12757.
(51) Lee, F. S.; Warshel, A. A Local Reaction Field Method for Fast Evaluation of Long-range Electrostatic Interactions in Molecular Simulations. *J. Chem. Phys.* **1992**, *97*, 3100–3107.
(52) Berendsen, H. J. C.; Postma, J. P. M.; van Gunsteren, W. F.; DiNola, A.; Haak, J. R. Molecular Dynamics with Coupling to an External Bath. *J. Chem. Phys.* **1984**, *81*, 3684–3690.
(53) Roe, D. R.; Cheatham, T. E. PTRAJ and CPPTRAJ: Software for Processing and Analysis of Molecular Dynamics Trajectory Data. *J. Chem. Theory Comput.* **2013**, *9*, 3084–3095.
(54) Lu, Z.; Dunaway-Mariano, D.; Allen, K. N. The Catalytic Scaffold of the Haloalkanoic Acid Dehalogenase Enzyme Superfamily Acts as a Mold for the Trigonal Bipyramidal Transition State. *Proc. Natl. Acad. Sci.USA* **2008**, *105*, 5687–5692.
(55) Peck, A.; Sunden, F.; Andrews, L. D.; Pande, V. S.; Herschlag, D. Tungstate as a Transition State Analog for Catalysis by Alkaline Phosphatase. *J. Mol. Biol.* **2016**, *428*, 2758–2768.
(56) Zhang, M.; Zhou, M.; Van Etten, R. L.; Stauffacher, C. V. Crystal Structure of Bovine Low Molecular Weight Phosphotyrosyl Phosphatase Complexed with the Transition State Analog Vanadate. *Biochemistry* **1997**, *36*, 15–23.
(57) X-Ray Structures of a Novel Acid Phosphatase from *Escherichia Blattae* and Its Complex with the Transition-State Analog Molybdate. *EMBO J.* **2000**, *19*, 2412–2423.
(58) Xiang, T.; Liu, Q.; Deacon, A. M.; Koshy, M.; Kriksunov, I. A.; Lei, X. G.; Hao, Q.; Thiel, D. J. Crystal Structure of a Heat-Resilient Phytase from *Aspergillus Fumigatus*, Carrying a Phosphorylated Histidine. *J. Mol. Biol.* **2004**, *339*, 437–445.
(59) Di Sabato, G.; Jencks, W. P. Mechanism and Catalysis of Reactions of Acyl Phosphates. I. Nucleophilic Reactions. *J. Am. Chem. Soc.* **1961**, *83*, 4393–4400.
(60) Muegge, I.; Tao, H.; Warshel, A. A Fast Estimate of Electrostatic Group Contributions to the Free Energy of Protein-Inhibitor Binding. *Protein Eng. Des. Sel.* **1997**, *10*, 1363–1372.
(61) Kamerlin, S. C. L.; Warshel, A. The EVB as a Quantitative Tool for Formulating Simulations and Analyzing Biological and Chemical Reactions. *Faraday Discuss.* **2010**, *145*, 71–106.
